# *Staphylococcus aureus coa* gene sequence analysis can prevent misidentification of coagulase-negative strains and contribute to their control in dairy cow herds

**DOI:** 10.3389/fmicb.2023.1120305

**Published:** 2023-05-11

**Authors:** Clara Locatelli, Stefano Gattolin, Valentina Monistero, Bianca Castiglioni, Paolo Moroni, Maria Filippa Addis, Paola Cremonesi

**Affiliations:** ^1^Department of Veterinary Medicine and Animal Sciences, Università degli Studi di Milano, Lodi, Italy; ^2^Laboratorio di Malattie Infettive degli Animali, Università degli Studi di Milano, Lodi, Italy; ^3^Italian National Research Council, Institute of Agricultural Biology and Biotechnology, Lodi, Italy; ^4^Quality Milk Production Services, Animal Health Diagnostic Center, Cornell University, Ithaca, NY, United States

**Keywords:** coagulase gene, dairy cow, genotyping, sequencing, *Staphylococcus aureus*

## Abstract

Accurate and precise differentiation of staphylococci isolated from milk is of importance for udder health management. In particular, the rapid and specific identification of *Staphylococcus aureus* plays an essential role in the prevention and treatment programs for bovine mastitis. Plasma gelatinization in coagulase assays is routinely used to discriminate *S. aureus* from other species by detecting the presence of extracellular free staphylocoagulase. However, rarely occurring coagulase-deficient *S. aureus* strains can be responsible for clinical and subclinical mastitis cases. By investigating *S. aureus* isolates from a single herd over a 10-year period we identified the persistence of a phenotypically coagulase-negative *S. aureus* strain and pinpointed the possible cause to a single base pair deletion in the *coa* gene sequence. Our results support the need to integrate primary biochemical tests with molecular/sequence analysis approaches for correctly identifying and discriminating atypical *S. aureus* in bovine herds, as the coagulase test alone may fail to detect persistent mastitis-causing strains.

## 1. Introduction

The family *Staphylococcaceae* comprises the genus *Staphylococcus*, a diverse group of gram-positive bacteria globally recognized as commensal colonizers of humans and warm-blooded animals (Founou et al., 2018). In food-producing animals, their principal reservoirs are the skin and mucosa of pigs, chickens, sheep, goats, and cows (Huber et al., 2011). Among them, the majority of species shares the inability to clot rabbit plasma and are referred to as coagulase-negative staphylococci (CoNS). Though CoNS are frequently detected in dairy cattle (De Buck et al., 2021), they are considered “minor pathogens” (Hamel et al., 2020; Ryman et al., 2021). On the other hand, coagulase-positive staphylococci (CoPS) including *Staphylococcus aureus*, *S. intermedius* and *S. hyicus*, are ubiquitous and highly versatile microorganisms implicated in a large variety of infections, ranging from dermatitis to septicemia, with *S. aureus* being the best-known of these pathogens.

In dairy cows, *S. aureus* is predominantly classified as a contagious mastitis causative agent whose presence is more frequently associated with subclinical than clinical cases, although staphylococcal infections may also occur in clinical forms (Schroeder, 2012). This microorganism is characterized by lower recovery rates than the other staphylococci, despite the efforts in controlling its presence and spread in dairy herds (Exel et al., 2022). The accurate and precise differentiation of staphylococci isolated from milk samples has therefore a major impact on udder health management. In particular, the rapid and specific identification of *S. aureus* plays an essential role in bovine mastitis prevention and treatment programs, at the point that CoNS are currently referred as Non-*aureus* Staphylococci (NAS), stressing this dichotomy. As this species differs from other staphylococcal species for being generally β-hemolytic, the hemolysis detection can also represent a fast and cost-effective method for testing the presumptive *S. aureus* presence in primary cultures, despite the low sensitivity and specificity (Ryman et al., 2021). However, previous studies demonstrated that almost 20–25% of *S. aureus* isolates from bovine intramammary infections (IMIs) show no visible β-hemolysis on blood agar plates (Ryman et al., 2021). On the other hand, as several NAS species also show β-hemolysis, selective media like Baird-Parker + RPF (Rabbit Plasma Fibrinogen) Agar or Mannitol Salt Agar (MSA) can be alternatively used for the differential growth of staphylococci (Pumipuntu et al., 2017). Nevertheless, the former works through the same principle of coagulase activity and the latter yields positive reaction also with some NAS.

Therefore, in diagnostic laboratories using primary and secondary biochemical tests for bacterial species identification, the coagulase assay is most frequently used to differentiate *S. aureus* from NAS (Peetermans et al., 2015). The bound form or clumping factor can be detected by a slide test, while extracellular free *S. aureus* coagulase (staphylocoagulase) can be detected by plasma gelatinization in a standard coagulase test tube (CTT), normally prepared from rabbit or horse whole blood (Peetermans et al., 2015). This enzyme can prime the non-proteolytic activation of prothrombin and cleavage of fibrinogen to promote coagulation (McAdow et al., 2012). Nevertheless, some *S. aureus* strains may not express this major characteristic, producing false negative results (Akineden et al., 2011). These CTT-negative *S. aureus* could therefore be erroneously classified as NAS and managed as such, with a potential for a spread within the herd (Akineden et al., 2011; Sunagar et al., 2013).

Although Matrix-Assisted Laser Desorption/Ionization Time-of-Flight Mass Spectrometry (MALDI-TOF MS) technique is being increasingly applied in veterinary microbiology, many laboratories still carry out bacterial identification with primary biochemical tests as they are easy to set up and cost-effective. In these settings, coagulase deficiencies in *S. aureus* due to transcriptional or post-transcriptional alterations in the *coa* gene (McAdow et al., 2012) may therefore lead to an erroneous species identification, with adverse effects on udder health control programs.

In our laboratory work, we repeatedly isolated coagulase-deficient *S. aureus* from clinical and subclinical mastitis cases occurring in a single herd over a 10-year period. In this study, we present the detailed characterization of the *coa* gene of the coagulase-negative *S. aureus* strain isolated from this dairy farm with the aim of understanding the molecular basis for this phenotypic behavior and suggesting mitigation measures to possible identification bias.

## 2. Materials and methods

### 2.1. Sample collection and *S. aureus* identification

During the daily diagnostic activity at the Laboratorio di Malattie Infettive degli Animali (MiLab, Università degli Studi di Milano, Italy), from December 2013 to August 2022, a total of 31 staphylococcal isolates were obtained from as many milk samples from a single Italian dairy farm and presumptively identified as *S. aureus*. The samples (15 quarter clinical mastitis, 14 composite and 2 quarter milk samples with high somatic cell count, Table 1) were cultured and evaluated according to National Mastitis Council procedures (National Mastitis Council, 2017), including CTT (Pumipuntu et al., 2017). The typical colony morphology and the beta-hemolysis on blood agar suggested to subculture the isolates onto Mannitol Salt Agar (MSA, Oxoid, Basingstoke, United Kingdom) and Baird Parker with Rabbit Plasma (BP + RPF, Microbiol, Cagliari, Italy) for further characterization. All the isolates were frozen in Nutrient Broth (Microbiol, Cagliari, Italy) added with 15% glycerol (Carlo Erba Reagents, Milan, Italy) until further analyses.

**TABLE 1:** *Staphylococcus aureus* isolates collected from a single herd analyzed in this study.

No.	Isolates	Type of sample	Isolation date	Cow	Coagulase test	MALDI-TOF MS log score	Genotype
1	2948	Composite	10/12/2013	169	Negative	2.43	GTBN
2	2949	Clinical mastitis	10/12/2013	259 RR	Negative	2.28	GTBN
3	2937	Composite	28/10/2013	274	Negative	2.19	GTBN
4	2936	Composite	28/10/2013	169	Negative	2.39	GTBN
5	2947	Composite	10/12/2013	286	Negative	2.15	GTBN
6	2976	Composite	17/04/2014	297	Negative	2.32	GTBN
7	2977	Composite	17/04/2014	55	Negative	2.24	GTBN
8	2978	Composite	17/04/2014	213	Negative	2.37	GTBN
9	2979	Clinical mastitis	17/04/2014	169 FR	Negative	2.22	GTBN
10	2980	Clinical mastitis	17/04/2014	213 RR	Negative	2.21	GTBN
11	3023	Clinical mastitis	24/09/2014	354	Negative	2.47	GTBN
12	3119	Clinical mastitis	09/12/2015	161 FR	Negative	2.56	GTBN
13	3209	Composite	31/01/2017	316	Negative	2.33	GTBN
14	3245	Clinical mastitis	20/06/2017	245 FL	Negative	2.27	GTBN
15	3254	Clinical mastitis	10/10/2017	223	Negative	2.45	GTBN
16	3259	Quarter milk	15/01/2018	223	Negative	2.15	GTBN
17	3260	Quarter milk	15/01/2018	223	Negative	2.36	GTBN
18	3263	Clinical mastitis	05/06/2018	139 FL	Negative	2.25	GTBN
19	3264	Clinical mastitis	05/06/2018	312 RL	Negative	2.32	GTBN
20	3265	Composite	05/06/2018	223	Negative	2.54	GTBN
21	3277	Clinical mastitis	07/05/2019	156	Positive	2.50	GTA^I^
22	3282	Composite	16/07/2019	26	Positive	2.42	GTA^I^
23	3283	Composite	16/07/2019	61	Negative	2.31	GTBN
24	3473	Clinical mastitis	28/06/2022	21	Positive	2.19	GTA^I^
25	3474	Clinical mastitis	28/06/2022	23	Positive	2.11	GTA^I^
26	3482	Composite	22/08/2022	409	Negative	2.20	GTBN
27	3483	Clinical mastitis	22/08/2022	409	Negative	2.14	GTBN
28	56	Composite	22/08/2022	321	Positive	2.34	GTA
29	88	Composite	22/08/2022	299	Positive	2.30	GTA
30	117	Clinical mastitis	08/08/2022	37 FL	Positive	2.30	GTA
31	125	Clinical mastitis	08/08/2022	270 FR	Positive	2.36	GTA

### 2.2. DNA extraction and molecular identification

DNA was extracted using the DNA isolation system kit (Clonit, Medical System, Genova, Italy) according to the protocol described by Cremonesi et al. (2006), starting from step 2. DNA quality and quantity were measured using a NanoDrop ND-1000 spectrophotometer (Nano-Drop Technologies, Wilmington, DE); the samples were stored at −20°C until further use.

To check the specificity of the isolates, the DNA extracted was amplified with *nuc* (thermonuclease coding gene) and *coa* (coagulase coding gene) primers, as previously described (Cremonesi et al., 2005). As positive controls, *S. aureus* reference strains (ATCC 19040, ATCC 19041, ATCC 19048, ATCC 700699) were used in each PCR assay. All amplified PCR fragments were separated by 2% agarose gel electrophoresis (GellyPhor, Euroclone, Milan, Italy), stained with ethidium bromide (0.05 mg/mL; Sigma-Aldrich), and visualized under UV transilluminator (BioView Ltd., Nes Ziona, Israel). A 100-bp DNA ladder (Finnzymes, Espoo, Finland) was included in each gel.

### 2.3. Confirmation through MALDI-TOF MS

MALDI-TOF MS (Bruker Daltonik GmbH, Bremen, Germany) has been introduced in routine mastitis diagnostics at MiLab since January 2021. All the isolates were analyzed following the protocol described in Monistero et al. (2021) and Rosa et al. (2022).

### 2.4. RS-PCR typing

All the 31 isolates were also genotyped by RS-PCR as previously described with a detailed working protocol (Fournier et al., 2008; Graber, 2016). The method is based on the amplification of the 16S–23S rRNA intergenic spacer region. The PCR products were analyzed using the miniaturized electrophoresis system DNA 7500 LabChip (Agilent Technologies, Santa Clara, CA). Genotypes were inferred from the electrophoresis profile using the Mahal software, which is freely available online (Graber, 2016).^1^

### 2.5. Library preparation, sequencing, bioinformatics analysis

The DNA was amplified for the entire coagulase gene by using the following primers designed by the Primer3 programme^2^ : FORWARD = GCCGCTTTAATACCAGCAAC; REVERSE = CTTCCGATTGTTCGATGCTT (amplicon size = 2268 bp). The PCR amplifications were performed in 25 μl volumes per sample. A total of 12.5 μl of GoTaq^®^ Long PCR Master Mix, 2X (Promega Corporation, Madison, USA) and 0.2 μl of each primer (100 μM) were added to 2 μl of genomic DNA (5 ng/μl). A first amplification step was performed in an Applied Biosystem 2700 thermal cycler (ThermoFisher Scientific). Samples were denatured at 95°C for 2 min, followed by 30 cycles with a denaturing step at 94°C for 30 s, annealing at 55°C for 30 s and extension at 68°C for 2 min, with a final extension at 72°C for 10 min. The amplicons were then cleaned with Agencourt AMPure XP (Beckman, Coulter Brea, CA, USA) and libraries were prepared following the Nextera XT DNA library Prep Kit Protocol (Illumina, San Diego, CA, USA), using a tagmentation of 15 min with *Pdm*I enzyme. The libraries obtained were quantified by Real Time PCR with KAPA Library Quantification Kits (Kapa Biosystems, Inc., MA, USA), pooled in equimolar proportion and sequenced in one MiSeq (Illumina) run with 2 × 300-base paired-end reads. FASTQ files were mapped with BWA-MEM2 against staphylocoagulase GenBank locus sequence LOCUS JN861807 (strain MSSA_129) on the Galaxy Platform (Afgan et al., 2018) and reads coverage was visualized using IGV (Robinson et al., 2011).

### 2.6. Molecular phylogenetic analysis by the neighbor-joining method

CDS DNA sequences used for phylogenetic analysis from different *S. aureus* strains and subspecies were obtained from the NCBI database.^3^ Phylogenetic relationships were estimated in MEGAX (Kumar et al., 2018). DNA sequences (Supplementary material) were aligned by MUSCLE with default settings. Where a frameshift mutation was present, the whole sequence was used to allow for a full length alignment. Evolutionary relationships among coagulase sequences were inferred by using the Neighbor-Joining method and evolutionary distances were computed using the Maximum Composite Likelihood method and are in the units of the number of base substitutions per site. The rate variation among sites was modeled with a gamma distribution. The reliability of the phylogenetic tree was estimated by setting 1000 bootstrap replicates.

### 2.7. Sanger sequencing

All the samples were also analyzed by conventional sanger sequencing. The DNA was amplified for the coagulase gene by using the following primers designed in conserved regions: FORWARD = ATGGGATAACAAAGCAGATG; REVERSE = GGTTCTTCAACTTTCTTCTC (amplicon size = 900 bp). The PCR amplifications were performed in 25 μl volumes per sample. A total of 12.5 μl of PCR Master Mix, 2X (Thermofisher Scientific) and 0.2 μl of each primer (100 μM) were added to 2 μl of genomic DNA (5 ng/μl). The amplification step was performed in an Applied Biosystem 2700 thermal cycler (ThermoFisher Scientific). Samples were denatured at 95°C for 2 min, followed by 30 cycles with a denaturing step at 95°C for 1 min, annealing at 54°C for 1 min and extension at 72°C for 1 min, with a final extension at 72°C for 10 min. The amplicons were then cleaned with Wizard^®^ SV Gel and PCR Clean-Up System (Promega Italia, Milan, Italy) and the cleaned products were sequenced by Eurofins Genomics (Ebersberg, Germany), following the instructions of the manufacturer.

## 3. Results and discussion

### 3.1. Bacteriological analysis and coagulase test

As previously described (Akineden et al., 2011), the discrimination between CoPS and CoNS catalase-positive cocci represents one of the most important routine procedures for identifying the etiological agents of contagious mastitis within a dairy herd. Coagulase is considered a virulence factor and staphylococci that are not able to produce this protein are reported to be less pathogenic. This is why the CTT for *S. aureus* coagulase activity remains the reference and the most used method in the laboratory routine. Although rare, some atypical *S. aureus* strains may occur (Akineden et al., 2011; Sunagar et al., 2013) and coagulase test specificity should be considered to avoid misclassification. From bacteriological analysis, 23 isolates (10 quarter clinical mastitis, 11 composite and 2 quarter high somatic cell samples, Table 1) resulted unexpectedly negative at the coagulase test after both 4 and 24 h of incubation at 37°C. The 23 CTT-negative isolates found in this study demonstrated the importance of applying appropriate screening testing to avoid *S. aureus* misclassification and to prevent staphylococcal infection spread within farms. The remaining 8 *S. aureus* analyzed were CTT-positive isolates producing a typical coagulase halo around the colonies on BP-RPF demonstrating lecithinase activity, whereas this was weak to absent for the 23 CTT-negative isolates. Aside from CTT results, all 31 *S. aureus* isolates evaluated in this study showed a positive reaction on MSA, proving the higher sensitivity of this selective medium compared to BP-RPF. As an initial screening method and compared to CTT, using MSA represents a low cost good choice for a more sensitive and still acceptably rapid *S. aureus* identification. However, due to the limited specificity, *S. aureus* identified through MSA positive results should be confirmed by further molecular methods (Pumipuntu et al., 2017).

### 3.2. Molecular characterization: amplification of *nuc* and *coa* genes and RS-PCR analysis

The identification as *S. aureus* was also confirmed by MALDI-TOF results, with scores >2.00 for all the isolates (Table 1). A misidentified *S. aureus* due to a negative CTT could drive to severe consequences as the other virulence factors activity (i.e., enterotoxins) could be conserved. The MALDI-TOF identification was further supported by PCR on the thermonuclease (*nuc*) and coagulase (*coa*) genes for all the 31 isolates evaluated in this study. Moreover, the RS-PCR carried out for verification of the genotypes circulating in the herd in the time frame evaluated in this study revealed that all the 23 coagulase-negative *S. aureus* belonged to genotype GTBN (Table 1), while the 8 coagulase-positive *S. aureus* belonged to genotypes GTA (*N* = 6 in 2022) and GTA^I^ (*n* = 2 in 2019). Previous studies (Cremonesi et al., 2015; Cosandey et al., 2016) using the RS-PCR technique, however, demonstrated that *S. aureus* isolated from bovine IMIs are genetically heterogeneous, with some *S. aureus* genotypes showing a limited tendency to spread in the herd, as for GTBN, GTA and GTA^I^. The knowledge of the characteristics of *S. aureus* circulating in the herd might help to formulate strategies for focused treatment and control of disease (Monistero et al., 2018).

### 3.3. Sequencing of the *coa* gene

Out of the 23 isolates with the same GTBN genotype, 4 (isolates number 1, 4, 5, 12) were randomly chosen and analyzed in one Miseq run in order to determine the nucleotide sequence of the *coa* gene. As previously described (Watanabe et al., 2005, 2009; Johler et al., 2012), this gene is known to be divided into six regions: the signal sequence, the D1 and D2 regions enabling contact with prothrombin, the central region, a repeat region, and the C-terminal sequence.

**FIGURE 1:**
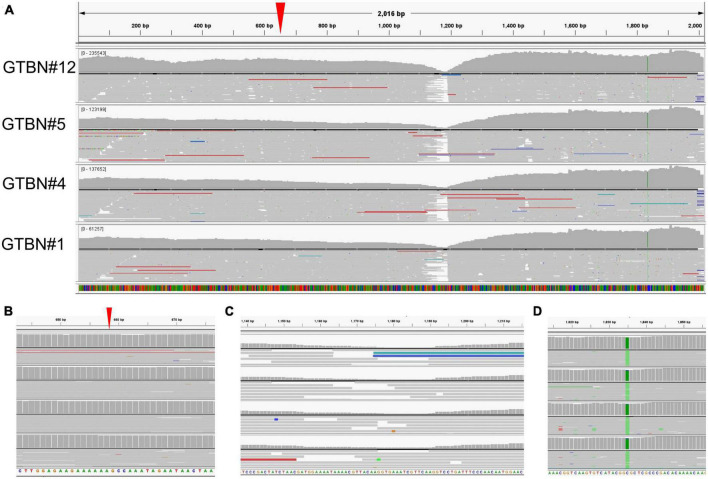
Molecular analysis of sequence variants observed in the GTBN coagulase genes. **(A)** Sequencing reads obtained from isolates GTBN#1, 4, 5, and 12 were mapped using MSSA_129 as reference sequence. Gray signals correspond to sequence identity with MSSA_129. The boxes show in more detail **(B)** the position of the single A deletion at position 653 bp in both the reference and the four samples (red arrowhead), **(C)** an area with drop in read coverage at around 1,165–1,190 and **(D)** a SNP (C to A) at position 1,835 common to all sequenced samples.

In order to choose a suitable reference for mapping, preliminary BLAST searches using individual reads from the FASTQ sequencing files were carried out against *S. aureus* from the GenBank database. Since all the reads used aligned to strain MSSA_129 (sequence JN861807, Johler et al., 2012), this strain was used as reference. The *coa* gene from the methicillin-susceptible *S. aureus* MSSA_129 strain was previously reported to harbor a single base deletion at position 653 within the D2 region of the gene, leading to a frameshift and premature termination of the resulting protein (Johler et al., 2012). The four GTBN *S. aureus* strains revealed the same sequence (Figure 1A), and complete identity to MSSA_129 around position 653 in the D2 region of the gene (Figure 1B and Supplementary material 2A). This was further confirmed in all the 23 GTBN isolates by Sanger sequencing, by using primers chosen in conserved regions between the sequences from GTBN of interest (isolates 1, 4, 5, 12) and sequences from non-GTBN genotypes (NCTC7485, ATCC6538, SA1428). Conversely, the deletion at position 653 was not observed in the GTA and GTA^I^ coagulase sequences. As the MSSA_129 strain (Johler et al., 2012), also all our GTBN samples would therefore be characterized by a premature stop codon resulting in a predicted 223 aa peptide that is most likely not functional. By inspecting the rest of the sequence, no differences to the reference used were found in the central portion of the gene, not even at around 1,165–1,190 bp where fewer reads were available to ensure coverage (Figure 1C). Finally, sequence comparison revealed that the four GTBN strains could be genetically differentiated from the reference used (MSSA_129) by the presence of a SNP, (C to A) at position 1,835 (Figure 1D).

**FIGURE 2:**
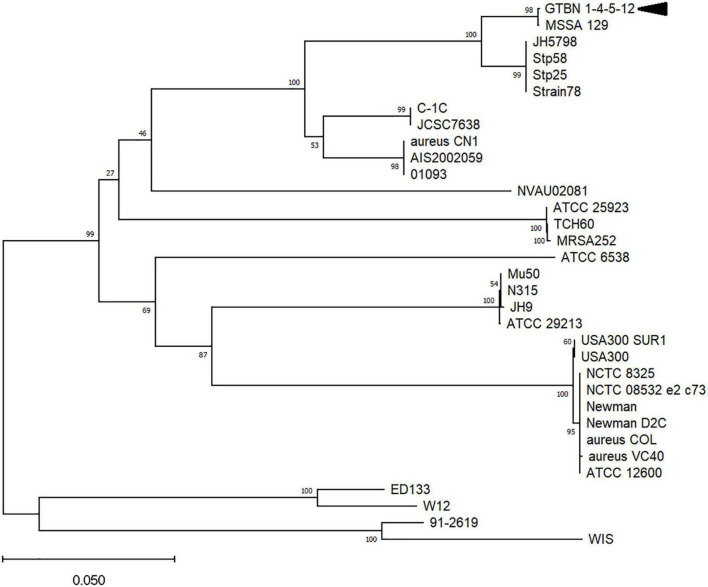
Evolutionary relationships among coagulase genes in different *Staphylococcus aureus* strains and subspecies. Only bootstrap values >50 are shown. The arrowhead indicates the sequence corresponding to the four isolates (GTBN#1, 4, 5, and 12).

To gain insights into the variability of *coa* genes from different *S. aureus* strains and subspecies, the sequence from the GTBN isolates and that of MSSA_129 were aligned to 21 *coa* CDS obtained from GenBank (listed in Supplementary material), and their relationships were inferred by using the Neighbor-Joining method (Figure 2). This analysis showed that the sequences were separated in different clusters, and that those of GTBN isolates and MSSA_129 clustered closely, among others, with the two sequences Stp58 and Stp25 (Watanabe et al., 2009). These two were CTT-positive MSSA isolated in Japan (Watanabe et al., 2009). The Stp58 *coa* sequence was identical to the Stp25 *coa* sequence; their alignment revealed the lack of the single base deletion at position 653 bp observed in the 4 GTBN samples from this study (Supplementary material 2B), resulting in a predicted full length peptide in these two strains (Supplementary material 2C). Since Stp58 and Stp25 were reported as CTT-positive, this is further evidence that the base deletion is responsible for the lack of coagulase activity in GTBN 1, 4, 5, and 12.

The collection of samples over a 10-years period suggested the persistence of the low diffusive GTBN in the same herd; all these coagulase negative strains were characterized by an indel-mutation in *coa* gene. This study has potential limitations as analysis by using whole genome sequencing (WGS), cheaper today than few years ago, would provide deeper information on the atypical *S. aureus* circulating in bovine herds and allow to inspect the sequences of genes other than coagulase. However, the use of gene sequence analysis is nowadays still far from being a routine method, and in this specific case study this approach was enough to provide an answer to the issue encountered. As no single phenotypic test can guarantee reliable results in *S. aureus* detection, our work emphasizes the usefulness of MALDI-TOF or molecular methods in routine analyses to identify and discriminate atypical staphylococci from bovine mastitis cases. Where such advanced identification techniques are not yet available, combining the results of more than one test is crucial to avoid misidentification due to atypical strains. The knowledge about the epidemiology of coagulase–negative *S. aureus* genotypes might support control measures directed to reduce the spread of a contagious pathogen within dairy herds.

## Data availability statement

The datasets presented in this study can be found in online repositories. The names of the repository/repositories and accession number(s) can be found below: NCBI SRA – SAMN32298094.

## Author contributions

CL and PC designed and performed the experiments. SG helped with the data analysis. BC, PM, and MA gave advices to the researchers. VM, CL, SG, MA, and PC wrote the manuscript. All authors critically reviewed the manuscript and approved the final version.

## Footnotes

1
https://mahal.vitech.dev/#/

2
https://primer3.ut.ee/

3
https://www.ncbi.nlm.nih.gov/
