# Supplementary material for: Staphylococcus aureus coa gene sequence analysis can prevent misidentification of coagulase-negative strains and contribute to their control in dairy cow herds
Source: Front Microbiol. 2023 May 11;14:1120305. doi: 10.3389/fmicb.2023.1120305 (PMC10213915; doi:10.3389/fmicb.2023.1120305)
Supplement: Supplementary file 1 [file Data_Sheet_1.docx]

Supplementary Material

*Staphylococcus aureus coa* gene sequence analysis can prevent misidentification of coagulase-negative strains and contribute to their control in dairy cow herds

Clara Locatelli^1,2†^, Stefano Gattolin^3†^, Valentina Monistero^1,2^, Bianca Castiglioni^3^, Paolo Moroni^1,2,4^, Maria Filippa Addis^1,2^, Paola Cremonesi^3*^

**Correspondence:** Paola Cremonesi: paola.cremonesi@ibba.cnr.it

# Supplementary Data

**List of CDS DNA sequences used for phylogenetic analysis from different *S. aureus* strains and subspecies were obtained from the NCBI database**

| **STRAIN** | **Genbank** |
| --- | --- |
| MSSA129 | JN861807.1 |
| JH5798 | LC547337.1 |
| Strain 78 | CP022682.1 |
| ATCC_25923 | CP009361.1 |
| NCTC 8325 | NC_007795.1 |
| Mu50 | BA000017.4 |
| ATCC_6538 | CP020020.1 |
| ATCC 29213 | CP094857.1 |
| USA300 SUR1 | CP009423.1 |
| NCTC 08532_e2_c73 | PPRK01000035.1 |
| MRSA252 | BX571856.1 |
| Newman | AP009351.1 |
| Newman_D2C | CP023391.1 |
| N315 | BA000018.3 |
| JH9 | CP000703.1 |
| Aureus_COL | CP000046.1 |
| Aureus_VC40 | CP003033.1 |
| USA300 | CP092052.1 |
| TCH60 | CP002110.1 |
| ED133 | CP001996.1 |
| Aureus_CN1 | CP003979.1 |
| ATCC_12600 | CP035101.1 |
| Stp58 | AB489894.1 |
| Stp25 | AB489895.1 |
| C-1C | AB436977.1 |
| JCSC 7638 | AB488509.1 |
| AIS2002059 | AB436979.1 |
| 01093 | AB436978.1 |
| W12 | AB489890.1 |
| 91/2619 | AB489901.1 |
| WIS | AB489893.1 |
| NVAU02081 | AB436985.1 |

>GTBN_1-4-5-12

ATGAAAAAGCAAATAATTTCGCTAGGCGCATTAGCAGTTGCATCTAGCTTATTTACATGGGATAACAAAGCAGATGCGATAGTAACAAAGGATTATAGTAAAGAATCAAGAGTGAAAGAGAACAGTAAATATGATTCGCCAATGTCAAATTGGTATTATTGGGGAAAGGTTAAATCCTTGGAGTCACAATTTGCAGATGCAATAGATATTATAGAAGATTATCAATATGGTGAAAAAGAATATAAAGATGCAAAAGATAAACTAATGACTAGAATACTAGGTGAGGACCAATACTTATTAAAGAAAAAAATAGAAGAATATAAACAATATAGAGAAAGATATTTAAAAGCTGGATTAAGTCCTGTGAAATTTTATGATTACAATCTTTATGATTTTACAATGAAAGAATATAATGATATCCATCAGTCTTTAAAAGATGCAGTAGAAGAGTTCTATCAAGAAGTTAAACATATTCAATCAAAGAATTCGGATTTACAAACTTATGATAAGAAAACTGAAGATAAAGAAACTGATAATGTATACTCTTTAGTTAGTGAAATTGATACTATTGTTGCAACATATTATGGAGATAAAAATCATGGAGAGCATGCTAAAGAGTTGAGAGCTAAGCTAGATATTATTCTTGGAGAAGAAAAAAGCCAAATAGAATAACTAATGAACGTATTAGAAAAGAAATGACTGATGATTTGAATTCTATTATCGATGACTTCTTTATGGAAACTGGGCAAAACAGACCGGTTAAAATCACTAAATATAATCCAAATATTCATAGCCCTAAAGATAACAAAGAAAGCTTCGATAAATTAGTTGAAGAAACGAAAAAAGCAGTTAAAGAAGCAGATGAGTCTTGGAAAACTAAAACTGTCAAAACATACGGTGAAACTGAAACAAAAGCACATGTTGTAAAAGAAGAGAAGAAAGTTGAAGAACCTCAATTACCTAAAGTTGGAAACCAGCAAGAGGATAAAACTACAGTTGGTACAACTGAAAAAGCACCATTACCAATTGCGCAACCACTAGTTAAATTACCACAAATTGGGACTCAAGGTAAAATTGTAGAAGGGCCAAAATACCCAACGATGGAACAGCACACAATCTATGGTGAAATTGTAAATGGTCCCGACTATCTAACGATGGAAAATAAAACGTTACAAGGTGAAATCGTTCAAGGTCCTGATTTCCCAACAATGGAACAAAACAGACCATCTTTAAGCGATAATTATACTCAACCGACGACACCGAACCCTATTTTAGAAGGTCTTGAAGGTAGCTCATCTAAACTCGAAATAAAACCACAAGGTACTGAATCAACGTTGAAAGGTATTCAAGGAGAATCAAGTGATATTGAAGTTAAACCTCAAGCAACTGAAACAACAGAAGCATCACATTATCCAGCGAGACCGCAATTTAACAAAACACCTAAGTATGTGAAATATAGAGATGCTGGTACAGGTATTCGTGAATACAACGATGGAACATTTGGATATGAAGCGAGACCAAGATTCAACAAGCCAAGTGAAACAAATGCATACAACGTAACGACAAATCAAGATGGCACAGTATCATACGGCGCCCGCCCGACACAAAACAAGCCAAGCGAAACGAATGCATATAACGTAACAGCACACGGAAATGGTCAAGTGTCATACGGCGCTCGTCCGACATACAAGAAGCCAAGCGAAACAAATGCATATAACGTAACAACACATGCAAATGGTCAAGTATCATACGGAGCTCGCCCAACACAAAACAAGCCAAGCGAAACAAACGCATATAACGTAACAACACATGCAAACGGTCAAGTGTCATACGGAGCTCGCCCGACACAAAACAAGCCAAGCAAAACAAACGCATATAACGTAACAACACACGGAAATGGCACAGTATCATATGGCGCTCGCCCGACACAAAACAAGCCAAGTAAAACAAATGCATATAACGTAACAACACATGCAGATGGTACTGCGACATATGGTCCTAGAGTAACAAAATAA

>MSSA_129

ATGAAAAAGCAAATAATTTCGCTAGGCGCATTAGCAGTTGCATCTAGCTTATTTACATGGGATAACAAAGCAGATGCGATAGTAACAAAGGATTATAGTAAAGAATCAAGAGTGAAAGAGAACAGTAAATATGATTCGCCAATGTCAAATTGGTATTATTGGGGAAAGGTTAAATCCTTGGAGTCACAATTTGCAGATGCAATAGATATTATAGAAGATTATCAATATGGTGAAAAAGAATATAAAGATGCAAAAGATAAACTAATGACTAGAATACTAGGTGAGGACCAATACTTATTAAAGAAAAAAATAGAAGAATATAAACAATATAGAGAAAGATATTTAAAAGCTGGATTAAGTCCTGTGAAATTTTATGATTACAATCTTTATGATTTTACAATGAAAGAATATAATGATATCCATCAGTCTTTAAAAGATGCAGTAGAAGAGTTCTATCAAGAAGTTAAACATATTCAATCAAAGAATTCGGATTTACAAACTTATGATAAGAAAACTGAAGATAAAGAAACTGATAATGTATACTCTTTAGTTAGTGAAATTGATACTATTGTTGCAACATATTATGGAGATAAAAATCATGGAGAGCATGCTAAAGAGTTGAGAGCTAAGCTAGATATTATTCTTGGAGAAGAAAAAAGCCAAATAGAATAACTAATGAACGTATTAGAAAAGAAATGACTGATGATTTGAATTCTATTATCGATGACTTCTTTATGGAAACTGGGCAAAACAGACCGGTTAAAATCACTAAATATAATCCAAATATTCATAGCCCTAAAGATAACAAAGAAAGCTTCGATAAATTAGTTGAAGAAACGAAAAAAGCAGTTAAAGAAGCAGATGAGTCTTGGAAAACTAAAACTGTCAAAACATACGGTGAAACTGAAACAAAAGCACATGTTGTAAAAGAAGAGAAGAAAGTTGAAGAACCTCAATTACCTAAAGTTGGAAACCAGCAAGAGGATAAAACTACAGTTGGTACAACTGAAAAAGCACCATTACCAATTGCGCAACCACTAGTTAAATTACCACAAATTGGGACTCAAGGTAAAATTGTAGAAGGGCCAAAATACCCAACGATGGAACAGCACACAATCTATGGTGAAATTGTAAATGGTCCCGACTATCTAACGATGGAAAATAAAACGTTACAAGGTGAAATCGTTCAAGGTCCTGATTTCCCAACAATGGAACAAAACAGACCATCTTTAAGCGATAATTATACTCAACCGACGACACCGAACCCTATTTTAGAAGGTCTTGAAGGTAGCTCATCTAAACTCGAAATAAAACCACAAGGTACTGAATCAACGTTGAAAGGTATTCAAGGAGAATCAAGTGATATTGAAGTTAAACCTCAAGCAACTGAAACAACAGAAGCATCACATTATCCAGCGAGACCGCAATTTAACAAAACACCTAAGTATGTGAAATATAGAGATGCTGGTACAGGTATTCGTGAATACAACGATGGAACATTTGGATATGAAGCGAGACCAAGATTCAACAAGCCAAGTGAAACAAATGCATACAACGTAACGACAAATCAAGATGGCACAGTATCATACGGCGCCCGCCCGACACAAAACAAGCCAAGCGAAACGAATGCATATAACGTAACAGCACACGGAAATGGTCAAGTGTCATACGGCGCTCGTCCGACATACAAGAAGCCAAGCGAAACAAATGCATATAACGTAACAACACATGCAAATGGTCAAGTATCATACGGAGCTCGCCCAACACAAAACAAGCCAAGCGAAACAAACGCATATAACGTAACAACACATGCAAACGGTCAAGTGTCATACGGCGCTCGCCCGACACAAAACAAGCCAAGCAAAACAAACGCATATAACGTAACAACACACGGAAATGGCACAGTATCATATGGCGCTCGCCCGACACAAAACAAGCCAAGTAAAACAAATGCATATAACGTAACAACACATGCAGATGGTACTGCGACATATGGTCCTAGAGTAACAAAATAA

>JH5798

ATGAAAAAGCAAATAATTTCGCTAGGCGCATTAGCAGTTGCATCTAGCTTATTTACATGGGATAACAAAGCAGATGCGATAGTAACAAAGGATTATAGTAAAGAATCAAGAGTGAATGAGAACAGTAAATATGATTCGCCAATGTCAAATTGGTATTATTGGGGAAAGGTTAAATCCTTGGAGTCACAATTTGCAGATGCAATAGATATTATAGAAGATTATCAATATGGTGAAAAAGAATATAAAGATGCAAAAGATAAACTAATGACTAGAATACTAGGTGAGGACCAATACTTATTAAAGAAAAAAATAGAAGAATATAAACAATATAGAGAAAGATATTTAAAAGCTGGATTAAGTCCTGTGAAATTTTATGATTACAATCTTTATGATTTTACAATGAAAGAATATAATGATATCCATCAGTCTTTAAAAGATGCAGTAGAAGAGTTCTATCAAGAAGTTAAACATATTCAATCAAAGAATTCGGATTTACAAACTTATGATAAGAAAACTGAAGATAAAGAAACTGATAATGTATACTCTTTAGTTAGTGAAATTGATACTATTGTTGCAACATATTATGGAGATAAAAATCATGGAGAGCATGCTAAAGAGTTGAGAGCTAAGCTAGATATTATTCTTGGAGAAGAAAAAAAGCCAAATAGAATAACTAATGAACGTATTAGAAAAGAAATGACTGATGATTTGAATTCTATTATCGATGACTTCTTTATGGAAACTGGGCAAAACAGACCGGTTAAAATCACTAAATATAATCCAAATATTCATAGCCCTAAAGATAACAAAGAAAGCTTCGATAAATTAGTTGAAGAAACGAAAAAAGCAGTTAAAGAAGCAGATGAGTCTTGGAAAACTAAAACTGTCAAAACATACGGTGAAACTGAAACAAAAGCACATGTTGTAAAAGAAGAGAAGAAAGTTGAAGAACCTCAATTACCTAAAGTTGGAAACCAGCAAGAGGATAAAACTACAGTTGGTACAACTGAAAAAGCACCATTACCAATTGCGCAACCACTAGTTAAATTACCACAAATTGGGACTCAAGGTAAAATTGTAGAAGGGCCAAAATACCCAACGATGGAACAGCACACAATCTATGGTGAAATTGTAAAAGGTCCCGACTATCTAACGATGGAAAATAAAACGTTACAAGGTGAAATCGTTCAAGGTCCAGATTTCCCAACAATGGAACAAAACAGACCAGCACTAAGCGATAATTATACAAACCCAACGTTAACGAACCCTATTTTAAAAGGTATTGAAGGAAACTCAACTAATCTTGAAATAAAACCACAAGGTACTGAATCAACGTTGAAAGGTATTCAAGGAGAATCAAGTGATATTGAAGTTAAACCTCAAGCAACTGAAACAACAGAAGCATCACATTATCCAGCGAGACCGCAATTTAACAAAACACCTAAGTATGTGAAATATAGAGATGCTGGTACAGGTATCCGTGAATACAACGATGGAACATTTGGATATGAAGCGAGACCAAGATTCAACAAGCCATCAGAAACAAACGCATACAACGTAATGACAAATCAAGATGGCACAGTATCATACGGCGCCCGCCCAACACAAAACAAGGCATCAGAAACAAACGCATATAACGTAACAACACATGCAAACGGCCAAGTATCATACGGAGCTCGCCCAACACAAAAGAAGCCAAGCGAAACAAATGCATATAACGTAACAACACATGCAAACGGCCAAGTATCATATGGCGCCCGCCCGACATACAAGAAGCCAAGTGAAACAAATGCATATAACGTAACAACACATGCAAATGGCCAAGTATCATATGGGGCTCGCCCAACACAAAACAAGCCAAGCAATACAAACGCATATAACGTAACAACACATGCAAACGGCCAAGTATCATATGGGGCTCGCCCGACACAAAACAAGGCATCAGAAACAAACGCATATAACGTAACAACACATGCAAACGGCCAAGTATCATACGGAGCTCGCCCGACACAAAACAAGCCAAGCGAAACAAACGCATATAACGTAACAACACACGGAAACGGTCAAGTGTCATACGGCGCTCGTCCGACATACAACAAGCCAAGTAAAACAAATGCATACAATGTAACAACACATGCAGATGGTACTGCGACATATGGTCCTAGAGTAACAAAATAA

>Strain78

ATGAAAAAGCAAATAATTTCGCTAGGCGCATTAGCAGTTGCATCTAGCTTATTTACATGGGATAACAAAGCAGATGCGATAGTAACAAAGGATTATAGTAAAGAATCAAGAGTGAATGAGAACAGTAAATATGATTCGCCAATGTCAAATTGGTATTATTGGGGAAAGGTTAAATCCTTGGAGTCACAATTTGCAGATGCAATAGATATTATAGAAGATTATCAATATGGTGAAAAAGAATATAAAGATGCAAAAGATAAACTAATGACTAGAATACTAGGTGAGGACCAATACTTATTAAAGAAAAAAATAGAAGAATATAAACAATATAGAGAAAGATATTTAAAAGCTGGATTAAGTCCTGTGAAATTTTATGATTACAATCTTTATGATTTTACAATGAAAGAATATAATGATATCCATCAGTCTTTAAAAGATGCAGTAGAAGAGTTCTATCAAGAAGTTAAACATATTCAATCAAAGAATTCGGATTTACAAACTTATGATAAGAAAACTGAAGATAAAGAAACTGATAATGTATACTCTTTAGTTAGTGAAATTGATACTATTGTTGCAACATATTATGGAGATAAAAATCATGGAGAGCATGCTAAAGAGTTGAGAGCTAAGCTAGATATTATTCTTGGAGAAGAAAAAAAGCCAAATAGAATAACTAATGAACGTATTAGAAAAGAAATGACTGATGATTTGAATTCTATTATCGATGACTTCTTTATGGAAACTGGGCAAAACAGACCGGTTAAAATCACTAAATATAATCCAAATATTCATAGCCCTAAAGATAACAAAGAAAGCTTCGATAAATTAGTTGAAGAAACGAAAAAAGCAGTTAAAGAAGCAGATGAGTCTTGGAAAACTAAAACTGTCAAAACATACGGTGAAACTGAAACAAAAGCACATGTTGTAAAAGAAGAGAAGAAAGTTGAAGAACCTCAATTACCTAAAGTTGGAAACCAGCAAGAGGATAAAACTACAGTTGGTACAACTGAAAAAGCACCATTACCAATTGCGCAACCACTAGTTAAATTACCACAAATTGGGACTCAAGGTAAAATTGTAGAAGGGCCAAAATACCCAACGATGGAACAGCACACAATCTATGGTGAAATTGTAAAAGGTCCCGACTATCTAACGATGGAAAATAAAACGTTACAAGGTGAAATCGTTCAAGGTCCAGATTTCCCAACAATGGAACAAAACAGACCAGCACTAAGCGATAATTATACAAACCCAACGTTAACGAACCCTATTTTAAAAGGTATTGAAGGAAACTCAACTAATCTTGAAATAAAACCACAAGGTACTGAATCAACGTTGAAAGGTATTCAAGGAGAATCAAGTGATATTGAAGTTAAACCTCAAGCAACTGAAACAACAGAAGCATCACATTATCCAGCGAGACCGCAATTTAACAAAACACCTAAGTATGTGAAATATAGAGATGCTGGTACAGGTATCCGTGAATACAACGATGGAACATTTGGATATGAAGCGAGACCAAGATTCAACAAGCCATCAGAAACAAACGCATACAACGTAATGACAAATCAAGATGGCACAGTATCATACGGCGCCCGCCCAACACAAAACAAGGCATCAGAAACAAACGCATATAACGTAACAACACATGCAAACGGCCAAGTATCATACGGAGCTCGCCCAACACAAAAGAAGCCAAGCGAAACAAATGCATATAACGTAACAACACATGCAAACGGCCAAGTATCATATGGCGCCCGCCCGACATACAAGAAGCCAAGTGAAACAAATGCATATAACGTAACAACACATGCAAATGGCCAAGTATCATATGGGGCTCGCCCAACACAAAACAAGCCAAGCAATACAAACGCATATAACGTAACAACACATGCAAACGGCCAAGTATCATATGGGGCTCGCCCGACACAAAACAAGGCATCAGAAACAAACGCATATAACGTAACAACACATGCAAACGGCCAAGTATCATACGGAGCTCGCCCGACACAAAACAAGCCAAGCGAAACAAACGCATATAACGTAACAACACACGGAAACGGTCAAGTGTCATACGGCGCTCGTCCGACATACAACAAGCCAAGTAAAACAAATGCATACAATGTAACAACACATGCAGATGGTACTGCGACATATGGTCCTAGAGTAACAAAATAA

>ATCC_25923

ATGAAAAAGCAAATAATTTCGCTAGGCGCATTAGCAGTTGCATCTAGCTTATTTACATGGGATAACAAAGCAGATGCGATAGTAACTAAAGATTATAGTAAAGAATCAAGAGTGAATGAGAACAGTAAATACGATACACCAATTCCAGATTGGTATCTAGGTAGTATTTTAAACAGATTAGGGGATCAAATATACTACGCTAAGGAATTAACTAATAAATACGAATATGGTGAGAAAGAGTATAAGCAAGCGATAGATAAATTGATGACTAGAGTTTTGGGAGAAGATCATTATCTATTAGAAAAAAAGAAAGCACAATATGAAGCATACAAAAAATGGTTTGAAAAACATAAAAGTGAAAATCCACATTCTAGTTTAAAAAAGATTAAATTTGACGATTTTGATTTATATAGATTAACGAAGAAAGAATACAATGAGTTACATCAATCATTAAAAGAAGCTGTTGATGAGTTTAATAGTGAAGTGAAAAATATTCAATCTAAACAAAAGGATTTATTACCTTATGATGAAGCAACTGAAAATCGAGTAACAAATGGAATATATGATTTTGTTTGCGAGATTGACACATTATACGCAGCATATTTTAATCATAGCCAATATGGTCATAATGCTAAAGAATTAAGAGCAAAGCTAGATATAATTCTTGGTGATGCTAAAGATCCTGTTAGAATTACGAATGAAAGAATAAGAAAAGAAATGATGGATGATTTAAATTCTATTATTGATGATTTCTTTATGGATACAAACATGAATAGACCATTAAACATAACTAAATTTAATCCGAATATTCATGACTATACTAATAAGCCTGAAAATAGAGATAACTTCGATAAATTAGTCAAAGAAACAAGAGAAGCAGTCGCAAACGCTGACGAATCTTGGAAAACAAGAACCGTCAAAAATTACGGTGAATCTGAAACAAAATCTCCTGTTGTAAAAGAAGAGAAGAAAGTTGAAGAACCTCAATTACCTAAAGTTGGAAACCAGCAAGAGGATAAAATTACAGTTGGTACAACTGAAGAAGCACCATTACCAATTGCGCAACCACTAGTTAAAATTCCACAGGGCACAATTCAAGGTGAAATTGTAAAAGGTCCGGAATATCTAACGATGGAAAATAAAACGTTACAAGGTGAAATCGTTCAAGGTCCAGATTTCCCAACAATGGAACAAAACAGACCATCTTTAAGCGATAATTATACTCAACCGACGACACCGAACCCTATTTTAAAAGGTATTGAAGGAAACTCAACTAAACTTGAAATAAAACCACAAGGTACTGAATCAACGTTAAAAGGTACTCAAGGAGAATCAAGTGATATTGAAGTTAAACCTCAAGCAACTGAAACAACAGAAGCATCACATTATCCAGCGAGACCTCAATTTAACAAAACACCTAAGTATGTGAAATATAGAGATGCTGGTACAGGTATCCGTGAATACAACGATGGAACATTTGGATATGAAGCGAGACCAAGATTCAACAAGCCAAGCGAAACAAATGCATACAACGTAACGACAAATCAAGATGGCACAGTATCATATGGCGCTCGCCCGACACAAAACAAACCAAGCGAAACAAATGCATACAACGTAACAACACATGCAAACGGCCAAGTATCATATGGCGCCCGCCCAACATACAAGAAGCCAAGCGAAACAAACGCATACAACGTAACGACAAATCAAGATGGCACAGTATCATATGGCGCTCGCCCGACACAAAACAAGCCAAGCGAAACAAACGCATATAACGTAACAACACATGCAAACGGCCAAGTATCATACGGAGCTCGTCCGACACAAAACAAGCCAAGCGAAACGAACGCATATAACGTAACAACACATGCAAACGGTCAAGTGTCATACGGAGCTCGCCCAACACAAAACAAGCCAAGTAAAACAAATGCATACAATGTAACAACACATGCAGATGGTACTGCGACATATGGTCCTAGAGTAACAAAATAA

>NCTC 8325

ATGAAAAAGCAAATAATTTCGCTAGGCGCATTAGCAGTTGCATCTAGCTTATTTACATGGGATAACAAAGCAGATGCGATAGTAACAAAGGATTATAGTGGGAAATCACAAGTTAATGCTGGGAGTAAAAATGGGACATTAATAGATAGCAGATATTTAAATTCAGCTCTATATTATTTGGAAGACTATATAATTTATGCTATAGGATTAACTAATAAATATGAATATGGAGATAATATTTATAAAGAAGCTAAAGATAGGTTGTTGGAAAAGGTATTAAGGGAAGATCAATATCTTTTGGAGAGAAAGAAATCTCAATATGAAGATTATAAACAATGGTATGCAAATTATAAAAAAGAAAATCCTCGTACAGATTTAAAAATGGCTAATTTTCATAAATATAATTTAGAAGAACTTTCGATGAAAGAATACAATGAACTACAGGATGCATTAAAGAGAGCACTGGATGATTTTCACAGAGAAGTTAAAGATATTAAGGATAAGAATTCAGACTTGAAAACTTTTAATGCAGCAGAAGAAGATAAAGCAACTAAGGAAGTATACGATCTCGTATCTGAAATTGATACATTAGTTGTATCATATTATGGTGATAAGGATTATGGGGAGCACGCGAAAGAGTTACGAGCAAAACTGGACTTAATCCTTGGAGATACAGACAATCCACATAAAATTACAAATGAACGTATTAAAAAAGAAATGATTGATGACTTAAATTCAATTATTGATGATTTCTTTATGGAAACTAAACAAAATAGACCGAAATCTATAACGAAATATAATCCTACAACACATAACTATAAAACAAATAGTGATAATAAACCTAATTTTGATAAATTAGTTGAAGAAACGAAAAAAGCAGTTAAAGAAGCAGATGATTCTTGGAAAAAGAAAACTGTCAAAAAATACGGAGAAACTGAAACAAAATCGCCAGTAGTAAAAGAAGAGAAGAAAGTTGAAGAACCTCAAGCACCTAAAGTTGATAACCAACAAGAGGTTAAAACTACGGCTGGTAAAGCTGAAGAAACAACACAACCAGTTGCACAACCATTAGTTAAAATTCCACAGGGCACAATTACAGGTGAAATTGTAAAAGGTCCGGAATATCCAACGATGGAAAATAAAACGGTACAAGGTGAAATCGTTCAAGGTCCCGATTTTCTAACAATGGAACAAAGCGGCCCATCATTAAGCAATAATTATACAAACCCACCGTTAACGAACCCTATTTTAGAAGGTCTTGAAGGTAGCTCATCTAAACTTGAAATAAAACCACAAGGTACTGAATCAACGTTAAAAGGTACTCAAGGAGAATCAAGTGATATTGAAGTTAAACCTCAAGCAACTGAAACAACAGAAGCTTCTCAATATGGTCCGAGACCGCAATTTAACAAAACACCTAAATATGTTAAATATAGAGATGCTGGTACAGGTATCCGTGAATACAACGATGGAACATTTGGATATGAAGCGAGACCAAGATTCAATAAGCCATCAGAAACAAATGCATATAACGTAACAACACATGCAAATGGTCAAGTATCATACGGAGCTCGTCCGACATACAAGAAGCCAAGCGAAACGAATGCATACAATGTAACAACACATGCAAACGGCCAAGTATCATACGGAGCTCGTCCGACACAAAACAAGCCAAGCAAAACAAACGCATATAACGTAACAACACATGGAAACGGCCAAGTATCATATGGCGCTCGCCCAACACAAAACAAGCCAAGCAAAACAAATGCATACAACGTAACAACACATGCAAACGGTCAAGTGTCATACGGAGCTCGCCCGACATACAAGAAGCCAAGTAAAACAAATGCATACAATGTAACAACACATGCAGATGGTACTGCGACATATGGGCCTAGAGTAACAAAATAA

>Mu50

ATGAAAAAGCAAATAATTTCGCTAGGCGCATTAGCAGTTGCATCTAGCTTATTTACATGGGATAACAAAGCAGATGCGATAGTAACAAAGGATTATAGTAAAGAATCAAGAGTGAATGAGAAAAGTAAAAAGGGAGCTACTGTTTCAGATTATTACTATTGGAAAATAATTGATAGTTTAGAGGCACAATTTACTGGAGCAATAGACTTATTGGAAGATTATAAATATGGAGATCCTATCTATAAAGAAGCGAAAGATAGATTGATGACAAGAGTATTAGGAGAAGACCAGTATTTATTAAAGAAAAAGATTGATGAATATGAGCTTTATAAAAAGTGGTATAAAAGTTCAAATAAGAACACTAATATGCTTACTTTCCATAAATATAATCTTTACAATTTAACAATGAATGAATATAACGATATTTTTAACTCTTTGAAAGATGCAGTTTATCAATTTAATAAAGAAGTTAAAGAAATAGAGCATAAAAATGTTGACTTGAAGCAGTTTGATAAAGATGGAGAAGACAAGGCAACTAAAGAAGTTTATGACCTTGTTTCTGAAATTGATACATTAGTTGTAACTTATTATGCTGATAAGGATTATGGGGAGCATGCGAAAGAGTTACGAGCAAAACTGGACTTAATCCTTGGAGATACAGACAATCCACATAAAATTACAAATGAGCGTATAAAAAAAGAAATGATCGATGACTTAAATTCAATTATAGATGATTTCTTTATGGAGACTAAACAAAATAGACCGAATTCTATAACAAAATATGATCCAACAAAACACAATTTTAAAGAGAAGAGTGAAAATAAACCTAATTTTGATAAATTAGTTGAAGAAACAAAAAAAGCAGTTAAAGAAGCAGACGAATCTTGGAAAAATAAAACTGTCAAAAAATACGAGGAAACTGTAACAAAATCTCCTGTTGTAAAAGAAGAGAAGAAAGTTGAAGAACCTCAATTACCTAAAGTTGGAAACCAGCAAGAGGTTAAAACTACGGCTGGTAAAGCTGAAGAAACAACACAACCAGTGGCACAGCCATTAGTAAAAATTCCACAAGAAACAATCTATGGTGAAACTGTAAAAGGTCCAGAATATCCAACGATGGAAAATAAAACGTTACAAGGTGAAATCGTTCAAGGTCCCGATTTTCTAACAATGGAACAAAACAGACCATCTTTAAGCGATAATTATACTCAACCGACGACACCGAACCCTATTTTAGAAGGTCTTGAAGGTAGCTCATCTAAACTTGAAATAAAACCACAAGGTACTGAATCAACGTTGAAAGGTATTCAAGGAGAATCAAGTGATATTGAAGTTAAACCTCAAGCAACTGAAACAACAGAAGCTTCTCAATATGGTCCGAGACCGCAATTTAACAAAACACCTAAGTATGTGAAATATAGAGATGCTGGTACAGGTATCCGTGAATACAACGATGGAACATTTGGATATGAAGCGAGACCAAGATTCAACAAGCCAAGTGAAACAAATGCATACAACGTAACGACAAATCAAGATGGCACAGTATCATACGGAGCTCGCCCAACACAAAACAAGCCAAGTGAAACAAACGCATATAACGTAACAACACATGCAAATGGTCAAGTATCATACGGTGCTCGCCCAACACAAAAAAAGCCAAGCAAAACAAATGCATACAACGTAACAACACATGCAAATGGTCAAGTATCATATGGCGCTCGCCCGACACAAAAAAAGCCAAGCAAAACAAATGCATATAACGTAACAACACATGCAAATGGTCAAGTATCATACGGAGCTCGCCCGACATACAAGAAGCCAAGCGAAACAAATGCATACAACGTAACAACACATGCAAATGGTCAAGTATCATATGGCGCTCGCCCGACACAAAAAAAGCCAAGCGAAACAAACGCATATAACGTAACAACACATGCAGATGGTACTGCGACATATGGGCCTAGAGTAACAAAATAA

>ATCC_6538

ATGAAAAAGCAAATAATTTCGCTAGGCGCATTAGCAGTTGCATCTAGCTTATTTACATGGGATAACAAAGCAGATGCGATAGTAACAAAGGATTATAATGGGAAATCACAAGTTAATGCTGGGAGTAAAAATGGGACACCGATTTCTAATGGTTACTTTTGGGGGAAAATCGATAGTCTAGAATCACAATTTTCTAAAGCATTAGCAATAATTGAAGAGTATCAATATGGTGAGAAAGAATATAAAGATGCAAAAGATAAGTTTATGGATAGAATTTTGTCAGAAGATCAATATCTTTTGGAGAAAAAGAAAGCTCTGTATGAGAAATATAAAGAGTGGTATAAAAAGCACAAAGAGATAAACCCAACTTATCCAAAAATGCAAACATTCCATGAATTTAGTGTATATAATCTAACTATGGAAGAATACAATGAGATTTCTAAATCTTTGAAAGATGCGGAAGAAGAGTTTCGTAAAAATGTAAGCGAAGTTCAACTACAAAATTCTGATTTGAAGTCTTTTGACAAAACTAGAGAAACTAAGGCTACAGATGATATATACGATTTTGTTTGTGAAATAGACACACTTGTAGCTACGTATTATGGCGACCAGAATTATAGAGAAAATGCGAAAGAACTTAGAATGAAAATGGATTTAATACTTGGAGATTCTGATAACCCTAACCGAATTACGAATGAAAGAATTAAAAATGAAATGATGAAAGATTTAAATTCAATCATTGATGATTTCTTTATAGATACTAATCAAAACAGACCTACAACGATAAAGAAATACGATCCTAGAATTCATGACTTTACTAAGAAAAAAGAGAATAAAAGTAACTTCGATGCTTTAGTTAAAGAAACTAAAGATGCGGTTGCTAAGGCTGATAATTCTTGGAAAACTAAAACTGTCAAAACATATGGTGAAGCTGAAACAAAAGCACATGTTGTAAAAGAAGAGAAGAAAGTTGAAGAACCTCAATTACCTAAAGTTGGAAACCAGCAAGAGGATAAAACTACAGTTGATAAAGCTGAAGAAACAACACAACCAGTGGCACAGCCATTAGTTAAAATTCCACAGGGCACAATTACAGGTGAAATTGTAAAAGGTCCAGACTATCCAACTATGGAAAATAAAACGTTACAAGGTGAAATCGTTCAAGGTCCAGATTTCCCAACAATGGAACAAAGCGGCCCATCATTAAGCAATAATTATACAAACCCACCGTTAACGGACCCTATTTTAGAAGGTCTTGAAGGTAGCTCATCTAAACTTGAAATAAAACCACAAGGTACTGAATCAACGTTGAAAGGTACTCAAGGAGAATCAAGTGATATTGAAGTTAAACCTCAAGCAACTGAAACAACAGAAGCTTCAAAATATGGTCCGAGACCGCAATTTAACAAAACACCTAAGTATGTGAAATATAGAGATGCTGGTACAGGTATCCGTGAATACAACGATGGAACATTTGGATATGAAGCGAGACCAAGATTCAACAAGCCAAGTGAAACAAATGCATACAACGTAACGACAAATCAAGATGGCACAGTAACATATGGCGCTCGCCCAACACAAAATAAGCCATCAGAAACAAATGCATATAACGTAACAACACATGCAAACGGCCAAGTGTCATACGGTGCTCGTCCGACATACAAGAAACCAAGTAAAACAAACGCATACAACGTAACGACAAATCAAGATGGCACAGTATCATATGGGGCTCGCCCAACACAAAACAAGCCAAGCAAAACAAATGCATATAACGTAACAACACATGCAAACGGCCAAGTATCATACGGAGCTCGCCCGACATACAAGAAACCAAGCAAAACAAATGCATACAACGTAACAACACATGCAAATGGTCAAGTATCATATGGCGCTCGCCCGACATACAAGAAACCAAGCAAAACAAATGCATATAACGTAACAACACATGCAAATGGTCAAGTATCATACGGAGCTCGCCCGACATACAAGAAGCCAAGCGAAACAAATGCATACAACGTAACAACACATGCAAATGGTCAAGTATCATATGGCGCTCGCCCGACACAAAAAAAGCCAAGCGAAACAAACGCATATAACGTAACAACACATGCAGATGGTACTGCGACATATGGGCCTAGAGTAACAAAATAA

>ATCC_29213

ATGAAAAAGCAAATAATTTCGCTAGGCGCATTAGCAGTTGCATCTAGCTTATTTACATGGGATAACAAAGCAGATGCGATAGTAACAAAGGATTATAGTAAAGAATCAAGAGTGAATGAGAAAAGTAAAAAGGGAGCTACTGTTTCAGATTATTACTATTGGAAAATAATTGATAGTTTAGAGGCACAATTTACTGGAGCAATAGACTTATTGGAAGATTATAAATATGGAGATCCTATCTATAAAGAAGCGAAAGATAGATTGATGACAAGAGTATTAGGAGAAGACCAGTATTTATTAAAGAAAAAGATTGATGAATATGAGCTTTATAAAAAGTGGTATAAAAGTTCAAATAAGAACACTAATATGCTTACTTTCCATAAATATAATCTTTACAATTTAACAATGAATGAATATAACGATATTTTTAACTCTTTGAAAGATGCAGTTTATCAATTTAATAAAGAAGTTAAAGAAATAGAGCATAAAAATGTTGACTTGAAGCAGTTTGATAAAGATGGAGAAGACAAGGCAACTAAAGAAGTTTATGACCTTGTTTCTGAAATTGATACATTAGTTGTAACTTATTATGCTGATAAGGATTATGGGGAGCATGCTAAAGAGTTACGAGCAAAACTGGACTTAATCCTTGGAGATACAGACAATCCACATAAAATTACAAATGAGCGTATAAAAAAAGAAATGATCGATGACTTAAATTCAATTATAGATGATTTCTTTATGGAGACTAAACAAAATAGACCGAATTCTATAACAAAATATGATCCAACAAAACACAATTTTAAAGAGAAGAGTGAAAATAAACCTAATTTTGATAAATTAGTTGAAGAAACAAAAAAAGCAGTTAAAGAAGCAGACGAATCTTGGAAAAATAAAACTGTCAAAAAATACGAGGAAACTGTAACAAAATCTCCTGTTGTAAAAGAAGAGAAGAAAGTTGAAGAACCTCAATTACCTAAAGTTGGAAACCAGCAAGAGGTTAAAACTACGGCTGGTAAAGCTGAAGAAACAACACAACCAGTGGCACAGCCATTAGTAAAAATTCCACAAGAAACAATCTATGGTGAAACTGTAAAAGGTCCAGAATATCCAACGATGGAAAATAAAACGTTACAAGGTGAAATCGTTCAAGGTCCCGATTTTCTAACAATGGAACAAAACAGACCATCTTTAAGCGATAATTATACTCAACCGACGACACCGAACCCTATTTTAGAAGGTCTTGAAGGTAGCTCATCTAAACTTGAAATAAAACCACAAGGTACTGAATCAACGTTGAAAGGTATTCAAGGAGAATCAAGTGATATTGAAGTTAAACCTCAAGCAACTGAAACAACAGAAGCTTCTCAATATGGTCCGAGACCGCAATTTAACAAAACACCTAAGTATGTGAAATATAGAGATGCTGGTACAGGTATCCGTGAATACAACGATGGAACATTTGGATATGAAGCGAGACCAAGATTCAACAAGCCAAGTGAAACAAATGCATACAACGTAACGACAAATCAAGATGGCACAGTATCATACGGAGCTCGCCCAACACAAAACAAGCCAAGTGAAACAAACGCATATAACGTAACAACACATGCAAATGGTCAAGTATCATACGGTGCTCGCCCAACACAAAACAAGCCAAGCAAAACAAATGCATACAACGTAACAACACATGCAAATGGTCAAGTATCATATGGCGCTCGCCCGACACAAAAAAAGCCAAGCAAAACAAATGCATATAACGTAACAACACATGCAAATGGTCAAGTATCATACGGAGCTCGCCCGACATACAAGAAGCCAAGCGAAACAAATGCATACAACGTAACAACACATGCAAATGGTCAAGTATCATATGGCGCTCGCCCGACACAAAAAAAGCCAAGCGAAACAAACGCATATAACGTAACAACACATGCAGATGGTACTGCGACATATGGGCCTAGAGTAACAAAATAA

>USA300_SUR1

ATGAAAAAGCAAATAATTTCGCTAGGCGCATTAGCAGTTGCATCTAGCTTATTTACATGGGATAACAAAGCAGATGCGATAGTAACAAAGGATTATAGTGGGAAATCACAAGTTAATGCTGGGAGTAAAAATGGGACATTAATAGATAGCAGATATTTAAATTCAGCTCTATATTATTTGGAAGACTATATAATTTATGCTATAGGATTAACTAATAAATATGAATATGGAGATAATATTTATAAAGAAGCTAAAGATAGGTTGTTGGAAAAGGTATTAAGGGAAGATCAATATCTTTTGGAGAGAAAGAAATCTCAATATGAAGATTATAAACAATGGTATGCAAATTATAAAAAAGAAAATCCTCGTACAGATTTAAAAATGGCTAATTTTCATAAATATAATTTAGAAGAACTTTCGATGAAAGAATACAATGAACTACAGGATGCATTAAAGAGAGCACTGGATGATTTTCACAGAGAAGTTAAAGATATTAAGGATAAGAATTCAGACTTGAAAACTTTTAATGCAGCAGAAGAAGATAAAGCAACTAAGGAAGTATACGATCTCGTATCTGAAATTGATACATTAGTTGTATCATATTATGGTGATAAGGATTATGGGGAGCACGCGAAAGAGTTACGAGCAAAACTGGACTTAATCCTTGGAGATACAGACAATCCACATAAAATTACAAATGAACGTATTAAAAAAGAAATGATTGATGACTTAAATTCAATTATTGATGATTTCTTTATGGAAACTAAACAAAATAGACCGAAATCTATAACGAAATATAATCCTACAACACATAACTATAAAACAAATAGTGATAATAAACCTAATTTTGATAAATTAGTTGAAGAAACGAAAAAAGCAGTTAAAGAAGCAGATGATTCTTGGAAAAAGAAAACTGTCAAAAAATACGGAGAAACTGAAACAAAATCGCCAGTAGTAAAAGAAGAGAAGAAAGTTGAAGAACCTCAAGCACCTAAAGTTGATAACCAACAAGAGGTTAAAACTACGGCTGGTAAAGCTGAAGAAACAACACAACCAGTTGCACAACCATTAGTTAAAATTCCACAGGGCACAATTACAGGTGAAATTGTAAAAGGTCCGGAATATCCAACGATGGAAAATAAAACGGTACAAGGTGAAATCGTTCAAGGTCCCGATTTTCTAACAATGGAACAAAGCGGCCCATCATTAAGCAATAATTATACAAACCCACCGTTAACGAACCCTATTTTAGAAGGTCTTGAAGGTAGCTCATCTAAACTTGAAATAAAACCACAAGGTACTGAATCAACGTTAAAAGGTACTCAAGGAGAATCAAGTGATATTGAAGTTAAACCTCAAGCAACTGAAACAACAGAAGCTTCTCAATATGGTCCGAGACCGCAATTTAACAAAACACCTAAATATGTTAAATATAGAGATGCTGGTACAGGTATCCGTGAATACAACGATGGAACATTTGGATATGAAGCGAGACCAAGATTCAATAAGCCATCAGAAACAAATGCATATAACGTAACAACACATGCAAATGGTCAAGTATCATACGGAGCTCGTCCGACACAAAACAAGCCAAGCAAAACAAACGCATATAACGTAACAACACATGGAAACGGCCAAGTATCATATGGCGCTCGCCCAACACAAAACAAGCCAAGCAAAACAAATGCATACAACGTAACAACACATGCAAACGGTCAAGTGTCATACGGAGCTCGCCCGACATACAAGAAGCCAAGTAAAACAAATGCATACAATGTAACAACACATGCAGATGGTACTGCGACATATGGGCCTAGAGTAACAAAATAA

>NCTC_08532_e2_c73

ATGAAAAAGCAAATAATTTCGCTAGGCGCATTAGCAGTTGCATCTAGCTTATTTACATGGGATAACAAAGCAGATGCGATAGTAACAAAGGATTATAGTGGGAAATCACAAGTTAATGCTGGGAGTAAAAATGGGACATTAATAGATAGCAGATATTTAAATTCAGCTCTATATTATTTGGAAGACTATATAATTTATGCTATAGGATTAACTAATAAATATGAATATGGAGATAATATTTATAAAGAAGCTAAAGATAGGTTGTTGGAAAAGGTATTAAGGGAAGATCAATATCTTTTGGAGAGAAAGAAATCTCAATATGAAGATTATAAACAATGGTATGCAAATTATAAAAAAGAAAATCCTCGTACAGATTTAAAAATGGCTAATTTTCATAAATATAATTTAGAAGAACTTTCGATGAAAGAATACAATGAACTACAGGATGCATTAAAGAGAGCACTGGATGATTTTCACAGAGAAGTTAAAGATATTAAGGATAAGAATTCAGACTTGAAAACTTTTAATGCAGCAGAAGAAGATAAAGCAACTAAGGAAGTATACGATCTCGTATCTGAAATTGATACATTAGTTGTATCATATTATGGTGATAAGGATTATGGGGAGCACGCGAAAGAGTTACGAGCAAAACTGGACTTAATCCTTGGAGATACAGACAATCCACATAAAATTACAAATGAACGTATTAAAAAAGAAATGATTGATGACTTAAATTCAATTATTGATGATTTCTTTATGGAAACTAAACAAAATAGACCGAAATCTATAACGAAATATAATCCTACAACACATAACTATAAAACAAATAGTGATAATAAACCTAATTTTGATAAATTAGTTGAAGAAACGAAAAAAGCAGTTAAAGAAGCAGATGATTCTTGGAAAAAGAAAACTGTCAAAAAATACGGAGAAACTGAAACAAAATCGCCAGTAGTAAAAGAAGAGAAGAAAGTTGAAGAACCTCAAGCACCTAAAGTTGATAACCAACAAGAGGTTAAAACTACGGCTGGTAAAGCTGAAGAAACAACACAACCAGTTGCACAACCATTAGTTAAAATTCCACAGGGCACAATTACAGGTGAAATTGTAAAAGGTCCGGAATATCCAACGATGGAAAATAAAACGGTACAAGGTGAAATCGTTCAAGGTCCCGATTTTCTAACAATGGAACAAAGCGGCCCATCATTAAGCAATAATTATACAAACCCACCGTTAACGAACCCTATTTTAGAAGGTCTTGAAGGTAGCTCATCTAAACTTGAAATAAAACCACAAGGTACTGAATCAACGTTAAAAGGTACTCAAGGAGAATCAAGTGATATTGAAGTTAAACCTCAAGCAACTGAAACAACAGAAGCTTCTCAATATGGTCCGAGACCGCAATTTAACAAAACACCTAAATATGTTAAATATAGAGATGCTGGTACAGGTATCCGTGAATACAACGATGGAACATTTGGATATGAAGCGAGACCAAGATTCAATAAGCCATCAGAAACAAATGCATATAACGTAACAACACATGCAAATGGTCAAGTATCATACGGAGCTCGTCCGACATACAAGAAGCCAAGCGAAACGAATGCATACAATGTAACAACACATGCAAACGGCCAAGTATCATACGGAGCTCGTCCGACACAAAACAAGCCAAGCAAAACAAACGCATATAACGTAACAACACATGGAAACGGCCAAGTATCATATGGCGCTCGCCCAACACAAAACAAGCCAAGCAAAACAAATGCATACAACGTAACAACACATGCAAACGGTCAAGTGTCATACGGAGCTCGCCCGACATACAAGAAGCCAAGTAAAACAAATGCATACAATGTAACAACACATGCAGATGGTACTGCGACATATGGGCCTAGAGTAACAAAATAA

>MRSA252

ATGAAAAAGCAAATAATTTCGCTAGGCGCATTAGCAGTTGCATCTAGCTTATTTACATGGGATAACAAAGCAGATGCGATAGTAACTAAAGATTATAGTAAAGAATCAAGAGTGAATGAGAACAGTAAATACGATACACCAATTCCAGATTGGTATCTAGGTAGTATTTTAAACAGATTAGGGGATCAAATATACTACGCTAAGGAATTAACTAATAAATACGAATATGGTGAGAAAGAGTATAAGCAAGCGATAGATAAATTGATGACTAGAGTTTTGGGAGAAGATCATTATCTATTAGAAAAAAAGAAAGCACAATATGAAGCATACAAAAAATGGTTTGAAAAACATAAAAGTGAAAATCCACATTCTAGTTTAAAAAAGATTAAATTTGACGATTTTGATTTATATAGATTAACGAAGAAAGAATACAATGAGTTACATCAATCATTAAAAGAAGCTGTTGATGAGTTTAATAGTGAAGTGAAAAATATTCAATCTAAACAAAAGGATTTATTACCTTATGATGAAGCAACTGAAAATCGAGTAACAAATGGAATATATGATTTTGTTTGCGAGATTGACACATTATACGCAGCATATTTTAATCATAGCCAATATGGTCATAATGCTAAAGAATTAAGAGCAAAGCTAGATATAATTCTTGGTGATGCTAAAGATCCTGTTAGAATTACGAATGAAAGAATAAGAAAAGAAATGATGGATGATTTAAATTCTATTATTGATGATTTCTTTATGGATACAAACATGAATAGACCATTAAACATAACTAAATTTAATCCGAATATTCATGACTATACTAATAAGCCTGAAAATAGAGATAACTTCGATAAATTAGTCAAAGAAACAAGAGAAGCAATCGCAAACGCTGACGAATCTTGGAAAACAAGAACCGTCAAAAATTACGGTGAATCTGAAACAAAATCTCCTGTTGTAAAAGAAGAGAAGAAAGTTGAAGAACCTCAATTACCTAAAGTTGGAAACCAGCAAGAGGATAAAATTACAGTTGGTACAACTGAAGAAGCACCATTACCAATTGCGCAACCACTAGTTAAAATTCCACAGGGCACAATTCAAGGTGAAATTGTAAAAGGTCCGGAATATCTAACGATGGAAAATAAAACGTTACAAGGTGAAATCGTTCAAGGTCCAGATTTCCCAACAATGGAACAAAACAGACCATCTTTAAGCGATAATTATACTCAACCGACGACACCGAACCCTATTTTAAAAGGTATTGAAGGAAACTCAACTAAACTTGAAATAAAACCACAAGGTACTGAATCAACGTTAAAAGGTACTCAAGGAGAATCAAGTGATATTGAAGTTAAACCTCAAGCAACTGAAACAACAGAAGCATCACATTATCCAGCGAGACCTCAATTTAACAAAACACCTAAGTATGTGAAATATAGAGATGCTGGTACAGGTATCCGTGAATACAACGATGGAACATTTGGATATGAAGCGAGACCAAGATTCAACAAGCCAAGCGAAACAAATGCATACAACGTAACGACAAATCAAGATGGCACAGTATCATATGGCGCTCGCCCGACACAAAACAAGCCAAGCGAAACAAACGCATATAACGTAACAACACATGCAAACGGCCAAGTATCATACGGAGCTCGTCCGACACAAAACAAGCCAAGCGAAACGAACGCATATAACGTAACAACACATGCAAACGGTCAAGTGTCATACGGAGCTCGCCCAACACAAAACAAGCCAAGTAAAACAAATGCATACAATGTAACAACACATGCAGATGGTACTGCGACATATGGTCCTAGAGTAACAAAATAA

>Newman

ATGAAAAAGCAAATAATTTCGCTAGGCGCATTAGCAGTTGCATCTAGCTTATTTACATGGGATAACAAAGCAGATGCGATAGTAACAAAGGATTATAGTGGGAAATCACAAGTTAATGCTGGGAGTAAAAATGGGACATTAATAGATAGCAGATATTTAAATTCAGCTCTATATTATTTGGAAGACTATATAATTTATGCTATAGGATTAACTAATAAATATGAATATGGAGATAATATTTATAAAGAAGCTAAAGATAGGTTGTTGGAAAAGGTATTAAGGGAAGATCAATATCTTTTGGAGAGAAAGAAATCTCAATATGAAGATTATAAACAATGGTATGCAAATTATAAAAAAGAAAATCCTCGTACAGATTTAAAAATGGCTAATTTTCATAAATATAATTTAGAAGAACTTTCGATGAAAGAATACAATGAACTACAGGATGCATTAAAGAGAGCACTGGATGATTTTCACAGAGAAGTTAAAGATATTAAGGATAAGAATTCAGACTTGAAAACTTTTAATGCAGCAGAAGAAGATAAAGCAACTAAGGAAGTATACGATCTCGTATCTGAAATTGATACATTAGTTGTATCATATTATGGTGATAAGGATTATGGGGAGCACGCGAAAGAGTTACGAGCAAAACTGGACTTAATCCTTGGAGATACAGACAATCCACATAAAATTACAAATGAACGTATTAAAAAAGAAATGATTGATGACTTAAATTCAATTATTGATGATTTCTTTATGGAAACTAAACAAAATAGACCGAAATCTATAACGAAATATAATCCTACAACACATAACTATAAAACAAATAGTGATAATAAACCTAATTTTGATAAATTAGTTGAAGAAACGAAAAAAGCAGTTAAAGAAGCAGATGATTCTTGGAAAAAGAAAACTGTCAAAAAATACGGAGAAACTGAAACAAAATCGCCAGTAGTAAAAGAAGAGAAGAAAGTTGAAGAACCTCAAGCACCTAAAGTTGATAACCAACAAGAGGTTAAAACTACGGCTGGTAAAGCTGAAGAAACAACACAACCAGTTGCACAACCATTAGTTAAAATTCCACAGGGCACAATTACAGGTGAAATTGTAAAAGGTCCGGAATATCCAACGATGGAAAATAAAACGGTACAAGGTGAAATCGTTCAAGGTCCCGATTTTCTAACAATGGAACAAAGCGGCCCATCATTAAGCAATAATTATACAAACCCACCGTTAACGAACCCTATTTTAGAAGGTCTTGAAGGTAGCTCATCTAAACTTGAAATAAAACCACAAGGTACTGAATCAACGTTAAAAGGTACTCAAGGAGAATCAAGTGATATTGAAGTTAAACCTCAAGCAACTGAAACAACAGAAGCTTCTCAATATGGTCCGAGACCGCAATTTAACAAAACACCTAAATATGTTAAATATAGAGATGCTGGTACAGGTATCCGTGAATACAACGATGGAACATTTGGATATGAAGCGAGACCAAGATTCAATAAGCCATCAGAAACAAATGCATATAACGTAACAACACATGCAAATGGTCAAGTATCATACGGAGCTCGTCCGACATACAAGAAGCCAAGCGAAACGAATGCATACAATGTAACAACACATGCAAACGGCCAAGTATCATACGGAGCTCGTCCGACACAAAACAAGCCAAGCAAAACAAACGCATATAACGTAACAACACATGGAAACGGCCAAGTATCATATGGCGCTCGCCCAACACAAAACAAGCCAAGCAAAACAAATGCATACAACGTAACAACACATGCAAACGGTCAAGTGTCATACGGAGCTCGCCCGACATACAAGAAGCCAAGTAAAACAAATGCATACAATGTAACAACACATGCAGATGGTACTGCGACATATGGGCCTAGAGTAACAAAATAA

>Newman_D2C

ATGAAAAAGCAAATAATTTCGCTAGGCGCATTAGCAGTTGCATCTAGCTTATTTACATGGGATAACAAAGCAGATGCGATAGTAACAAAGGATTATAGTGGGAAATCACAAGTTAATGCTGGGAGTAAAAATGGGACATTAATAGATAGCAGATATTTAAATTCAGCTCTATATTATTTGGAAGACTATATAATTTATGCTATAGGATTAACTAATAAATATGAATATGGAGATAATATTTATAAAGAAGCTAAAGATAGGTTGTTGGAAAAGGTATTAAGGGAAGATCAATATCTTTTGGAGAGAAAGAAATCTCAATATGAAGATTATAAACAATGGTATGCAAATTATAAAAAAGAAAATCCTCGTACAGATTTAAAAATGGCTAATTTTCATAAATATAATTTAGAAGAACTTTCGATGAAAGAATACAATGAACTACAGGATGCATTAAAGAGAGCACTGGATGATTTTCACAGAGAAGTTAAAGATATTAAGGATAAGAATTCAGACTTGAAAACTTTTAATGCAGCAGAAGAAGATAAAGCAACTAAGGAAGTATACGATCTCGTATCTGAAATTGATACATTAGTTGTATCATATTATGGTGATAAGGATTATGGGGAGCACGCGAAAGAGTTACGAGCAAAACTGGACTTAATCCTTGGAGATACAGACAATCCACATAAAATTACAAATGAACGTATTAAAAAAGAAATGATTGATGACTTAAATTCAATTATTGATGATTTCTTTATGGAAACTAAACAAAATAGACCGAAATCTATAACGAAATATAATCCTACAACACATAACTATAAAACAAATAGTGATAATAAACCTAATTTTGATAAATTAGTTGAAGAAACGAAAAAAGCAGTTAAAGAAGCAGATGATTCTTGGAAAAAGAAAACTGTCAAAAAATACGGAGAAACTGAAACAAAATCGCCAGTAGTAAAAGAAGAGAAGAAAGTTGAAGAACCTCAAGCACCTAAAGTTGATAACCAACAAGAGGTTAAAACTACGGCTGGTAAAGCTGAAGAAACAACACAACCAGTTGCACAACCATTAGTTAAAATTCCACAGGGCACAATTACAGGTGAAATTGTAAAAGGTCCGGAATATCCAACGATGGAAAATAAAACGGTACAAGGTGAAATCGTTCAAGGTCCCGATTTTCTAACAATGGAACAAAGCGGCCCATCATTAAGCAATAATTATACAAACCCACCGTTAACGAACCCTATTTTAGAAGGTCTTGAAGGTAGCTCATCTAAACTTGAAATAAAACCACAAGGTACTGAATCAACGTTAAAAGGTACTCAAGGAGAATCAAGTGATATTGAAGTTAAACCTCAAGCAACTGAAACAACAGAAGCTTCTCAATATGGTCCGAGACCGCAATTTAACAAAACACCTAAATATGTTAAATATAGAGATGCTGGTACAGGTATCCGTGAATACAACGATGGAACATTTGGATATGAAGCGAGACCAAGATTCAATAAGCCATCAGAAACAAATGCATATAACGTAACAACACATGCAAATGGTCAAGTATCATACGGAGCTCGTCCGACATACAAGAAGCCAAGCGAAACGAATGCATACAATGTAACAACACATGCAAACGGCCAAGTATCATACGGAGCTCGTCCGACACAAAACAAGCCAAGCAAAACAAACGCATATAACGTAACAACACATGGAAACGGCCAAGTATCATATGGCGCTCGCCCAACACAAAACAAGCCAAGCAAAACAAATGCATACAACGTAACAACACATGCAAACGGTCAAGTGTCATACGGAGCTCGCCCGACATACAAGAAGCCAAGTAAAACAAATGCATACAATGTAACAACACATGCAGATGGTACTGCGACATATGGGCCTAGAGTAACAAAATAA

>N315

ATGAAAAAGCAAATAATTTCGCTAGGCGCATTAGCAGTTGCATCTAGCTTATTTACATGGGATAACAAAGCAGATGCGATAGTAACAAAGGATTATAGTAAAGAATCAAGAGTGAATGAGAAAAGTAAAAAGGGAGCTACTGTTTCAGATTATTACTATTGGAAAATAATTGATAGTTTAGAGGCACAATTTACTGGAGCAATAGACTTATTGGAAGATTATAAATATGGAGATCCTATCTATAAAGAAGCGAAAGATAGATTGATGACAAGAGTATTAGGAGAAGACCAGTATTTATTAAAGAAAAAGATTGATGAATATGAGCTTTATAAAAAGTGGTATAAAAGTTCAAATAAGAACACTAATATGCTTACTTTCCATAAATATAATCTTTACAATTTAACAATGAATGAATATAACGATATTTTTAACTCTTTGAAAGATGCAGTTTATCAATTTAATAAAGAAGTTAAAGAAATAGAGCATAAAAATGTTGACTTGAAGCAGTTTGATAAAGATGGAGAAGACAAGGCAACTAAAGAAGTTTATGACCTTGTTTCTGAAATTGATACATTAGTTGTAACTTATTATGCTGATAAGGATTATGGGGAGCATGCGAAAGAGTTACGAGCAAAACTGGACTTAATCCTTGGAGATACAGACAATCCACATAAAATTACAAATGAGCGTATAAAAAAAGAAATGATCGATGACTTAAATTCAATTATAGATGATTTCTTTATGGAGACTAAACAAAATAGACCGAATTCTATAACAAAATATGATCCAACAAAACACAATTTTAAAGAGAAGAGTGAAAATAAACCTAATTTTGATAAATTAGTTGAAGAAACAAAAAAAGCAGTTAAAGAAGCAGACGAATCTTGGAAAAATAAAACTGTCAAAAAATACGAGGAAACTGTAACAAAATCTCCTGTTGTAAAAGAAGAGAAGAAAGTTGAAGAACCTCAATTACCTAAAGTTGGAAACCAGCAAGAGGTTAAAACTACGGCTGGTAAAGCTGAAGAAACAACACAACCAGTGGCACAGCCATTAGTAAAAATTCCACAAGAAACAATCTATGGTGAAACTGTAAAAGGTCCAGAATATCCAACGATGGAAAATAAAACGTTACAAGGTGAAATCGTTCAAGGTCCCGATTTTCTAACAATGGAACAAAACAGACCATCTTTAAGCGATAATTATACTCAACCGACGACACCGAACCCTATTTTAGAAGGTCTTGAAGGTAGCTCATCTAAACTTGAAATAAAACCACAAGGTACTGAATCAACGTTGAAAGGTATTCAAGGAGAATCAAGTGATATTGAAGTTAAACCTCAAGCAACTGAAACAACAGAAGCTTCTCAATATGGTCCGAGACCGCAATTTAACAAAACACCTAAGTATGTGAAATATAGAGATGCTGGTACAGGTATCCGTGAATACAACGATGGAACATTTGGATATGAAGCGAGACCAAGATTCAACAAGCCAAGTGAAACAAATGCATACAACGTAACGACAAATCAAGATGGCACAGTATCATACGGAGCTCGCCCAACACAAAACAAGCCAAGTGAAACAAACGCATATAACGTAACAACACATGCAAATGGTCAAGTATCATACGGTGCTCGCCCAACACAAAAAAAGCCAAGCAAAACAAATGCATACAACGTAACAACACATGCAAATGGTCAAGTATCATATGGCGCTCGCCCGACACAAAAAAAGCCAAGCAAAACAAATGCATATAACGTAACAACACATGCAAATGGTCAAGTATCATACGGAGCTCGCCCGACATACAAGAAGCCAAGCGAAACAAATGCATACAACGTAACAACACATGCAAATGGTCAAGTATCATATGGCGCTCGCCCGACACAAAAAAAGCCAAGCGAAACAAACGCATATAACGTAACAACACATGCAGATGGTACTGCGACATATGGGCCTAGAGTAACAAAATAA

>JH9

ATGAAAAAGCAAATAATTTCGCTAGGCGCATTAGCAGTTGCATCTAGCTTATTTACATGGGATAACAAAGCAGATGCGATAGTAACAAAGGATTATAGTAAAGAATCAAGAGTGAATGAGAAAAGTAAAAAGGGAGCTACTGTTTCAGATTATTACTATTGGAAAATAATTGATAGTTTAGAGGCACAATTTACTGGAGCAATAGACTTATTGGAAGATTATAAATATGGAGATCCTATCTATAAAGAAGCGAAAGATAGATTGATGACAAGAGTATTAGGAGAAGACCAGTATTTATTAAAGAAAAAGATTGATGAATATGAGCTTTATAAAAAGTGGTATAAAAGTTCAAATAAGAACACTAATATGCTTACTTTCCATAAATATAATCTTTACAATTTAACAATGAATGAATATAACGATATTTTTAACTCTTTGAAAGATGCAGTTTATCAATTTAATAAAGAAGTTAAAGAAATAGAGCATAAAAATGTTGACTTGAAGCAGTTTGATAAAGATGGAGAAGACAAGGCAACTAAAGAAGTTTATGACCTTGTTTCTGAAATTGATACATTAGTTGTAACTTATTATGCTGATAAGGATTATGGGGAGCATGCGAAAGAGTTACGAGCAAAACTGGACTTAATCCTTGGAGATACAGACAATCCACATAAAATTACAAATGAGCGTATAAAAAAAGAAATGATCGATGACTTAAATTCAATTATAGATGATTTCTTTATGGAGACTAAACAAAATAGACCGAATTCTATAACAAAATATGATCCAACAAAACACAATTTTAAAGAGAAGAGTGAAAATAAACCTAATTTTGATAAATTAGTTGAAGAAACAAAAAAAGCAGTTAAAGAAGCAGACGAATCTTGGAAAAATAAAACTGTCAAAAAATACGAGGAAACTGTAACAAAATCTCCTGTTGTAAAAGAAGAGAAGAAAGTTGAAGAACCTCAATTACCTAAAGTTGGAAACCAGCAAGAGGTTAAAACTACGGCTGGTAAAGCTGAAGAAACAACACAACCAGTGGCACAGCCATTAGTAAAAATTCCACAAGAAACAATCTATGGTGAAACTGTAAAAGGTCCAGAATATCCAACGATGGAAAATAAAACGTTACAAGGTGAAATCGTTCAAGGTCCCGATTTTCTAACAATGGAACAAAACAGACCATCTTTAAGCGATAATTATACTCAACCGACGACACCGAACCCTATTTTAGAAGGTCTTGAAGGTAGCTCATCTAAACTTGAAATAAAACCACAAGGTACTGAATCAACGTTGAAAGGTATTCAAGGAGAATCAAGTGATATTGAAGTTAAACCTCAAGCAACTGAAACAACAGAAGCTTCTCAATATGGTCCGAGACCGCAATTTAACAAAACACCTAAGTATGTGAAATATAGAGATGCTGGTACAGGTATCCGTGAATACAACGATGGAACATTTGGATATGAAGCGAGACCAAGATTCAACAAGCCAAGTGAAACAAATGCATACAACGTAACGACAAATCAAGATGGCACAGTATCATACGGAGCTCGCCCAACACAAAACAAGCCAAGTGAAACAAACGCATATAACGTAACAACACATGCAAATGGTCAAGTATCATACGGTGCTCGCCCAACACAAAAAAAGCCAAGCAAAACAAATGCATACAACGTAACAACACATGCAAATGGTCAAGTATCATATGGCGCTCGCCCGACACAAAAAAAGCCAAGCAAAACAAATGCATATAACGTAACAACACATGCAAATGGTCAAGTATCATACGGAGCTCGCCCGACATACAAGAAGCCAAGCGAAACAAATGCATACAACGTAACAACACATGCAAATGGTCAAGTATCATATGGCGCTCGCCTGACACAAAAAAAGCCAAGCGAAACAAACGCATATAACGTAACAACACATGCAGATGGTACTGCGACATATGGGCCTAGAGTAACAAAATAA

>aureus_COL

ATGAAAAAGCAAATAATTTCGCTAGGCGCATTAGCAGTTGCATCTAGCTTATTTACATGGGATAACAAAGCAGATGCGATAGTAACAAAGGATTATAGTGGGAAATCACAAGTTAATGCTGGGAGTAAAAATGGGACATTAATAGATAGCAGATATTTAAATTCAGCTCTATATTATTTGGAAGACTATATAATTTATGCTATAGGATTAACTAATAAATATGAATATGGAGATAATATTTATAAAGAAGCTAAAGATAGGTTGTTGGAAAAGGTATTAAGGGAAGATCAATATCTTTTGGAGAGAAAGAAATCTCAATATGAAGATTATAAACAATGGTATGCAAATTATAAAAAAGAAAATCCTCGTACAGATTTAAAAATGGCTAATTTTCATAAATATAATTTAGAAGAACTTTCGATGAAAGAATACAATGAACTACAGGATGCATTAAAGAGAGCACTGGATGATTTTCACAGAGAAGTTAAAGATATTAAGGATAAGAATTCAGACTTGAAAACTTTTAATGCAGCAGAAGAAGATAAAGCAACTAAGGAAGTATACGATCTCGTATCTGAAATTGATACATTAGTTGTATCATATTATGGTGATAAGGATTATGGGGAGCACGCGAAAGAGTTACGAGCAAAACTGGACTTAATCCTTGGAGATACAGACAATCCACATAAAATTACAAATGAACGTATTAAAAAAGAAATGATTGATGACTTAAATTCAATTATTGATGATTTCTTTATGGAAACTAAACAAAATAGACCGAAATCTATAACGAAATATAATCCTACAACACATAACTATAAAACAAATAGTGATAATAAACCTAATTTTGATAAATTAGTTGAAGAAACGAAAAAAGCAGTTAAAGAAGCAGATGATTCTTGGAAAAAGAAAACTGTCAAAAAATACGGAGAAACTGAAACAAAATCGCCAGTAGTAAAAGAAGAGAAGAAAGTTGAAGAACCTCAAGCACCTAAAGTTGATAACCAACAAGAGGTTAAAACTACGGCTGGTAAAGCTGAAGAAACAACACAACCAGTTGCACAACCATTAGTTAAAATTCCACAGGGCACAATTACAGGTGAAATTGTAAAAGGTCCGGAATATCCAACGATGGAAAATAAAACGGTACAAGGTGAAATCGTTCAAGGTCCCGATTTTCTAACAATGGAACAAAGCGGCCCATCATTAAGCAATAATTATACAAACCCACCGTTAACGAACCCTATTTTAGAAGGTCTTGAAGGTAGCTCATCTAAACTTGAAATAAAACCACAAGGTACTGAATCAACGTTAAAAGGTACTCAAGGAGAATCAAGTGATATTGAAGTTAAACCTCAAGCAACTGAAACAACAGAAGCTTCTCAATATGGTCCGAGACCGCAATTTAACAAAACACCTAAATATGTTAAATATAGAGATGCTGGTACAGGTATCCGTGAATACAACGATGGAACATTTGGATATGAAGCGAGACCAAGATTCAATAAGCCATCAGAAACAAATGCATATAACGTAACAACACATGCAAATGGTCAAGTATCATACGGAGCTCGTCCGACATACAAGAAGCCAAGCGAAACGAATGCATACAATGTAACAACACATGCAAACGGCCAAGTATCATACGGAGCTCGTCCGACACAAAACAAGCCAAGCAAAACAAACGCATATAACGTAACAACACATGGAAACGGCCAAGTATCATATGGCGCTCGCCCAACACAAAACAAGCCAAGCAAAACAAATGCATACAACGTAACAACACATGCAAACGGTCAAGTGTCATACGGAGCTCGCCCGACATACAAGAAGCCAAGTAAAACAAATGCATACAATGTAACAACACATGCAGATGGTACTGCGACATATGGGCCTAGAGTAACAAAATAA

>aureus_VC40

ATGAAAAAGCAAATAATTTCGCTAGGCGCATTAGCAGTTGCATCTAGCTTATTTACATGGGATAACAAAGCAGATGCGATAGTAACAAAGGATTATAGTGGGAAATCACAAGTTAATGCTGGGAGTAAAAATGGGACATTAATAGATAGCAGATATTTAAATTCAGCTCTATATTATTTGGAAGACTATATAATTTATGCTATAGGATTAACTAATAAATATGAATATGGAGATAATATTTATAAAGAAGCTAAAGATAGGTTGTTGGAAAAGGTATTAAGGGAAGATCAATATCTTTTGGAGAGAAAGAAATCTCAATATGAAGATTATAAACAATGGTATGCAAATTATAAAAAAGAAAATCCTCGTACAGATTTAAAAATGGCTAATTTTCATAAATATAATTTAGAAGAACTTTCGATGAAAGAATACAATGAACTACAGGATGCATTAAAGAGAGCACTGGATGATTTTCACAGAGAAGTTAAAGATATTAAGGATAAGAATTCAGACTTGAAAACTTTTAATGCAGCAGAAGAAGATAAAGCAACTAAGGAAGTATACGATCTCGTATCTGAAATTGATACATTAGTTGTATCATATTATGGTGATAAGGATTATGGGGAGCACGCGAAAGAGTTACGAGCAAAACTGGACTTAATCCTTGGAGATACAGACAATCCACATAAAATTACAAATGAACGTATTAAAAAAGAAATGATTGATGACTTAAATTCAATTATTGATGATTTCTTTATGGAAACTAAACAAAATAGACCGAAATCTATAACGAAATATAATCCTACAACACATAACTATAAAACAAATAGTGATAATAAACCTAATTTTGATAAATTAGTTGAAGAAACGAAAAAAGCAGTTAAAGAAGCAGATGATTCTTGGAAAAAGAAAACTGTCAAAAAATACGGAGAAACTGAAACAAAATCGCCAGTAGTAAAAGAAGAGAAGAAAGTTGAAGAACCTCAAGCACCTAAAGTTGATAACCAACAAGAGGTTAAAACTACGGCTGGTAAAGCTGAAGAAACAACACAACCAGTTGCACAACCATTAGTTAAAATTCCACAGGGCACAATTACAGGTGAAATTGTAAAAGGTCCGGAATATCCAACGATGGAAAATAAAACGGTACAAGGTGAAATCGTTCAAGGTCCCGATTTTCTAACAATGGAACAAAGCGGCCCATCATTAAGCAATAATTATACAAACCCACCGTTAACGAACCCTATTTTAGAAGGTCTTGAAGGTAGCTCATCTAAACTTGAAATAAAACCACAAGGTACTGAATCAACGTTAAAAGGTACTCAAGGAGAATCAAGTGATATTGAAGTTAAACCTCAAGCAACTGAAACAACAGAAGCTTCTCAATATGGTCCGAGACCGCAATTTAACAAAACACCTAAATATGTTAAATATAGAGATGCTGGTACAGGTATCCGTGAATACAACGATGGAACATTTGGATATGAAGCGAGACCAAGATTCAATAAGCCATCAGAAACAAATGCATATAACGTAACAACACATGCAAATGGTCAAGTATCATACGGAGCTCGTCCGACATACAAGAAGCCAAGCGAAACGAATGCATACAATGTAACAACACATGGAAACGGCCAAGTATCATACGGAGCTCGTCCGACACAAAACAAGCCAAGCAAAACAAACGCATATAACGTAACAACACATGGAAACGGCCAAGTATCATATGGCGCTCGCCCAACACAAAACAAGCCAAGCAAAACAAATGCATACAACGTAACAACACATGCAAACGGTCAAGTGTCATACGGAGCTCGCCCGACATACAAGAAGCCAAGTAAAACAAATGCATACAATGTAACAACACATGCAGATGGTACTGCGACATATGGGCCTAGAGTAACAAAATAA

>USA300

ATGAAAAAGCAAATAATTTCGCTAGGCGCATTAGCAGTTGCATCTAGCTTATTTACATGGGATAACAAAGCAGATGCGATAGTAACAAAGGATTATAGTGGGAAATCACAAGTTAATGCTGGGAGTAAAAATGGGACATTAATAGATAGCAGATATTTAAATTCAGCTCTATATTATTTGGAAGACTATATAATTTATGCTATAGGATTAACTAATAAATATGAATATGGAGATAATATTTATAAAGAAGCTAAAGATAGGTTGTTGGAAAAGGTATTAAGGGAAGATCAATATCTTTTGGAGAGAAAGAAATCTCAATATGAAGATTATAAACAATGGTATGCAAATTATAAAAAAGAAAATCCTCGTACAGATTTAAAAATGGCTAATTTTCATAAATATAATTTAGAAGAACTTTCGATGAAAGAATACAATGAACTACAGGATGCATTAAAGAGAGCACTGGATGATTTTCACAGAGAAGTTAAAGATATTAAGGATAAGAATTCAGACTTGAAAACTTTTAATGCAGCAGAAGAAGATAAAGCAACTAAGGAAGTATACGATCTCGTATCTGAAATTGATACATTAGTTGTATCATATTATGGTGATAAGGATTATGGGGAGCACGCGAAAGAGTTACGAGCAAAACTGGACTTAATCCTTGGAGATACAGACAATCCACATAAAATTACAAATGAACGTATTAAAAAAGAAATGATTGATGACTTAAATTCAATTATTGATGATTTCTTTATGGAAACTAAACAAAATAGACCGAAATCTATAACGAAATATAATCCTACAACACATAACTATAAAACAAATAGTGATAATAAACCTAATTTTGATAAATTAGTTGAAGAAACGAAAAAAGCAGTTAAAGAAGCAGATGATTCTTGGAAAAAGAAAACTGTCAAAAAATACGGAGAAACTGAAACAAAATCGCCAGTAGTAAAAGAAGAGAAGAAAGTTGAAGAACCTCAAGCACCTAAAGTTGATAACCAACAAGAGGTTAAAACTACGGCTGGTAAAGCTGAAGAAACAACACAACCAGTTGCACAACCATTAGTTAAAATTCCACAGGGCACAATTACAGGTGAAATTGTAAAAGGTCCGGAATATCCAACGATGGAAAATAAAACGGTACAAGGTGAAATCGTTCAAGGTCCCGATTTTCTAACAATGGAACAAAGCGGCCCATCATTAAGCAATAATTATACAAACCCACCGTTAACGAACCCTATTTTAGAAGGTCTTGAAGGTAGCTCATCTAAACTTGAAATAAAACCACAAGGTACTGAATCAACGTTAAAAGGTACTCAAGGAGAATCAAGTGATATTGAAGTTAAACCTCAAGCAACTGAAACAACAGAAGCTTCTCAATATGGTCCGAGACCGCAATTTAACAAAACACCTAAATATGTTAAATATAGAGATGCTGGTACAGGTATCCGTGAATACAACGATGGAACATTTGGATATGAAGCGAGACCAAGATTCAATAAGCCATCAGAAACAAATGCATATAACGTAACAACACATGCAAATGGTCAAGTATCATACGGAGCTCGTCCGACACAAAACAAGCCAAGCAAAACAAACGCATATAACGTAACAACACATGGAAACGGCCAAGTATCATATGGCGCTCGCCCAACACAAAACAAGCCAAGCAAAACAAATGCATACAACGTAACAACACATGCAAACGGTCAAGTGTCATACGGAGCTCGCCCGACATACAAGAAGCCAAGTAAAACAAATGCATACAATGTAACAACACATGCAGATGGTACTGCGACATATGGGCCTAGAGTAACAAAATAA

>TCH60

ATGAAAAAGCAAATAATTTCGCTAGGCGCATTAGCAGTTGCATCTAGCTTATTTACATGGGATAACAAAGCAGATGCGATAGTAACTAAAGATTATAGTAAAGAATCAAGAGTGAATGAGAACAGTAAATACGATACACCAATTCCAGATTGGTATCTAGGTAGTATTTTAAACAGATTAGGGGATCAAATATACTACGCTAAGGAATTAACTAATAAATACGAATATGGTGAGAAAGAGTATAAGCAAGCGATAGATAAATTGATGACTAGAGTTTTGGGAGAAGATCATTATCTATTAGAAAAAAAGAAAGCACAATATGAAGCATACAAAAAATGGTTTGAAAAACATAAAAGTGAAAATCCACATTCTAGTTTAAAAAAGATTAAATTTGACGATTTTGATTTATATAGATTAACGAAGAAAGAATACAATGAGTTACATCAATCATTAAAAGAAGCTGTTGATGAGTTTAATAGTGAAGTGAAAAATATTCAATCTAAACAAAAGGATTTATTACCTTATGATGAAGCAACTGAAAATCGAGTAACAAATGGAATATATGATTTTGTTTGCGAGATTGACACATTATACGCAGCATATTTTAATCATAGCCAATATGGTCATAATGCTAAAGAATTAAGAGCAAAGCTAGATATAATTCTTGGTGATGCTAAAGATCCTGTTAGAATTACGAATGAAAGAATAAGAAAAGAAATGATGGATGATTTAAATTCTATTATTGATGATTTCTTTATGGATACAAACATGAATAGACCATTAAACATAACTAAATTTAATCCGAATATTCATGACTATACTAATAAGCCTGAAAATAGAGATAACTTCGATAAATTAGTCAAAGAAACAAGAGAAGCAGTCGCAAACGCTGACGAATCTTGGAAAACAAGAACCGTCAAAAATTACGGTGAATCTGAAACAAAATCTCCTGTTGTAAAAGAAGAGAAGAAAGTTGAAGAACCTCAATTACCTAAAGTTGGAAACCAGCAAGAGGATAAAATTACAGTTGGTACAACTGAAGAAGCACCATTACCAATTGCGCAACCACTAGTTAAAATTCCACAGGGCACAATTCAAGGTGAAATTGTAAAAGGTCCGGAATATCTAACGATGGAAAATAAAACGTTACAAGGTGAAATCGTTCAAGGTCCAGATTTCCCAACAATGGAACAAAACAGACCATCTTTAAGCGATAATTATACTCAACCGACGACACCGAACCCTATTTTAAAAGGTATTGAAGGAAACTCAACTAAACTTGAAATAAAACCACAAGGTACTGAATCAACGTTAAAAGGTACTCAAGGAGAATCAAGTGATATTGAAGTTAAACCTCAAGCAACTGAAACAACAGAAGCATCACATTATCCAGCGAGACCTCAATTTAACAAAACACCTAAGTATGTGAAATATAGAGATGCTGGTACAGGTATCCGTGAATACAACGATGGAACATTTGGATATGAAGCGAGACCAAGATTCAACAAGCCAAGCGAAACAAATGCATACAACGTAACGACAAATCAAGATGGCACAGTATCATATGGCGCTCGCCCGACACAAAACAAACCAAGCGAAACAAATGCATACAACGTAACAACACATGCAAACGGCCAAGTATCATATGGCGCCCGCCCAACATACAAGAAGCCAAGCGAAACAAACGCATACAACGTAACGACAAATCAAGATGGCACAGTATCATATGGCGCTCGCCCGACACAAAACAAGCCAAGCGAAACAAACGCATATAACGTAACAACACATGCAAACGGCCAAGTATCATACGGAGCTCGTCCGACACAAAACAAGCCAAGCGAAACGAACGCATATAACGTAACAACACATGCAAACGGTCAAGTGTCATACGGAGCTCGCCCAACACAAAACAAGCCAAGTAAAACAAATGCATACAATGTAACAACACATGCAGATGGTACTGCGACATATGGTCCTAGAGTAACAAAATAA

>ED133

ATGAAAAAGCAAATAATTTCGCTAGGCGCATTAGCAGTTGCATCTAGCTTATTTACATGGGATAACAAAGCAGATGCGATAGTAACAAAGGATTATAGCAAAGAGTCAAGAGTGAATGAGAACAGTAAATATGGGACATTAATTTCAGACTGGTATTTAAAAGGGAGATTAACTAGTCTAGAATCTCAATTTATCAATGCATTGGATATTTTAGAGACATATCATTATGGCGAAAAAGAGTATAAAGATGCAAAAGATAAATTGATGACAAGAATTTTAGGGGAAGACCAATACCTTTTAGAAAGAAAAAAAGTGCAGTATGAGGAATACAAAAAATTATACCAAAAATATAAAGAAGAGAATCCAACCTCTAAAGTTAAAATGAAAACATTCGATCAATATACAATAGAAGATTTAACTATGAGGGAATATAATGAGTTAACAGAATCATTAAAAAGTGCTGTAAAAGACTTTGAGAAAGATGTTGAAAAAATAGAAAATCAACATCATGATTTGAAACCATTTACTGATGAAATGGAAGAGAAGGCTACTTCTAGAGTTGATGATTTAGCAAATAAAGCATATAGTGTTTATTTTGCATTTGTTAGGGATACACAACATAAAACTGAGGCATTAGAGTTAAAAGCGAAAGTAGATTTAGTTTTAGGTGATGAGGATAAACCGCATCGTATTTCTAATGAAAGAATTGAAAAAGAAATGATAAAAGATTTAGAATCTATTATTGAAGATTTCTTTATAGAAACTGGTTTAAATAAGCCTGGTAATATTACGAGTTATGATAGTAGTAAACATCACTATAAAAATCACAGTGAAGGTTTTGAGGCTCTAGTCAAAGAAACAAGAGAAGCAGTAGCAAACGCTGACGAATCTTGGAAAACTAAAACTGTAAAAAAATACGGTGAATCTGAAACAAAATCTCCTGTTGTAAAAGAAGAGAACAAAGTTGAAGACCCTCAATCACCTAAATTTGATAACCAACAAGAGGTTAAAACTACGGCTGGTAAAGCTGAAGAAACAACACAACCAGTTGCACAACCATTAGTTAAAATTCCACAGGGCACAATTACAGGTGAAATTGTGAAAGGTCCGGAATATCCAACGATGGAAAATAAAACGTTACAAGGTGAAATCGTTCAAGGTCCAGATTTCCCAACAATGGAACAAAGCGGTCCATCTTTAAGCGACAATTATACTCAACCGACGACACCGAACCCTATTTTAGAAGGTCTTGAAGGTAGCTCATCTAAACTTGAAATAAAACCACAAGGTACTGAATCAACGTTGAAAGGTATTCAAGGAGAATCAAGTGATATTGAAGTTAAACCTCAAGCAACTGAAACAACAGAAGCTTCTCAATATGGTCCGAGACCGCAATTTAACAAAACACCTAAGTATGTGAAATATAGAGATGCTGGTACAGGTATTCGTGAATACAACGATGGAACATTTGGATATGAAGCGAGACCAAGATTCAACAAGCCATCAGAAACAAACGCATACAACGTAACGACAAATCAAGATGGCACAGTATCATACGGCGCCCGCCCAACACAAAACAAGGCATCAGAAACAAACGCATATAACGTAACAACACATGCAAACGGCCAAGTATCATACGGAGCTCGCCCAACACAAAAGAAGCCAAGCGAAACAAATGCATATAACGTAACAACACATGCAAACGGCCAAGTATCATATGGCGCCCGCCCGACATACAACAAGCCAAGCGAAACAAATGCATATAACGTAACAACACACGGAAATGGCCAAGTATCATATGGAGCTCGTCCGACATACAAGAAACCAAGTAAAACAAATGCATATAACGTAACAACACATGCAAACGGCCAAGTGTCATACGGAGCTCGCCCAACACAAAACAAGCCAAGCGAAACAAACGCATATAACGTAACAACACATGCAAATGGCCAAGTATCATACGGAGCTCGCCCAACACAAAACAAGCCAAGCGAAACAAACGCATATAACGTAACAACACACGGAAACGGTCAAGTGTCATACGGCGCTCGTCCGACATACAACAAGCCAAGTAAAACAAATGCATACAATGTAACAACACATGCAGATGGTACTGCGACATATGGTCCTAGAGTAACAAAATAA

>aureus_CN1

ATGAAAAAGCAAATAATTTCGCTAGGCGCATTAGCAGTTGCATCTAGCTTATTTACATGGGATAACAAAGCAGATGCGATAGTAACAAAGGATTATAGTAAAGAATCAAGAGTGAAAGAAGAGAGTAAATATGATTCGCCAATGTCAAATTGGTATTATTGGGGAAAGGTTAAATCCTTGGAGTCACAATTTGCAGATGCGATAGATATTATAGAAGATTATCAATATGGGGAAAAAGAGTATAAAGACGCCAAAGACAAATTGATGACTAGGGTATTAGGTGAAGACCAATACTTATTAAAGAAAAAAATAGATGAATATAAACAATATAGAGAAAGATATTTAAAAGCTGGTTTAAGCCCGGTGAAATTTTATGATTATAACCTTTATGATTTTACAATGAAAGAATATAATGATATCCATCACTCTCTAAAAGGTGCAGTGGAAGAGTTCTATAAAGAAGTTAAACATATTCAATCAAAGAATTCGGATTTACAAACTTATGACAAGAAAACTGAAGATGAAGAAACTGATAAAGTATATTCATTAGTTAGTGAAATTGATACTCTTGTTGTAACATATTATGGAGATAAGGATTATGGGGAGCACGCTAAAGAGTTGAGAGCTAAGCTAGATATTATTCTTGGAGAAGAAAAAAAGCCCAATAGAATAACTAATGAACGTATTAGAAAAGAAATGACCGATGATTTAAATTCTATTATCGATGACTTCTTTATGGAAACTGGACAAAACAGACCGGTTAAAATCACTAAATATAATCCAAATATTCATAGTCCTAAAGATAACAAAGAAAACTTCGATGCTTTAGTTAAAGAAACAAGAGAAGCAGTTGAAAAAGCAGATGATTCTTGGAAAAAGAAAACTGTCAAAAAATACGGAGAAACTGAAACAAAATCGCCAGTAGTAAAAGAAGAGAAGAAAGTTGAAGAACCTCAAGCACCTAAAGTTGATAACCAACAAGAAGTTAAAACTACTGCTGGTAAAGCTGAAGAAACAACACAACCAGTGGCACAGCCATTAGTAAAAATTCCACAAGGAACAATCTATGGTGAAACTGTAAAAGGTCCAGACTATCCAACTATGGAAAATAAAACGTTACAAGGTGTAATTGTTCAAGGTCCAGATTTCCCAACAATGGAACAAAGCGGCCCATCATTAAGCAATAATTATACAAACCCACCGTTAACGAACCCTATTTTAGAAGGTCTTGAAGGTAGCTCATCTAAACTTGAAATAAAACCACAAGGTACTGAATCAACGTTAAAAGGTACTCAAGGAGAATCAAGTGATATTGAAGTTAAACCTCAAGCAACTGAAACAACAGAAGCTTCTCAATATGGTCCGAGACCGCAATTTAACAAAACACCTAAATATGTTAAATATAGAGATGCTGGTACAGGTATCCGTGAATACAACGATGGAACATTTGGATATGAAGCGAGACCAAGATTCAATAAGCCATCAGAAACAAACGCATACAACGTAACGACAAATCAAGATGGCACAGTAACATATGGCGCTCGTCCAACACAAAACAAGCCAAGTAAAACGAACGCATATAACGTAACAACACATGCAAACGGCCAAGTATCATATGGCGCTCGCCCAACACAAAACAAGCCAAGCAAAACAAATGCATACAACGTAACAACACATGCAAATGGTCAAGTATCATATGGCGCTCGCCCGACACAAAACAAGCCAAGCAAAACAAATGCATATAACGTAACAACACATGCAAATGGTCAAGTATCATACGGAGCTCGCCCGACATACAAGAAGCCAAGCGAAACAAATGCATACAACGTAACAACACATGCAAATGGTCAAGTATCATATGGCGCTCGCCCGACACAAAACAAGCCAAGCGAAACAAACGCATATAATGTAACAACACATGCAGATGGTACTGCGACATATGGGCCTAGAGTAACAAAATAA

>ATCC_12600

ATGAAAAAGCAAATAATTTCGCTAGGCGCATTAGCAGTTGCATCTAGCTTATTTACATGGGATAACAAAGCAGATGCGATAGTAACAAAGGATTATAGTGGGAAATCACAAGTTAATGCTGGGAGTAAAAATGGGACATTAATAGATAGCAGATATTTAAATTCAGCTCTATATTATTTGGAAGACTATATAATTTATGCTATAGGATTAACTAATAAATATGAATATGGAGATAATATTTATAAAGAAGCTAAAGATAGGTTGTTGGAAAAGGTATTAAGGGAAGATCAATATCTTTTGGAGAGAAAGAAATCTCAATATGAAGATTATAAACAATGGTATGCAAATTATAAAAAAGAAAATCCTCGTACAGATTTAAAAATGGCTAATTTTCATAAATATAATTTAGAAGAACTTTCGATGAAAGAATACAATGAACTACAGGATGCATTAAAGAGAGCACTGGATGATTTTCACAGAGAAGTTAAAGATATTAAGGATAAGAATTCAGACTTGAAAACTTTTAATGCAGCAGAAGAAGATAAAGCAACTAAGGAAGTATACGATCTCGTATCTGAAATTGATACATTAGTTGTATCATATTATGGTGATAAGGATTATGGGGAGCACGCGAAAGAGTTACGAGCAAAACTGGACTTAATCCTTGGAGATACAGACAATCCACATAAAATTACAAATGAACGTATTAAAAAAGAAATGATTGATGACTTAAATTCAATTATTGATGATTTCTTTATGGAAACTAAACAAAATAGACCGAAATCTATAACGAAATATAATCCTACAACACATAACTATAAAACAAATAGTGATAATAAACCTAATTTTGATAAATTAGTTGAAGAAACGAAAAAAGCAGTTAAAGAAGCAGATGATTCTTGGAAAAAGAAAACTGTCAAAAAATACGGAGAAACTGAAACAAAATCGCCAGTAGTAAAAGAAGAGAAGAAAGTTGAAGAACCTCAAGCACCTAAAGTTGATAACCAACAAGAGGTTAAAACTACGGCTGGTAAAGCTGAAGAAACAACACAACCAGTTGCACAACCATTAGTTAAAATTCCACAGGGCACAATTACAGGTGAAATTGTAAAAGGTCCGGAATATCCAACGATGGAAAATAAAACGGTACAAGGTGAAATCGTTCAAGGTCCCGATTTTCTAACAATGGAACAAAGCGGCCCATCATTAAGCAATAATTATACAAACCCACCGTTAACGAACCCTATTTTAGAAGGTCTTGAAGGTAGCTCATCTAAACTTGAAATAAAACCACAAGGTACTGAATCAACGTTAAAAGGTACTCAAGGAGAATCAAGTGATATTGAAGTTAAACCTCAAGCAACTGAAACAACAGAAGCTTCTCAATATGGTCCGAGACCGCAATTTAACAAAACACCTAAATATGTTAAATATAGAGATGCTGGTACAGGTATCCGTGAATACAACGATGGAACATTTGGATATGAAGCGAGACCAAGATTCAATAAGCCATCAGAAACAAATGCATATAACGTAACAACACATGCAAATGGTCAAGTATCATACGGAGCTCGTCCGACATACAAGAAGCCAAGCGAAACGAATGCATACAATGTAACAACACATGCAAACGGCCAAGTATCATACGGAGCTCGTCCGACACAAAACAAGCCAAGCAAAACAAACGCATATAACGTAACAACACATGGAAACGGCCAAGTATCATATGGCGCTCGCCCAACACAAAACAAGCCAAGCAAAACAAATGCATACAACGTAACAACACATGCAAACGGTCAAGTGTCATACGGAGCTCGCCCGACATACAAGAAGCCAAGTAAAACAAATGCATACAATGTAACAACACATGCAGATGGTACTGCGACATATGGGCCTAGAGTAACAAAATAA

>Stp58

ATGAAAAAGCAAATAATTTCGCTAGGCGCATTAGCAGTTGCATCTAGCTTATTTACATGGGATAACAAAGCAGATGCGATAGTAACAAAGGATTATAGTAAAGAATCAAGAGTGAATGAGAACAGTAAATATGATTCGCCAATGTCAAATTGGTATTATTGGGGAAAGGTTAAATCCTTGGAGTCACAATTTGCAGATGCAATAGATATTATAGAAGATTATCAATATGGTGAAAAAGAATATAAAGATGCAAAAGATAAACTAATGACTAGAATACTAGGTGAGGACCAATACTTATTAAAGAAAAAAATAGAAGAATATAAACAATATAGAGAAAGATATTTAAAAGCTGGATTAAGTCCTGTGAAATTTTATGATTACAATCTTTATGATTTTACAATGAAAGAATATAATGATATCCATCAGTCTTTAAAAGATGCAGTAGAAGAGTTCTATCAAGAAGTTAAACATATTCAATCAAAGAATTCGGATTTACAAACTTATGATAAGAAAACTGAAGATAAAGAAACTGATAATGTATACTCTTTAGTTAGTGAAATTGATACTATTGTTGCAACATATTATGGAGATAAAAATCATGGAGAGCATGCTAAAGAGTTGAGAGCTAAGCTAGATATTATTCTTGGAGAAGAAAAAAAGCCAAATAGAATAACTAATGAACGTATTAGAAAAGAAATGACTGATGATTTGAATTCTATTATCGATGACTTCTTTATGGAAACTGGGCAAAACAGACCGGTTAAAATCACTAAATATAATCCAAATATTCATAGCCCTAAAGATAACAAAGAAAGCTTCGATAAATTAGTTGAAGAAACGAAAAAAGCAGTTAAAGAAGCAGATGAGTCTTGGAAAACTAAAACTGTCAAAACATACGGTGAAACTGAAACAAAAGCACATGTTGTAAAAGAAGAGAAGAAAGTTGAAGAACCTCAATTACCTAAAGTTGGAAACCAGCAAGAGGATAAAACTACAGTTGGTACAACTGAAAAAGCACCATTACCAATTGCGCAACCACTAGTTAAATTACCACAAATTGGGACTCAAGGTAAAATTGTAGAAGGGCCAAAATACCCAACGATGGAACAGCACACAATCTATGGTGAAATTGTAAAAGGTCCCGACTATCTAACGATGGAAAATAAAACGTTACAAGGTGAAATCGTTCAAGGTCCAGATTTCCCAACAATGGAACAAAACAGACCAGCACTAAGCGATAATTATACAAACCCAACGTTAACGAACCCTATTTTAAAAGGTATTGAAGGAAACTCAACTAATCTTGAAATAAAACCACAAGGTACTGAATCAACGTTGAAAGGTATTCAAGGAGAATCAAGTGATATTGAAGTTAAACCTCAAGCAACTGAAACAACAGAAGCATCACATTATCCAGCGAGACCGCAATTTAACAAAACACCTAAGTATGTGAAATATAGAGATGCTGGTACAGGTATCCGTGAATACAACGATGGAACATTTGGATATGAAGCGAGACCAAGATTCAACAAGCCATCAGAAACAAACGCATACAACGTAATGACAAATCAAGATGGCACAGTATCATACGGCGCCCGCCCAACACAAAACAAGGCATCAGAAACAAACGCATATAACGTAACAACACATGCAAACGGCCAAGTATCATACGGAGCTCGCCCAACACAAAAGAAGCCAAGCGAAACAAATGCATATAACGTAACAACACATGCAAACGGCCAAGTATCATATGGCGCCCGCCCGACATACAAGAAGCCAAGTGAAACAAATGCATATAACGTAACAACACATGCAAATGGCCAAGTATCATATGGGGCTCGCCCAACACAAAACAAGCCAAGCAATACAAACGCATATAACGTAACAACACATGCAAACGGCCAAGTATCATATGGGGCTCGCCCGACACAAAACAAGGCATCAGAAACAAACGCATATAACGTAACAACACATGCAAACGGCCAAGTATCATACGGAGCTCGCCCGACACAAAACAAGCCAAGCGAAACAAACGCATATAACGTAACAACACACGGAAACGGTCAAGTGTCATACGGCGCTCGTCCGACATACAACAAGCCAAGTAAAACAAATGCATACAATGTAACAACACATGCAGATGGTACTGCGACATATGGTCCTAGAGTAACAAAATAA

>Stp25

ATGAAAAAGCAAATAATTTCGCTAGGCGCATTAGCAGTTGCATCTAGCTTATTTACATGGGATAACAAAGCAGATGCGATAGTAACAAAGGATTATAGTAAAGAATCAAGAGTGAATGAGAACAGTAAATATGATTCGCCAATGTCAAATTGGTATTATTGGGGAAAGGTTAAATCCTTGGAGTCACAATTTGCAGATGCAATAGATATTATAGAAGATTATCAATATGGTGAAAAAGAATATAAAGATGCAAAAGATAAACTAATGACTAGAATACTAGGTGAGGACCAATACTTATTAAAGAAAAAAATAGAAGAATATAAACAATATAGAGAAAGATATTTAAAAGCTGGATTAAGTCCTGTGAAATTTTATGATTACAATCTTTATGATTTTACAATGAAAGAATATAATGATATCCATCAGTCTTTAAAAGATGCAGTAGAAGAGTTCTATCAAGAAGTTAAACATATTCAATCAAAGAATTCGGATTTACAAACTTATGATAAGAAAACTGAAGATAAAGAAACTGATAATGTATACTCTTTAGTTAGTGAAATTGATACTATTGTTGCAACATATTATGGAGATAAAAATCATGGAGAGCATGCTAAAGAGTTGAGAGCTAAGCTAGATATTATTCTTGGAGAAGAAAAAAAGCCAAATAGAATAACTAATGAACGTATTAGAAAAGAAATGACTGATGATTTGAATTCTATTATCGATGACTTCTTTATGGAAACTGGGCAAAACAGACCGGTTAAAATCACTAAATATAATCCAAATATTCATAGCCCTAAAGATAACAAAGAAAGCTTCGATAAATTAGTTGAAGAAACGAAAAAAGCAGTTAAAGAAGCAGATGAGTCTTGGAAAACTAAAACTGTCAAAACATACGGTGAAACTGAAACAAAAGCACATGTTGTAAAAGAAGAGAAGAAAGTTGAAGAACCTCAATTACCTAAAGTTGGAAACCAGCAAGAGGATAAAACTACAGTTGGTACAACTGAAAAAGCACCATTACCAATTGCGCAACCACTAGTTAAATTACCACAAATTGGGACTCAAGGTAAAATTGTAGAAGGGCCAAAATACCCAACGATGGAACAGCACACAATCTATGGTGAAATTGTAAAAGGTCCCGACTATCTAACGATGGAAAATAAAACGTTACAAGGTGAAATCGTTCAAGGTCCAGATTTCCCAACAATGGAACAAAACAGACCAGCACTAAGCGATAATTATACAAACCCAACGTTAACGAACCCTATTTTAAAAGGTATTGAAGGAAACTCAACTAATCTTGAAATAAAACCACAAGGTACTGAATCAACGTTGAAAGGTATTCAAGGAGAATCAAGTGATATTGAAGTTAAACCTCAAGCAACTGAAACAACAGAAGCATCACATTATCCAGCGAGACCGCAATTTAACAAAACACCTAAGTATGTGAAATATAGAGATGCTGGTACAGGTATCCGTGAATACAACGATGGAACATTTGGATATGAAGCGAGACCAAGATTCAACAAGCCATCAGAAACAAACGCATACAACGTAATGACAAATCAAGATGGCACAGTATCATACGGCGCCCGCCCAACACAAAACAAGGCATCAGAAACAAACGCATATAACGTAACAACACATGCAAACGGCCAAGTATCATACGGAGCTCGCCCAACACAAAAGAAGCCAAGCGAAACAAATGCATATAACGTAACAACACATGCAAACGGCCAAGTATCATATGGCGCCCGCCCGACATACAAGAAGCCAAGTGAAACAAATGCATATAACGTAACAACACATGCAAATGGCCAAGTATCATATGGGGCTCGCCCAACACAAAACAAGCCAAGCAATACAAACGCATATAACGTAACAACACATGCAAACGGCCAAGTATCATATGGGGCTCGCCCGACACAAAACAAGGCATCAGAAACAAACGCATATAACGTAACAACACATGCAAACGGCCAAGTATCATACGGAGCTCGCCCGACACAAAACAAGCCAAGCGAAACAAACGCATATAACGTAACAACACACGGAAACGGTCAAGTGTCATACGGCGCTCGTCCGACATACAACAAGCCAAGTAAAACAAATGCATACAATGTAACAACACATGCAGATGGTACTGCGACATATGGTCCTAGAGTAACAAAATAA

>C-1C

ATGAAGAGAATTAGTAAAGATATATGGGCAGTATTTAAATTACTGTATCAAAATAAAGGGCGTTTTAGCATTAATGCCTTACTATTGCAGTTAATCATGATTTTTATTAGTAGTACATACTTAATTTTACTATTTAATATGATGTTAAAAGTAGCTGGGCAAAGCCAACTTACGATTAACAATTGGACGGAAATCGTAAGTCATCCTGCCAGTGTGATACTTCTTATTATATTCATATTAAGTGTTGCTTTTCTGATTTATGTAGAGTTTTCATTGTTAGTTTATATGATTTATGCCGGCTTTGATCGACAGATTATTACATTTAAATCCATTTTTAAAAATGCCTTTGTAAATGTGCGTAAACTCATAGGTGTACCAGTTATTTTCTTTGTCATTTATTTAATGTTAATGATACCCATTGCCAACCTAGGACTAAGTTCAGTATTAACAAAAAATATTTACATACCTAAATTTTTAACGGAAGAACTTATGAAAACGACGAAAGGTATAATCATTTACGGTACCTTTATGATTGCTGTATTTATATTAAACTTTAAATTAATATTTACTTTACCGTTAACGATTTTAAACCGCCAGTCGTTATTTAAAAATATGAGACTAAGTTGGCAAATTACGAAGCGAAATAAGTTTCGACTTGTTATAGAAATAGTTATATTGGAACTCATCATTGGTGCGATTTTAACATTAATTATTTCAGGAGCAACATATCTTGCTATTTGTGTAGATGAAGAAGGAGATAAGTTTTTAGTCTCATCAATTTTATTTGTTGTATTGAAAAGCTCATTGTTCTTCTATTATTTATTTACGAAATTATCATTAATCAGTGTGTTAGTACTGCACTTAAAACAAGAGAATGTATTAGACCAACCGGGCTTAGAATTTAAATACCCAAAACCGAAACGGAAGTCTAGGTTCTTTATAATTTCAATGGTGCTTGCAGTGACATGTTTTATCGGTTATAACATGTACTTACTTTACAATAATACTATCAATACAAATATTTCTATTATTGGCCATCGTGGTTTCGAAGATAAAGGCGTTGAAAATTCTATTCCGTCATTGAAAGCTGCTGCAAAAGCGAATGTCGAATACGTTGAGTTAGATACAATTATGACGAAAGATAAACAATTTGTTGTTAGTCATGATAACAATTTGAAACGTTTAACAGGTGTTAATAAAAACATTTCTGAATCTAATTTCAAAGATGTCGTCGGTTTGAAAATGCGTCAAAATGGACATGAAGCAAAACTTGTATCCTTAGACGAATTTATTGAAACGGCTAAACAATCAAATGTGAAACTACTAGTAGAGTTAAAGCCACATGGTAAAGAACCAGCAGATTATGCACAACGTGTTATTGATATTTTGAAAAAGCATGGTGTTGAACATCAATATCGTGTGATGTCATTGGATTATGATGTGATGACTAAGTTGAAAAAAGAAGCGCCATATCTCAAGTGTGGTTATATCATTCCGTTGCAGTTTGGTCATTTTAAAGAAACATCATTAGATTTCTTTGTCATCGAAGATTTTTCTTATTCGCCAAGACTTGTTAATCAAGCGCACTTGGAAAATAAAGAAGTCTATACTTGGACTATTAACGGCGAAGAAGATTTAACGAAATACTTACAAACCAATGTTGATGGTATTATCACAGATGACCCAGCATTAGCTGATCAGATTAAAGAAGAAAAGAAAGACGAAACATACTTCGATCGTTCTATAAGAATTTTGTTTGAATAATATAAACAAAGACCTCTAAAGTTATCAAGATGATACTTTCAGAGGTCTTTTTAACGTTGCCATCTATGGGGATAGGCAATCGTTTCATTCGTTTATAATCATATGACAAGGATTTATAAGGCAATTTGGCGTCACAAACACTTACATGATTTATTAGTGAATTATTAATTGTTTTGTGAATGCAAAGGGTTAGAAATTGAAACGTAAATACTTTCTAATCTATGTTTCGCTTTAGTCATTTGATCCAAATTTTTAGTGCGTATAGCTGATTTAGCAATATAGTGCGCAGCTAAAATGTCGCGTTTTTGATACGCATCTAAATTTAGGTACGATAATTTATTAAAGTCAGTGTTTGCTATTAATTCATGTAATTGATCTACAAGCGCTTGATGTTGATACGTATGTGATGTAGTTTCAGATTTGCTTGCTAATTTAATACCAGTCGTATCAAGGAGCGCCGCTTTAATACCAGCAACTAAATATGTTTTGATTTTCATTTGTGTTGTCATGCTTTGTTACTCCTTTGATGTACATTAATCAAAAAAATTATACACTATTGTATATTGCAAAGCTAATTAACTATAACAAAAAGATAGTTAATGCTTTGTTTATTCTAGTTAATATATAGTTAATGTCTTTTAATATTTTGTTTCTTTAATGTAGATTGGGCAATTACATTTTGGAGGAATTAAAAAATTATGAAAAAGCAAATAATTTCGCTAGGCGCATTAGCAGTTGCATCTAGCTTATTTACATGGGATAACAAAGCAGATGCGATAGTAACAAAGGATTATAGTAAAGAATCAAGAGTGAAAGAAGAGAGTAAATATGATTCGCCAATGTCAAATTGGTATTATTGGGGAAAGGTTAAATCCTTGGAGTCACAATTTGCAGATGCGATAGATATTATAGAAGATTATCAATATGGGGAAAAAGAGTATAAAGACGCCAAAGACAAATTGATGACTAGGGTATTAGGTGAAGACCAATACTTATTAAAGAAAAAAATAGATGAATATAGACAATATAGAGAAAGATATTTAAAAGCTGGTTTAAGCCCGGTGAAATTTTATGATTATAACCTTTATGATTTTACAATGAAAGAATATAATGATATCCATCACTCTCTAAAAGGTGCAGTGGAAGAGTTCTATAAAGAAGTTAAACATATTCAATCAAAGAATTCGGATTTACAAACTTATGACAAGAAAACTGAAGATGAAGAAACTGATAAAGTATATTCATTAGTTAGTGAAATTGATACTCTTGTTGTAACATATTATGGAGATAAGGATTATGGGGAGCACGCTAAAGAGTTGAGAGCTAAGCTAGATATTATTCTTGGAGAAGAAAAAAAGCCCAATAGAATAACTAATGAACGTATTAGAAAAGAAATGACCGATGATTTAAATTCTATTATCGATGACTTCTTTATGGAAACTGGACAAAACAGACCGGTTAAAATCACTAAATATAATCCAAATATTCATAGTCCTAAAGATAACAAAGAAAACTTCGATGCTTTAGTTAAAGAAACAAGAGAAGCGGTTGCTAAGGCTGATGATTCTTGGAAAAATAAAACTGTCAAAAAATACGAGGAAACTGTAACAAAATCTCCTGTTGTAAAAGAAGAGAAGAAAGTTGAAGAACCTCAATTACCTAAAGTTGGAAACCAGCAAGAGGTTAAAACTACGGCTGGTAAAGCTGAAGAAACAACACAACCAGTTGCACAACCATTAGTTAAAATTCCACAGGGCACAATTACAGGTGAAATTGTAAAAGGTCCGGAATATCCAACGATGGAAAATAAAACGTTACAAGGTGAAATCGTTCAAGGTCCCGATTTTCTAACAATGGAACAAAACAGACCATCTTTAAGCGATAATTATACTCAACCGACGACACCGAACCCTATTTTAGAAGGTCTTGAAGGTAGCTCATCTAAACTTGAAATAAAACCACAAGGTACTGAATCAACGTTAAAAGGTACTCAAGGAGAATCAAGTGATATTGAAGTTAAACCTCAAGCAACTGAAACAACAGAAGCTTCTCAATATGGTCCGAGACCGCAATTTAACAAAACACCTAAGTATGTTAAATATAGAGATGCTGGTACAGGTATCCGTGAATACAACGATGGAACATTTGGATATGAAGCGAGACCAAGATTCAATAAGCCATCAGAAACAAACGCATACAACGTAACGACAAATCAAGATGGCACAGTAACATATGGCGCTCGTCCAACACAAAACAAGCCAAGTAAAACGAACGCATATAACGTAACAACACACGGAAACGGCCAAGTATCATATGGCGCTCGCCCGACACAAAACAAGCCAAGTAAAACAAATGCATATAACGTAACAACACATGCAAATGGTCAAGTATCATATGGAGCTCGTCCGACATACAAGAAGCCAAGCGAAACGAATGCATACAATGTAACAACACATGCAAACGGCCAAGTATCATACGGCGCTCGTCCGACACAAAACAAGGCAAGCGAAACAAACGCATATAACGTAACAACACATGCAAACGGTCAAGTGTCATACGGAGCTCGCCCGACATACAAGAAGCCAAGCGAAACAAATGCATACAATGTAACAACACATGCAGATGGTACTGCGACATATGGGCCTAGAGTAACAAAATAAATTTATAACTCTATCCAAAGACATACAGTCAATACAAAGAATTATGTATCTATACAACAGTAATCATGCATTCTATGATGCTTCTAACTGAATTAAAGCATCGAACAATCGGAAGCATATTTCTAAATTATTTATTCATTATAGTCTTAAACATAACATGACCTAATATATTACTAACCTATTAAAATAAACCACGCACATCTAAGTGATATACGACAATCACAGCAATAATAATTGCTTTAAAAAGTCGTACCGAACTGGAACTTACAAGTCTAGTTCGAACACACACTGATGTGAGTGGTTTTCTTTATTTTAAACATGAACAATCAGATAAGTTACTAGCATTAGCAAATATTATTAAATCAAAGGGCTTCGATTCATAAAATTTAAAACAATGATTAAAATTAGACGTGTAAATGTTAAATTCTAAAACGGAAATACCCACCATCCCATTAAACCACTTTTTTGTTCAATCACTATGTTTCACATAGCTTCAATAATAAAACGAAACTGCTTCAACCCGCTTCAACTTCAGCCTACTTCAATAACCAAACGAATCCGCTTCATCCAAAATCAACCATTCTAACGCACATATTCAAATATAGCAGCTGCACCCATACCGACACCAATACACATCGTAACCATGCCGTAACGGCTATCGGGACGTCTACCCATTTCATTAAGTAAACGCGCGGTTAACATTGCGCCTGTAGCACCTAATGGATGACCTAAAGCAATAGCGCCACCATTCACATTCGTACGTGATATATCTAGACCTACTTCTTTAATAGATGCAATTGTTTGAGAAGCAAATGCTTCGTTCAATTCGATCAAATCAATGTCTTCAACAGATAGATTGCTGAGTGACAATACTTCAGGAATCGCATATGCAGGCCCAATACCCATAATTTTCGGGTCAACGCCTACTGCCTTATAACCAACGAATCGTGCAATAGGTGTCACGCCTAGTTCTTTCACTTTATCTCCAGACATTAAAACTACAAATCCTGCACCATCAGAAAGTGGGGCAGATGTTCCCGCAGTCACAGTGCCGTCAGCTTTAAATACTGTACGTAATTTGGCTAATGCCTCCATCGTGGTGTCAGGGCGTATAAATTCATCTTGGTCAAAGATATTTGTGTGTACTTTTGGTCCTGCGTTTGTATATTCAACTGAGTTTACTTGTATTGGGATAATTTCATCGTTGAACCGACCATCACGTTGTGCTTCATAGGCACGTTGATGACTTCTGACAGCATAAGCATCTTGATCTTCGCGTGATACGTCAAATTGGGATGCTACATTTTCAGCAGTTAAACCCATAGGATATGACGCACCTATATCATCATATTGTAAGGTAGGATTGTTTGTGGGCTCGTTGCCACCCATTGGTACGGCACTCATCAATTCAACGCCACCAGCTACAAGTATATCTCCTTGACCAGCCATAATTTGATTGGCTGCAATCGCGATGGTTTGTAATCCTGATGAGCAGTAGCGATTCACTGTTTGACCCGGTACCGTGTCAGATAATCCCGTAAGCAATGCAATCGTTCGTGCAATGTTTTGTCCTTGTAATCCTTCTGGAAAAGCCGTACCAACAATGACATCTTCAATCATATTCTTATTGAATTTTCCGTCAATACGTTTCAATACGCCTTGTAATACTTTGGCTGCGACATCATCAGGTCTTTCGTGGAATAATGCGCCTTGCTTTGCTTTCGCTGCGGCTGAACGCCCATAAGCTACAATGTATGCTTCTTGCAT

>JCSC7638

ATGAAAAAGCAAATAATTTCGCTAGGCGCATTAGCAGTTGCATCTAGCTTATTTACATGGGATAACAAAGCAGATGCGATAGTAACAAAGGATTATAGTAAAGAATCAAGAGTGAAAGAAGAGAGTAAATATGATTCGCCAATGTCAAATTGGTATTATTGGGGAAAGGTTAAATCCTTGGAGTCACAATTTGCAGATGCGATAGATATTATAGAAGATTATCAATATGGGGAAAAAGAGTATAAAGACGCCAAAGACAAATTGATGACTAGGGTATTAGGTGAAGACCAATACTTATTAAAGAAAAAAATAGATGAATATAGACAATATAGAGAAAGATATTTAAAAGCTGGTTTAAGCCCGGTGAAATTTTATGATTATAACCTTTATGATTTTACAATGAAAGAATATAATGATATCCATCACTCTCTAAAAGGTGCAGTGGAAGAGTTCTATAAAGAAGTTAAACATATTCAATCAAAGAATTCGGATTTACAAACTTATGACAAGAAAACTGAAGATGAAGAAACTGATAAAGTATATTCATTAGTTAGTGAAATTGATACTCTTGTTGTAACATATTATGGAGATAAGGATTATGGGGAGCACGCTAAAGAGTTGAGAGCTAAGCTAGATATTATTCTTGGAGAAGAAAAAAAGCCCAATAGAATAACTAATGAACGTATTAGAAAAGAAATGACCGATGATTTAAATTCTATTATCGATGACTTCTTTATGGAAACTGGACAAAACAGACCGGTTAAAATCACTAAATATAATCCAAATATTCATAGTCCTAAAGATAACAAAGAAAACTTCGATGCTTTAGTTAAAGAAACAAGAGAAGCGGTTGCTAAGGCTGATGATTCTTGGAAAAATAAAACTGTCAAAAAATACGAGGAAACTGTAACAAAATCTCCTGTTGTAAAAGAAGAGAAGAAAGTTGAAGAACCTCAATTACCTAAAGTTGGAAACCAGCAAGAGGTTAAAACTACGGCTGGTAAAGCTGAAGAAACAACACAACCAGTTGCACAACCATTAGTTAAAATTCCACAGGGCACAATTACAGGTGAAATTGTAAAAGGTCCGGAATATCCAACGATGGAAAATAAAACGTTACAAGGTGAAATCGTTCAAGGTCCCGATTTTCTAACAATGGAACAAAACAGACCATCTTTAAGCGATAATTATACTCAACCGACGACACCGAACCCTATTTTAGAAGGTCTTGAAGGTAGCTCATCTAAACTTGAAATAAAACCACAAGGTACTGAATCAACGTTAAAAGGTACTCAAGGAGAATCAAGTGATATTGAAGTTAAACCTCAAGCAACTGAAACAACAGAAGCTTCTCAATATGGTCCGAGACCGCAATTTAACAAAACACCTAAGTATGTTAAATATAGAGATGCTGGTACAGGTATCCGTGAATACAACGATGGAACATTTGGATATGAAGCGAGACCAAGATTCAATAAGCCATCAGAAACAAACGCATACAACGTAACGACAAATCAAGATGGCACAGTAACATATGGCGCTCGTCCAACACAAAACAAGCCAAGTAAAACGAACGCATATAACGTAACAACACACGGAAACGGCCAAGTATCATATGGCGCTCGCCCGACACAAAACAAGCCAAGTAAAACAAATGCATATAACGTAACAACACATGCAAATGGTCAAGTATCATATGGAGCTCGTCCGACATACAAGAAGCCAAGCGAAACGAATGCATACAATGTAACAACACATGCAAACGGCCAAGTATCATACGGCGCTCGTCCGACACAAAACAAGGCAAGCGAAACAAACGCATATAACGTAACAACACATGCAAACGGTCAAGTGTCATACGGAGCTCGCCCGACATACAAGAAGCCAAGCGAAACAAATGCATACAATGTAACAACACATGCAGATGGTACTGCGACATATGGGCCTAGAGTAACAAAATAA

>AIS2002059

ATGAAGAGAATTAGTAAAGATATATGGGCAGTATTTAAATTACTGTATCAAAATAAAGGGCGTTTTAGCATTAATGCCTTACTATTGCAGTTAATCATGATTTTTATTAGTAGTACATACTTAATTTTACTATTTAATATGATGTTAAAAGTAGCTGGGCAAAGCCAACTTACGATTAACAATTGGATGGAAATCGTTAGTCATCCCGCCAGTGTGATACTTCTTATTATATTCATATTAAGTGTTGCCTTTCTGATTTATGTAGAGTTTTCATTGTTAGTTTATATGGTTTATGCCGGCTTTGATCGACAAATTATTACATTTAAATCCATTTTTAAAAATGCCTTTGTAAATGTGCGTAAACTCATAGGTGTACCAGTTATTTTCTTTGTTATTTATTTAATGTTAATGATACCCATTGCCAACCTAGGACTAAGTTCAGTATTAACAAAAAATATTTACATACCTAAATTTTTAACGGAAGAACTTATGAAAACGACGAAAGGTATAATCATTTACGGTACCTTTATGATTGCTGTATTTATATTAAACTTTAAATTAATATTTACGTTACCGTTAACGATTTTAAACCGCCAGTCGTTATTTAAAAATATGAGACTAAGTTGGCAAATTACGAAGCGAAATAAGTTTCGACTTGTTATAGAAATAGTTATATTGGAACTCATCATTGGTGCGATTTTAACATTAATTATTTCAGGAGCAACATATCTTGCTATTTGTGTAGATGAAGAAGGAGATAAGTTTTTAGTCTCATCAATTTTATTTGTTGTATTGAAAAGCGCATTGTTCTTCTATTATTTATTTACGAAATTATCATTAATCAGTGTGTTAGTACTGCACTTAAAACAAGAGAATGTATTAGACCAACCGGGCTTAGAATTTAAATATCCAAAACCGAAACGGAAGTCTAGGTTCTTTATAATTTCAATGGTGCTTGCAGTGACATGTTTTATCGGTTATAACATGTACTTACTTTACAATAATACTATCAATACAAATATCTCCATTATTGGCCATCGTGGTTTCGAAGATAAAGGGGTTGAAAATTCTATTCCGTCATTGAAAGCTGCTGCAAAAGCGAATGTCGAATACGTTGAGTTAGATACAATTATGACGAAAGATAAACAATTTGTTGTTAGTCATGATAACAATTTGAAACGTTTAACAGGTGTTAATAAAAACATTTCTGAATCTAATTTCAAAGATGTCGTCGGTTTGAAAATGCGTCAAAATGGACATGAAGCAAAACTTGTATCCTTAGACGAATTTATTGAAACGGCTAAACAATCAAATGTGAAGCTACTAGTTGAGTTAAAGCCACATGGTAAAGAACCAGCAGATTATACACAACGTGTTATTGATATTTTGAAAAAGCATGGTGTTGAACATCAATATCGTGTGATGTCTTTGGATTATGATGTGATGACTAAGTTGAAAAAAGAAGCGCCATATCTCAAGTGTGGTTATATCATTCCGTTGCAGTTTGGTCATTTTAAAGAAACATCATTAGATTTCTTTGTCATCGAAGATTTTTCTTATTCGCCAAGACTTGTTAATCAAGCGCACTTGGAAAATAAAGAAGTCTATACTTGGACTATTAACGGCGAAGAAGATTTAACGAAATACTTACAAACCAATGTTGATGGTATTATCACAGATGACCCAGCATTAGCTGATCAGATTAAAGAAGAAAAGAAAGACGAAACATACTTCGATCGTTCTATAAGAATTTTGTTTGAATAATATAAACAAAGACCTCTAAAGTTATTAAGATGATACCTTCAGAGGTCTTTTTAATGTTGCCATCTATGGGATAGGCAATCGTTTCATTCGTTTATATTCATATGACAAGTATTTGTATGGCAATTTGGCGTCACAAACACTTACATGATTTATTGGTGAATTATTAATTGTTTTGTGAATGCAAAGGGTTAGAAATTGAATTGTAAATACTTTCTAATCTTTGTTTCGCTTTAGTCATTTGATCCAAATTTTTAGTGCGTATAGCGGATTTTGCAATATAGTGCGCAGCTAAAATATCGCGTTTTTGAAACGCATCTAAATTTAGGTACGATAATTTATTTAAGTCAGTGTTTGCTATTAATTCATGTAATTGATCTACAAGCGCTTGATGTTGATACGTATGTGATGTAGTTTCAGGTTTGCTTGCTAATTTAATACCAGTCGTATCAAGGAGCGCCGCTTTAATACCAGCAACTAAATATGTTTTGATTTTCATTTGTGTTGTCATGCTTTGTTACTCCTTTGATGTACATTAATCAAAAAAATTATACACTATTGTATATTGCAAAGCTAATTAACTATAACAAAAAGATAGTTAATGCTTTGTTTATTCTAGTTAATATATAGTTAATGTCTTTTAATATTTTGTTTCTTTAATGTAGATTGGGCAATTACATTTTGGAGGAATTAAAAAATTATGAAAAAGCAAATAATTTCGCTAGGCGCATTAGCAGTTGCATCTAGCTTATTTACATGGGATAACAAAGCAGATGCGATAGTAACAAAGGATTATAGTAAAGAATCAAGAGTGAAAGAAGAGAGTAAATATGATTCGCCAATGTCAAATTGGTATTATTGGGGAAAGGTTAAATCCTTGGAGTCACAATTTGCAGATGCGATAGATATTATAGAAGATTATCAATATGGGGAAAAAGAGTATAAAGACGCCAAAGACAAATTGATGACTAGGGTATTAGGTGAAGACCAATACTTATTAAAGAAAAAAATAGATGAATATAAACAATATAGAGAAAGATATTTAAAAGCTGGTTTAAGCCCGGTGAAATTTTATGATTATAACCTTTATGATTTTACAATGAAAGAATATAATGATATCCATCACTCTCTAAAAGGTGCAGTGGAAGAGTTCTATAAAGAAGTTAAACATATTCAATCAAAGAATTCGGATTTACAAACTTATGACAAGAAAACTGAAGATGAAGAAACTGATAAAGTATATTCATTAGTTAGTGAAATTGATACTCTTGTTGTAACATATTATGGAGATAAGGATTATGGGGAGCACGCTAAAGAGTTGAGAGCTAAGCTAGATATTATTCTTGGAGAAGAAAAAAAGCCCAATAGAATAACTAATGAACGTATTAGAAAAGAAATGACCGATGATTTAAATTCTATTATCGATGACTTCTTTATGGAAACTGGACAAAACAGACCGGTTAAAATCACTAAATATAATCCAAATATTCATAGTCCTAAAGATAACAAAGAAAACTTCGATGCTTTAGTTAAAGAAACAAGAGAAGCAGTTGAAAAAGCAGATGATTCTTGGAAAAAGAAAACTGTCAAAAAATACGGAGAAACTGAAACAAAATCGCCAGTAGTAAAAGAAGAGAAGAAAGTTGAAGAACCTCAAGCACCTAAAGTTGATAACCAACAAGAAGTTAAAACTACTGCTGGTAAAGCTGAAGAAACAACACAACCAGTGGCACAGCCATTAGTAAAAATTCCACAAGGAACAATCTATGGTGAAACTGTAAAAGGTCCAGACTATCCAACTATGGAAAATAAAACGTTACAAGGTGTAATTGTTCAAGGTCCAGATTTCCCAACAATGGAACAAAGCGGCCCATCATTAAGCAATAATTATACAAACCCACCGTTAACGAACCCTATTTTAGAAGGTCTTGAAGGTAGCTCATCTAAACTTGAAATAAAACCACAAGGTACTGAATCAACGTTAAAAGGTACTCAAGGAGAATCAAGTGATATTGAAGTTAAACCTCAAGCAACTGAAACAACAGAAGCTTCTCAATATGGTCCGAGACCGCAATTTAACAAAACACCTAAATATGTTAAATATAGAGATGCTGGTACAGGTATCCGTGAATACAACGATGGAACATTTGGATATGAAGCGAGACCAAGATTCAATAAGCCATCAGAAACAAACGCATACAACGTAACGACAAATCAAGATGGCACAGTAACATATGGCGCTCGTCCAACACAAAACAAGCCAAGTAAAACGAACGCATATAACGTAACAACACATGCAAACGGCCAAGTATCATATGGCGCTCGCCCAACACAAAACAAGCCAAGCAAAACAAATGCATACAACGTAACAACACATGCAAATGGTCAAGTATCATATGGCGCTCGCCCGACACAAAACAAGCCAAGCAAAACAAATGCATATAACGTAACAACACATGCAAATGGTCAAGTATCATACGGAGCTCGCCCGACATACAAGAAGCCAAGCGAAACAAATGCATACAACGTAACAACACATGCAAATGGTCAAGTATCATATGGCGCTCGCCCGACACAAAACAAGCCAAGCGAAACAAACGCATATAATGTAACAACACATGCAGATGGTACTGCGACATATGGGCCTAGAGTAACAAAATAAGTTTGTAACTCTATCCAAAGACATACAAGTCAATACAAAACATTACGTATCTTTACAACAGTAATCATGCATTCTATGATGCTTCTAACTGAATTAAAGCATCGAACAATCGAAAGCATATTTCTAAATTATTTATTCATTATAGTATTAAACATAACATGACCTAATATATTACTAACCTATTAAAATAAACCACGCACATCTAAGTGATATACGACAATCACAGCAATAATAATTGCTTTAGAAAGTCGTGCCGAACTGGAACTTACAAGTCTAGTTCGAACACACACTGATGTGAGTGGTTTTCTTTATTTTAAACATGAACAATCAGATAAGTTACTAGCATTAGCAAATATTATTAAATCAAAGGGCTTCGATTCATAAAATTTAAAACAATGATTAAAATTAGACGTGTAAATGTTAAATTCTAAAACGGAAATAACCACCATCCCATTAAACCACTTTTTTGTTCAATCACTATATTTCACACAGCTTCATTAATAAAACGAAATTGCTTCAACCCGCTTCAACTTCAATCTGCTTCAACTTCAGCCTACTTCATTCAATAACCAAACGAATCCGCTTCATCCAAAATCAACCATTCTAACGCACATATTCAAATATAGCAGCTGCACCCATACCGACACCAATACACATCGTAACCATGCCGTAACGGCTATCGGGACGTCTACCCATTTCATTAAGTAAACGCGCGGTTAACATTGCGCCTGTAGCACCTAATGGATGACCTAAAGCAATAGCGCCACCATTCACATTCGTACGTGATATATCTAGACCTACTTCTTTAATAGATGCAATCGTTTGAGAAGCAAATGCTTCGTTCAATTCGATCAAATCAATGTCTTCAACAGATAGATTGCTGAGTGACAATACTTCAGGAATCGCATATGCAGGCCCAATACCCATAATTTTCGGGTCAACGCCTACTGCCTTATAACCAACGAATCGTGCAATAGGTGTCACGCCTAGTTCTTTCACTTTATCTCCAGACATTAAAACTACAAATCCTGCACCATCAGAAAGTGGGGCAGATGTTCCCGCAGTCACAGTGCCGTCAGCTTTAAATACTGTACGTAATTTGGCTAATGCGTCCATCGTGGTGTCAGGGCGTATAAATTCATCTTGGTCAAAGATATTTGTGTGTACTTTTGGTCCTGCGTTTGTATATTCAACTGAGTTTACTTGTATTGGGATAATTTCATCGTTGAACCGACCATCACGTTGTGCGTCATAGGCACGTTGATGACTTCTGACAGCATAAGCATCTTGATCTTCGCGTGATACGTCAAATTGGGATGCTACATTTTCAGCAGTTAAACCCATAGGATATGACGCACCTATATCATCATATTGTAAGGTTGGATTGTTTGTGGGCTCGTTGCCACCCATTGGTACGGCACTCATCAATTCAACGCCACCAGCTACAAGTATATCTCCTTGACCAGCCATAATTTGATTGGCTGCAATCGCGATGGTTTGTAATCCTGATGAGCAGTAGCGATTCACTGTTTGACCCGGTACCGTGTCAGATAATCCCGTACGCAATGCAATCGTTCGTGCAATGTTTTGTCCTTGTAATCCTTCTGGAAAAGCCGTACCAACAATGACATCTTCAATCATATTCTTATTGAATTTTCCGTCAATACGTTTCAATACGCCTTGTAATACTTTGGCTGCGACATCATCAGGTCTTTCGTGGAATAATGCGCCTTGCTTTGCTTTCGCTGCGGCTGAACGCCCATAAGCTACAATGTATGCTTCTTGCAT

>01093

ATGAAAAAGCAAATAATTTCGCTAGGCGCATTAGCAGTTGCATCTAGCTTATTTACATGGGATAACAAAGCAGATGCGATAGTAACAAAGGATTATAGTAAAGAATCAAGAGTGAAAGAAGAGAGTAAATATGATTCGCCAATGTCAAATTGGTATTATTGGGGAAAGGTTAAATCCTTGGAGTCACAATTTGCAGATGCGATAGATATTATAGAAGATTATCAATATGGGGAAAAAGAGTATAAAGACGCCAAAGACAAATTGATGACTAGGGTATTAGGTGAAGACCAATACTTATTAAAGAAAAAAATAGATGAATATAAACAATATAGAGAAAGATATTTAAAAGCTGGTTTAAGCCCGGTGAAATTTTATGATTATAACCTTTATGATTTTACAATGAAAGAATATAATGATATCCATCACTCTCTAAAAGGTGCAGTGGAAGAGTTCTATAAAGAAGTTAAACATATTCAATCAAAGAATTCGGATTTACAAACTTATGACAAGAAAACTGAAGATGAAGAAACTGATAAAGTATATTCATTAGTTAGTGAAATTGATACTCTTGTTGTAACATATTATGGAGATAAGGATTATGGGGAGCACGCTAAAGAGTTGAGAGCTAAGCTAGATATTATTCTTGGAGAAGAAAAAAAGCCCAATAGAATAACTAATGAACGTATTAGAAAAGAAATGACCGATGATTTAAATTCTATTATCGATGACTTCTTTATGGAAACTGGACAAAACAGACCGGTTAAAATCACTAAATATAATCCAAATATTCATAGTCCTAAAGATAACAAAGAAAACTTCGATGCTTTAGTTAAAGAAACAAGAGAAGCAGTTGAAAAAGCAGATGATTCTTGGAAAAAGAAAACTGTCAAAAAATACGGAGAAACTGAAACAAAATCGCCAGTAGTAAAAGAAGAGAAGAAAGTTGAAGAACCTCAAGCACCTAAAGTTGATAACCAACAAGAAGTTAAAACTACTGCTGGTAAAGCTGAAGAAACAACACAACCAGTGGCACAGCCATTAGTAAAAATTCCACAAGGAACAATCTATGGTGAAACTGTAAAAGGTCCAGACTATCCAACTATGGAAAATAAAACGTTACAAGGTGTAATTGTTCAAGGTCCAGATTTCCCAACAATGGAACAAAGCGGCCCATCATTAAGCAATAATTATACAAACCCACCGTTAACGAACCCTATTTTAGAAGGTCTTGAAGGTAGCTCATCTAAACTTGAAATAAAACCACAAGGTACTGAATCAACGTTAAAAGGTACTCAAGGAGAATCAAGTGATATTGAAGTTAAACCTCAAGCAACTGAAACAACAGAAGCTTCTCAATATGGTCCGAGACCGCAATTTAACAAAACACCTAAATATGTTAAATATAGAGATGCTGGTACAGGTATCCGTGAATACAACGATGGAACATTTGGATATGAAGCGAGACCAAGATTCAATAAGCCATCAGAAACAAACGCATACAACGTAACGACAAATCAAGATGGCACAGTAACATATGGCGCTCGTCCAACACAAAACAAGCCAAGTAAAACGAACGCATATAACGTAACAACACATGCAAACGGCCAAGTATCATATGGCGCTCGCCCAACACAAAACAAGCCAAGCAAAACAAATGCATACAACGTAACAACACATGCAAATGGTCAAGTATCATATGGCGCTCGCCCGACACAAAACAAGCCAAGCAAAACAAATGCATATAACGTAACAACACATGCAAATGGTCAAGTATCATACGGAGCTCGCCCGACATACAAGAAGCCAAGCGAAACAAATGCATACAACGTAACAACACATGCAAATGGTCAAGTATCATATGGCGCTCGCCCGACACAAAACAAGCCAAGCGAAACAAACGCATATAATGTAACAACACATGCAGATGGTACTGCGACATATGGGCCTAGAGTAACAAAATAA

>W12

ATGAAAAAGCAAATAATTTCGCTAGGCGCATTAGCAGTTGCATCTAGCTTATTTACATGGGATAACAAAGCAGATGCGATAGTGACAAAGGATTATAGCGAAGAATCTAGGGTGAATGAGAACAGTAAATATGGGACATTAATTTCAGACTGGTATTTAAGAGGGAGATTAACTAGTCTAGAATCTCAATTTATCAATGCATTGGATATTTTAGAGACATATCATTATGGCGAAAAAGAGTATAAAGATGCAAAAGATAAATTGATGACAAGAATTTTAGGGGAAGACCAATACCTTTTAGAAAGAAAAAAAGTGCAGTATGAGGAATACAAAAAATTATACCAAAAATATAAAGAAGAGAATCCAACCTCTAAAGTTAAAATGAAAACATTCCATCAATATACAATAGAAGATTTAACTATGAGGGAATATAATGAGTTAACAGAATCGTTAAGAAGTGCTGTAAAAGACTTTGAGAAAGATGTTGAAAGAATTGAAAATCAACATCATGATTTGAAACCATTTACTGATGAAATGGAAGAGAAGGCTACTTCTAGAGTTGATGATTTAGCAAATAAAGCATATAGTGTTTATTTTGCATTTGTTAGGGATACACAACATAAAACTGAGGCATTAGAGTTAAAAGCGAAAGTAGATTTAGTTTTAGGTGATGAGGATAAACCGCATCGTATTTCTAATGAAAGAATTGAAAAAGAAATGATAAAAGATTTAGAATCTATTATTGAAGATTTCTTTATAGAAACTGGTTTAAATAAGCCTGTTAATATTACGAGTTATGATAGTAGTAAACATCACTATAAAAATCACAGTGAAGGTTTTGAGGCTCTAGTTAAAGAAACAAGAGAAGCGGTCACAAACGCTAATGATTCTTGGAAAACTAAAGCTGTCAAAAAATACGGGGAAACTGAAATAAAATCGCCAGTAGTAAAAGAAGAGAAGAAAGTTGAAGAACCTCAATCACCTAAATTTGACAACCAACAAGAGGTTGAAACTACGGCTGGTAAAGCTGAAGAAACAACACAACCAGTTGCACAACCTCTAGTTAAGATTCCAGAAGGTACAATTCAAGGTGTACCTGTAGAAGGACCAAAATACCCAACGATGGAACAGCACACTATCTATGGTGAAATTGTAAAAGGTCCGGAATATCCAACGATGGAAAATAAAACGTTACAAGGTGAAATCGTTCAAGGTCCAGATTTTCTAACAATGGAACAAAACAGACCATCTTTAAGCGACAATTATACACAACCGACGACACCGAACCCTATTTTAAAAGGTATTGAAGGAACCTCATCTAAACTTGAAATAAAACCACAAGGTACTGAATCAACGTTGAAAGGTATTCAAGGAGAATCAAGTGATATTGAAGTTAAACCTCAAGCAACTGAAACAACAGAAGCTTCTCAATATGGTCCGAGACCGCAATTTAACAAAACACCTAAGTATGTTAAATATAGAGATGCTGGTACAGGTATCCGTGAATACAACGATGGAACATTTGGATATGAAGCGAGACCAAGATTCAATAAGCCATCAGAAACAAACGCATACAACGTAACGACAAATCAAGATGGCACAGTAACATATGGCGCTCGTCCAACACAAAACAAGGCAAGCAAAACAAACGCATATAACGTAACAACACATGCAAACGGTCAAGTATCATACGGAGCTCGTCCGACATACAAGAAGCCAAGCGAAACGAATGCATATAACGTAACAACACATGCAAACGGTCAAGTATCATATGGCGCTCGTCCGACACAAAACAAGGCAAGCGAAACAAATGCATACAACGTAACAACACATGCAAATGGTCAAGTATCATATGGCGCTCGTCCGACACAAAACAAGCCAAGCAAAACAAACGCATATAACGTAACAACACACGGAAATGGCCAAGTATCATACGGCGCCCGCCCGACATACAAGAAGCCAAGCGAAACAAATGCATACAATGTAACAACACATGCAGATGGTACTGCGACATATGGGCCTAGAGTAACAAAATAA

>91-2619

ATGAAAAAGCAAATAATTTCGCTAGGCGCATTAGCAGTTGCATCTAGCTTATTTACATGGGATAACAAAGCAGATGCGATAGTAACAAAGGATTATAGTGGGAAATCACAAGTTAATGCTGGGAGTAAAAATGGGAAACAAATTGCAGATGGATATTATTGGGGAATAATTGAAAATCTAGAAAACCAGTTTTACAATATTTTTCATTTACTGGATCAGCATAAATATGCAGAAAAAGAATATAAAGATGCAGTAGATAAATTAAAAACTAGAGTTTTAGAGGAAGACCAATACCTGCTAGAAAGAAAAAAAGAAAAATACGAAATTTATAAAGAACTATATAAAAAATACAAAAAAGAGAATCCTAATACTCAAGTTAAAATGAAAGCATTTGATAAATACGATCTTGGCGATTTAACTATGGAAGAATACAATGACTTATCAAAATTATTAACAAAAGCATTGGATAACTTTAAGTTAGAAGTAAAGAAAATTGAATCAGAGAATCCAGATTTAAAACCATATTCTGAAAGCGAAGAAAGAACAGCATATGGTAAAATAGATTCACTTGTTGATCAAGCATATAGTGTATATTTTGCCTACGTTACAGATGCACAACATAAAACAGAAGCATTAAATCTTAGGGCGAAAATTGATTTGATTTTAGGTGATGAAAAAGATCCAATTAGAGTTACGAATCAACGTACTGAAAAAGAAATGATTAAAGATTTAGAATCTATTATTGATGATTTCTTCATTGAAACCAAGTTGAATAGACCTAAACACATTACTAGGTATGATGGAACTAAACATGATTACCATAAACATAAAGATGGATTTGATGCTCTAGTTAAAGAAACAAGAGAAGCGGTTGCAAAGGCTGACGAATCTTGGAAAAATAAAACTGTCAAAAAATACGAGGAAACTGTAACAAAATCTCCAGTTGTAAAAGAAGAGAAGAAAGTTGAAGAACCTCAATCACCTAAATTTGATAACCAACAAGAGGTTAAAATTACAGTTGATAAAGCTGAAGAAACAACACAACCAGTGGCACAGCCATTAGTTAAAATTCCACAGGGCACAATTACAGGTGAAATTGTAAAAGGTCCGGAATATCCAACGATGGAAAATAAAACGTTACAAGGTGAAATCGTTCAAGGTCCAGATTTCCCAACAATGGAACAAAACAGACCATCTTTAAGCGATAATTATACTCAACCGACGACACCGAACCCTATTTTAGAAGGTCTTGAAGGTAGCTCATCTAAACTTGAAATAAAACCACAAGGTACTGAATCAACGTTAAAAGGTACTCAAGGAGAATCAAGTGATATTGAAGTTAAACCTCAAGCATCTGAAACAACAGAAGCATCACATTATCCAGCAAGACCTCAATTTAACAAAACACCTAAATATGTTAAATATAGAGATGCTGGTACAGGTATCCGTGAATACAACGATGGAACATTTGGATATGAAGCGAGACCAAGATTCAATAAGCCATCAGAAACAAACGCATACAACGTAACGACAAATCAAGATGGCACAGTAACATATGGCGCTCGCCCAACACAAAACAAACCAAGCAAAACAAATGCATACAACGTAACAACACATGCAAATCGTCAAGTATCATATGGCGCTCGCCCGACACAAAACAAGCCAAGCAAAACAAATGCATATAACGTAACAACACATGCAAATGGTCAAGTATCATACGGAGCTCGCCCGACACAAAACAAGCCAAGCAAAACAAATGCATATAACGTAACAACACACGCAAACGGTCAAGTGTCATACGGAGCTCGCCCGACATACAAGAAGCCAAGTAAAACAAATGCATACAATGTAACAACACATGCAGATGGTACTGCGACATATGGGCCTAGAGTAACAAAATAA

>WIS

ATGAAAAAGCAAATAATTTCGCTAGGCGCATTAGCAGTTGCATCTAGCTTATTTACATGGGATAACAAAGCAGATGCGATAGTAACAAAGGATTATAGTGGGAAATCACAAGTTAATGCTGGGAGTAAAAATGGGAAACAAATTGCAGATGGATATTATTGGGGAATAATTGAAAATCTAGAGAACCAGTTTTACAATATTTTTCATTTATTGGATCAGCATAAATATGCAGAAAAAGAATATAAAGATGCATTAGATAAATTAAAAACTAGAGTTTTAGAGGAAGACCAATACCTGCTAGAAAGAAAAAAAGAAAAATACGAAATTTATAAAGAACTATATAAAAAATACAAAAAAGAGAATCCTAATACTCAGGTTAAAATGAAAGCATTTGATAAATACGATCTTGGCGATTTAACTATGGAAGAATACAATGACTTATCAAAATTATTAACAAAAGCATTGGATAACTTTAAGTTAGAAGTAAAGAAAATTGAATCAGAGAATCCAGATTTAAGACCATATTCTGAAAGTGAAGAGAGAACAGCATATGGTAAAATAGATTCACTTGTTGATCAAGCATATAGTGTATATTTTGCCTACGTTACAGATGCTCAACATAAAACAGAAGCATTAAATCTTAGGGCAAAAATAGATTTGATTTTAGGTGATGAAAAAGATCCAATTAGAGTGACGAATCAACGTACTGAAAAAGAAATGATTAAAGATTTAGAATCTATTATTGATGATTTCTTCATTGAAACAAAGTTGAATAGACCTCAACACATTACTAGATATGATGGAACTAAACATGATTACCATAAACATAAAGATGGATTTGATGCTTTAGTTAAAGAAACAAGAGAAGCGGTTTCTAAGGCTGACGAATCTTGGAAAACTAAAACTGTCAAAAAATACGGGGAAACTGAAACAAAATATCCTGTTGTAAAAGAAGAGAAGAAAGTTGAAGAACCTCAATCACCTAAAGTTTCTGAAAAAGTGGATGTTCAGGAAACGGTTGGTACAACTGAAGAAGCACCATTACCAATTGCGCAACCACTAGTTAAATTACCACAAATTGGGACTCAAGGCGAAATTGTAAAAGGTCCCGACTATCCAACTATGGAAAATAAAACGTTACAAGGTGTAATTGTTCAAGGTCCAGATTTCCCAACAATGGAACAAAACAGACCATCTTTAAGTGACAATTATACACAACCATCTGTGACTTTACCGTCAATTACAGGTGAAAGTACACCAACGAACCCTATTTTAAAAGGTATTGAAGGAAACTCATCTAAACTTGAAATAAAACCACAAGGTACTGAATCAACGTTGAAAGGTATTCAAGGAGAATCAAGTGATATTGAAGTTAAACCTCAAGCAACTGAAACAACAGAAGCATCACATTATCCAGCGAGACCGCAATTTAACAAAACACCTAAATATGTGAAATATAGAGATGCTGGTACAGGTATTCGTGAATACAACGATGGAACTTTTGGATATGAAGCGAGACCAAGATTCAACAAGCCATCAGAAACAAACGCATACAACGTAACGACAAATCAAGATGGCACAGTATCATATGGGGCTCGCCCAACACAAAACAAGCCAAGCAAAACAAATGCATATAACGTAACAACACATGCAAACGGCCAAGTATCATATGGCGCTCGCCCGACATACAACAAGCCAAGTGAAACAAATGCATACAACGTAACGACAAATCGAGATGGCACAGTATCATATGGCGCTCGCCCGACACAAAACAAGCCAAGCGAAACGAATGCATATAACGTAACAACACACGGAAATGGCCAAGTATCATATGGCGCTCGTCCGACACAAAAGAAGCCAAGCAAAACAAATGCATATAACGTAACAACACATGCAAACGGCCAAGTATCATATGGCGCTCGTCCGACATACAACAAGCCAAGTAAAACAAATGCATACAATGTAACAACACATGCAGATGGTACTGCGACATATGGTCCTAGAGTAACAAAATAA

>NVAU02081

# ATGAAGAGAATTAGTAAAGATATATGGGCAGTATTTAAATTACTGTATCAAAATAAAGGGCGTTTTAGCATTAATGCCTTACTATTGCAGTTAATCATGATTTTTATTAGTAGTACATACTTAATTTTACTATTTAATATGATGTTAAAAGTAGCTGGGCAAAGCCAACTTACGATTAACAATTGGACGGAAATCGTTAGTCATCCCGCCAGTGTGATACTTCTTATTATATTCATATTAAGTGTTGCCTTTCTGATTTATGTAGAGTTTTCATTGTTAGTTTATATGGTTTATGCCGGCTTTGATCGACAAATTATTACATTTAAATCCATTTTTAAAAATGCCTTTGTAAATGTGCGTAAACTCATAGGTGTACCAGTTATTTTCTTTGTTATTTATTTAATGTTAATGATACCCATTGCCAACCTAGGACTAAGTTCAGTATTAACAAAAAATATTTACATACCTAAATTTTTAACGGAAGAACTTATGAAAACGACGAAAGGTATAATCATTTACGGTACCTTTATGATTGCTGTATTTATATTAAACTTTAAATTAATATTTACGTTACCGTTAACGATTTTAAACCGCCAGTCGTTATTTAAAAATATGAGACTAAGTTGGCAAATTACGAAGCGAAATAAGTTTCGACTTGTTATAGAAATAGTTATATTGGAACTCATCATTGGTGCGATTTTAACATTAATTATTTCAGGAGCAACATATCTTGCTATTTGTGTAGATGAAGAAGGAGATAAGTTTTTAGTCTCATCAATTTTATTTGTTGTATTGAAAAGCGCATTGTTCTTCTATTATTTATTTACGAAATTATCATTAATCAGTGTGTTAGTACTGCACTTAAAACAAGAGAATGTATTAGACCAACCGGGCTTAGAATTTAAATATCCAAAACCGAAACGGAAGTCTAGGTTCTTTATAATTTCAATGGTGCTTGCAGTGACATGTTTTATCGGTTATAACATGTACTTACTTTACAATAATACTATCAATACAAATATCTCCATTATTGGCCATCGTGGTTTCGAAGATAAAGGGGTTGAAAATTCTATTCCGTCATTGAAAGCTGCTGCAAAAGCGAATGTCGAATACGTTGAGTTAGATACAATTATGACGAAAGATAAACAATTTGTTGTTAGTCATGATAACAATTTGAAACGTTTAACAGGTGTTAATAAAAACATTTCTGAATCTAATTTCAAAGATGTCGTCGGTTTGAAAATGCGTCAAAATGGACATGAAGCAAAACTTGTATCCTTAGACGAATTTATTGAAACGGCTAAACAATCAAATGTGAAGCTACTAGTTGAGTTAAAGCCACATGGTAAAGAACCAGCAGATTATACACAACGTGTTATTGATATTTTGAAAAAGCATGGTGTTGAACATCAATATCGTGTGATGTCTTTGGATTATGATGTGATGACTAAGTTGAAAAAAGAAGCGCCATATCTCAAGTGTGGTTATATCATTCCGTTGCAGTTTGGTCATTTTAAAGAAACATCATTAGATTTCTTTGTCATCGAAGATTTTTCTTATTCGCCAAGACTTGTTAATCAAGCGCACTTGGAAAATAAAGAAGTCTATACTTGGACTATTAACGGCGAAGAAGATTTAACGAAATACTTACAAACCAATGTTGATGGTATTATCACAGATGACCCAGCATTAGCTGATCAGATTAAAGAAGAAAAGAAAGACGAAACATACTTCGATCGTTCTATAAGAATTTTGTTTGAATAATATAAACAAAGACCTCTAAAGTTATCAAGATGATACCTTCAGAGGTCTTTTTAATGTTGCCATCTATGGGATAGGCAATCGTTTCATTCGTTTATATTCATATGACAAGTATTTGTATGGCAATTTGGCGTCACAAACACTTACATGATTTATTGGTGAATTATTAATTGTTTTGTGAATGCAAAGGGTTAGAAATTGAAACGTAAATACTTTCTAATCTATGTTTCGCTTTAGTCATTTGATCCAAATTTTTAGTGCGTATAGCTGATTTAGCAATATAGTGCGCAGCTAAAATGTCGCGTTTTTGATACGCATCTAAATTTAGGTACGATAATTTATTAAAGTCAGTGTTTGCTATTAATTCATGTAATTGATCTACAAGCGCTTGATGTTGATACGTATGTGATGTAGTTTCAGATTTGCTTGCTAATTTAATACCAGTCGTATCAAGGAGCGCCGCTTTAATACCAGCAACTAAATATGTTTTGATTTTCATTTGTGTTGTCATGCTTTGTTACTCCTTTGATGTACATTAATCAAAAAAATTATACACTATTGTATATTGCAAAGCTAATTAACTATAACAAAAAGATAGTTAATGCTTTGTTTATTCTAGTTAATATATAGTTAATGTCTTTTAATATTTTGTTTCTTTAATGTAGATTGGGCAATTACATTTTGGAGGAATTAAAAAATTATGAAAAAGCAAATAATTTCGCTAGGCGCATTAGCAGTTGCATCTAGCTTATTTACATGGGATAACAAAGCAGATGCGATAGTAACAAAGGATTATAGCAAAGAATCTAGGGTGAATGAGAACAGTAAATATGATACACCAATTTCAGACACTTATTACTGGGGAGTAATAAAAAATCTAGAGTCACAATTTGCTGAAGCAATTGATTTATTAGAGGATTATCAATATGGAGAAAAAGAATATAAAGATGCTAAAGACAAACTGATGACTAGGCTTTTAGGTGAAGATCAATATTTATTGAAGAAAAAAATCGATGAGTATGAAGTATACAAAGAATCGTTTAAAAAATTTAAAGAAAAAAATCCTAATGATAATTCGAAAATGAGTTCTTTTTATAACTATAATTTATATAATTTTACTATGAAAGAATATAATGATATAAGACATTCTTTAAAAGAAGCGATAGATCAGTTCCGTAAAGATGTAGATGATATACATTCGAAAAAGGTAGACTTAAAAACATACAGTAAAGAAGCCGAAGAAAAAGCTACAGATGAAGTTTATAGCCTTGTCTGTGAAGTTGACACACTTTTTGCATCATACTATGGTCATGATAAATATGATCAAAATGCTAAAGAATTACGTGCTAAATTAGATCTGATACTTGGAGATAAAGATAACCCACATAAAATTACTAATGAGCGTATTAAAAAAGAAATGATGGACGATTTAAATTCTATTATTGATGATTTCTTTATGGAAACTAATCAAAATAGACCATCAAACATAACTAAATTTAATCCAGATATTCATGGGTATAAAGAGCATAGAGAAAGCTTCGATGCTCTAGTTAAAGAAACAAGAGAAGCGGTTGCAAAGGCTGACGAATCTTGGAAAACTAAAACTGTAAAAAATTACGGTGAATCTGAAACAAAAGCACATGTTGTAAAAGAAGAGAAGAAAGTTGAAGAACCTCAATTACCTAAAGTTGGAAACCAGCAAGAGGATAAAACTACAGTTGATAAAGCTGAAGAAACGACACAACCAGTGGCACAGCCATTAATAAAAATTCCACAAGGAACAATCTATGGTGAAATTGTGAAAGATCCTGACTATCTAACGATGGAAAATAAAATGTTACAAGGTGAAATAGTTCAAGGTCCAGATTTTCTAACAATGGAACAAAACAGACCATCTTTAAGCGGTAATTATACACAACCATCTGTGACTTTACCGTCAATTACAGGTGAAAGTACACCAACGAACCCTATTTTAGAAGGTCTTGAAGGTAGCTCATCTAAACTTGAAATAAAACCACAAGGTACTGAATCAACGTTGAAAGGTATTCAAGGAGAATCAAGTGATATTGAGGTTAAACCTCAAGCATCTGAAACAACAGAAGCATCACATTATCCAGCGAGACCTCAATTTAACAAAACACCTAAATATGTTAAATATAGAGATGCTGGTACAGGTATCCGTGAATACAACGATGGAACATTTGGATATGAAGCGAGACCAAGATTCAATAAGCCATCAGAAACAAATGCATACAACGTAACAACACATGGAAACGGCCAAGTATCATATGGCGCTCGCCCAACACAAAACAAGCCAAGCAAAACAAATGCATACAACGTAACAACACATGCAAATGGTCAAGTATCATATGGCGCTCGCCCGACACAAAACAAGCCAAGCAAAACAAATGCATATAACGTAACAACACATGCAAATGGTCAAGTATCATACGGAGCTCGCCCGACATACAAGAAGCCAAGCGAAACAAATGCATACAACGTAACAACACATGCAAATGGTCAAGTATCATATGGCGCTCGCCCGACACAAAAAAAGCCAAGCGAAACAAACGCATATAACGTAACAACACATGCAGATGGTACTGCGACATATGGGCCTAGAGTAACAAAATAAGTTTGTAACTCTATCCAAAGACATACAGTCAATACAAAACATTACGTATCTTTACAACAGTAATCATGCATTCTATGATGCTTCTAACTGAATTAAAGCATCGAACAATCGGAAGCATATTTCTAAATTATTTATTCATTATAGTCTTAAACATAACATGACCTAATATATTACTAACCTATTAAAATAAACCACGCACATCTAAGTGATATACGACAATCACAGCAATAATAATTGCTTTAGAAAGTCGTGCCGAACTGGAACTTACAAGTCTAGTTCGAACACACACTGATGTGAGTGGTTTTCTTTATTTTAAACATGAACAATCAGATAAGTTACTAGCATTAGCAAATATTATTAAATCAAAGGGCTTCGATTCATAAAATTTAAAACAATGATTAGAATTAGACGTGTAAATGTTAAATTCTAAAACGGAAATAACCACCATCCCATTAAACCACTTTTTTGTTCAATCACTATATTTCACACAGCTTCATTAATAAAACGAAATTGCTTCAACCCGCTTCAACTTCAATCTGCTTCAACTTCAGCCTACTTCATTCAATAACAAAACGAATCCGCTTCATCCAAAATCAACCATTCTAACGCACATATTCAAATATAGCAGCTGCACCCATACCGACACCAATACACATCGTAACCATGCCGTAACGGCTATCGGGACGTCTACCCATTTCATTAAGTAAACGCGCGGTTAACATTGCGCCTGTAGCACCTAATGGATGACCTAAAGCAATAGCGCCACCATTCACATTTGTACGTGATATATCTAGACCTACTTCTTTAATAGATGCAATCGTTTGAGAAGCAAATGCTTCGTTCAATTCGATCAAATCAATGTCTTCAACAGATAGATTGCTGAGTGACAATACTTCAGGAATCGCATATGCAGGCCCAATACCCATAATTTTCGGGTCAACGCCTACTGCCTTATAACCAACGAATCGTGCAATAGGTGTCACGCCGAGTTCTTTCACTTTATCTCCAGACATTAAAACTACAAATCCTGCACCATCAGAAAGTGGGGCAGATGTTCCTGCAGTCACAGTGCCGTCAGCTTTAAATACTGTACGTAATTTGGCTAATGCCTCCATCGTGGTGTCAGGGCGTATAAATTCATCTTGGTCAAAGATATTTGTGTGTACTTTTGGTCCTGCGTTTGTATATTCAACTGAGTTTACTTGTATTGGAATAATTTCATCTTTGAACCGACCATCACGTTGTGCGTCATAGGCACGTTGATGACTTCTGACAGCATAAGCATCTTGATCTTCGCGTGATACGTCAAATTGGGATGCTACATTTTCAGCAGTTAAACCCATAGGATATGACGCACCTATATCATCATATTGTAAGGTAGGATTGTTTGTGGGCTCGTTGCCACCCATTGGTACGGCACTCATCAATTCAACGCCACCAGCTACAAGTATATCTCCTTGACCAGCCATAATTTGATTGGCTGCAATCGCGATGGTTTGTAATCCTGATGAGCAGTAGCGATTCACTGTTTGACCCGGTACCGTGTCAGATAATCCCGTACGCAATGCAATCGTTCGTGCAATGTTTTGTCCTTGTAATCCTTCTGGAAAAGCCGTACCAACAATGACATCTTCAATCATATTCTTATTGAATTTTCCGTCAATACGTTTCAATACGCCTTGTAATACTTTGGCTGCGACATCATCAGGTCTTTCGTGGAATAATGCGCCTTGCTTTGCTTTCGCTGCGGCTGAACGCCCATAAGCTACAATGTATGCTTCTTGCAT

# Supplementary Data

# Comparison among the *coa* gene sequence (nucleotides and aminoacids) in the 4 GTBN samples and MSSA_129, Stp58 and Stp25 strains.

**A)**

GTBN_1-4-5-12 ATGAAAAAGCAAATAATTTCGCTAGGCGCATTAGCAGTTGCATCTAGCTTATTTACATGG

MSSA_129 ATGAAAAAGCAAATAATTTCGCTAGGCGCATTAGCAGTTGCATCTAGCTTATTTACATGG

************************************************************

GTBN_1-4-5-12 GATAACAAAGCAGATGCGATAGTAACAAAGGATTATAGTAAAGAATCAAGAGTGAAAGAG

MSSA_129 GATAACAAAGCAGATGCGATAGTAACAAAGGATTATAGTAAAGAATCAAGAGTGAAAGAG

************************************************************

GTBN_1-4-5-12 AACAGTAAATATGATTCGCCAATGTCAAATTGGTATTATTGGGGAAAGGTTAAATCCTTG

MSSA_129 AACAGTAAATATGATTCGCCAATGTCAAATTGGTATTATTGGGGAAAGGTTAAATCCTTG

************************************************************

GTBN_1-4-5-12 GAGTCACAATTTGCAGATGCAATAGATATTATAGAAGATTATCAATATGGTGAAAAAGAA

MSSA_129 GAGTCACAATTTGCAGATGCAATAGATATTATAGAAGATTATCAATATGGTGAAAAAGAA

************************************************************

GTBN_1-4-5-12 TATAAAGATGCAAAAGATAAACTAATGACTAGAATACTAGGTGAGGACCAATACTTATTA

MSSA_129 TATAAAGATGCAAAAGATAAACTAATGACTAGAATACTAGGTGAGGACCAATACTTATTA

************************************************************

GTBN_1-4-5-12 AAGAAAAAAATAGAAGAATATAAACAATATAGAGAAAGATATTTAAAAGCTGGATTAAGT

MSSA_129 AAGAAAAAAATAGAAGAATATAAACAATATAGAGAAAGATATTTAAAAGCTGGATTAAGT

************************************************************

GTBN_1-4-5-12 CCTGTGAAATTTTATGATTACAATCTTTATGATTTTACAATGAAAGAATATAATGATATC

MSSA_129 CCTGTGAAATTTTATGATTACAATCTTTATGATTTTACAATGAAAGAATATAATGATATC

************************************************************

GTBN_1-4-5-12 CATCAGTCTTTAAAAGATGCAGTAGAAGAGTTCTATCAAGAAGTTAAACATATTCAATCA

MSSA_129 CATCAGTCTTTAAAAGATGCAGTAGAAGAGTTCTATCAAGAAGTTAAACATATTCAATCA

************************************************************

GTBN_1-4-5-12 AAGAATTCGGATTTACAAACTTATGATAAGAAAACTGAAGATAAAGAAACTGATAATGTA

MSSA_129 AAGAATTCGGATTTACAAACTTATGATAAGAAAACTGAAGATAAAGAAACTGATAATGTA

************************************************************

GTBN_1-4-5-12 TACTCTTTAGTTAGTGAAATTGATACTATTGTTGCAACATATTATGGAGATAAAAATCAT

MSSA_129 TACTCTTTAGTTAGTGAAATTGATACTATTGTTGCAACATATTATGGAGATAAAAATCAT

************************************************************

GTBN_1-4-5-12 GGAGAGCATGCTAAAGAGTTGAGAGCTAAGCTAGATATTATTCTTGGAGAAGAAAAAAGC

MSSA_129 GGAGAGCATGCTAAAGAGTTGAGAGCTAAGCTAGATATTATTCTTGGAGAAGAAAAAAGC

************************************************************

GTBN_1-4-5-12 CAAATAGAATAACTAATGAACGTATTAGAAAAGAAATGACTGATGATTTGAATTCTATTA

MSSA_129 CAAATAGAATAACTAATGAACGTATTAGAAAAGAAATGACTGATGATTTGAATTCTATTA

************************************************************

GTBN_1-4-5-12 TCGATGACTTCTTTATGGAAACTGGGCAAAACAGACCGGTTAAAATCACTAAATATAATC

MSSA_129 TCGATGACTTCTTTATGGAAACTGGGCAAAACAGACCGGTTAAAATCACTAAATATAATC

************************************************************

GTBN_1-4-5-12 CAAATATTCATAGCCCTAAAGATAACAAAGAAAGCTTCGATAAATTAGTTGAAGAAACGA

MSSA_129 CAAATATTCATAGCCCTAAAGATAACAAAGAAAGCTTCGATAAATTAGTTGAAGAAACGA

************************************************************

GTBN_1-4-5-12 AAAAAGCAGTTAAAGAAGCAGATGAGTCTTGGAAAACTAAAACTGTCAAAACATACGGTG

MSSA_129 AAAAAGCAGTTAAAGAAGCAGATGAGTCTTGGAAAACTAAAACTGTCAAAACATACGGTG

************************************************************

GTBN_1-4-5-12 AAACTGAAACAAAAGCACATGTTGTAAAAGAAGAGAAGAAAGTTGAAGAACCTCAATTAC

MSSA_129 AAACTGAAACAAAAGCACATGTTGTAAAAGAAGAGAAGAAAGTTGAAGAACCTCAATTAC

************************************************************

GTBN_1-4-5-12 CTAAAGTTGGAAACCAGCAAGAGGATAAAACTACAGTTGGTACAACTGAAAAAGCACCAT

MSSA_129 CTAAAGTTGGAAACCAGCAAGAGGATAAAACTACAGTTGGTACAACTGAAAAAGCACCAT

************************************************************

GTBN_1-4-5-12 TACCAATTGCGCAACCACTAGTTAAATTACCACAAATTGGGACTCAAGGTAAAATTGTAG

MSSA_129 TACCAATTGCGCAACCACTAGTTAAATTACCACAAATTGGGACTCAAGGTAAAATTGTAG

************************************************************

GTBN_1-4-5-12 AAGGGCCAAAATACCCAACGATGGAACAGCACACAATCTATGGTGAAATTGTAAATGGTC

MSSA_129 AAGGGCCAAAATACCCAACGATGGAACAGCACACAATCTATGGTGAAATTGTAAATGGTC

************************************************************

GTBN_1-4-5-12 CCGACTATCTAACGATGGAAAATAAAACGTTACAAGGTGAAATCGTTCAAGGTCCTGATT

MSSA_129 CCGACTATCTAACGATGGAAAATAAAACGTTACAAGGTGAAATCGTTCAAGGTCCTGATT

************************************************************

GTBN_1-4-5-12 TCCCAACAATGGAACAAAACAGACCATCTTTAAGCGATAATTATACTCAACCGACGACAC

MSSA_129 TCCCAACAATGGAACAAAACAGACCATCTTTAAGCGATAATTATACTCAACCGACGACAC

************************************************************

GTBN_1-4-5-12 CGAACCCTATTTTAGAAGGTCTTGAAGGTAGCTCATCTAAACTCGAAATAAAACCACAAG

MSSA_129 CGAACCCTATTTTAGAAGGTCTTGAAGGTAGCTCATCTAAACTCGAAATAAAACCACAAG

************************************************************

GTBN_1-4-5-12 GTACTGAATCAACGTTGAAAGGTATTCAAGGAGAATCAAGTGATATTGAAGTTAAACCTC

MSSA_129 GTACTGAATCAACGTTGAAAGGTATTCAAGGAGAATCAAGTGATATTGAAGTTAAACCTC

************************************************************

GTBN_1-4-5-12 AAGCAACTGAAACAACAGAAGCATCACATTATCCAGCGAGACCGCAATTTAACAAAACAC

MSSA_129 AAGCAACTGAAACAACAGAAGCATCACATTATCCAGCGAGACCGCAATTTAACAAAACAC

************************************************************

GTBN_1-4-5-12 CTAAGTATGTGAAATATAGAGATGCTGGTACAGGTATTCGTGAATACAACGATGGAACAT

MSSA_129 CTAAGTATGTGAAATATAGAGATGCTGGTACAGGTATTCGTGAATACAACGATGGAACAT

************************************************************

GTBN_1-4-5-12 TTGGATATGAAGCGAGACCAAGATTCAACAAGCCAAGTGAAACAAATGCATACAACGTAA

MSSA_129 TTGGATATGAAGCGAGACCAAGATTCAACAAGCCAAGTGAAACAAATGCATACAACGTAA

************************************************************

GTBN_1-4-5-12 CGACAAATCAAGATGGCACAGTATCATACGGCGCCCGCCCGACACAAAACAAGCCAAGCG

MSSA_129 CGACAAATCAAGATGGCACAGTATCATACGGCGCCCGCCCGACACAAAACAAGCCAAGCG

************************************************************

GTBN_1-4-5-12 AAACGAATGCATATAACGTAACAGCACACGGAAATGGTCAAGTGTCATACGGCGCTCGTC

MSSA_129 AAACGAATGCATATAACGTAACAGCACACGGAAATGGTCAAGTGTCATACGGCGCTCGTC

************************************************************

GTBN_1-4-5-12 CGACATACAAGAAGCCAAGCGAAACAAATGCATATAACGTAACAACACATGCAAATGGTC

MSSA_129 CGACATACAAGAAGCCAAGCGAAACAAATGCATATAACGTAACAACACATGCAAATGGTC

************************************************************

GTBN_1-4-5-12 AAGTATCATACGGAGCTCGCCCAACACAAAACAAGCCAAGCGAAACAAACGCATATAACG

MSSA_129 AAGTATCATACGGAGCTCGCCCAACACAAAACAAGCCAAGCGAAACAAACGCATATAACG

************************************************************

GTBN_1-4-5-12 TAACAACACATGCAAACGGTCAAGTGTCATACGG**A**GCTCGCCCGACACAAAACAAGCCAA

MSSA_129 TAACAACACATGCAAACGGTCAAGTGTCATACGGCGCTCGCCCGACACAAAACAAGCCAA

**********************************.*************************

GTBN_1-4-5-12 GCAAAACAAACGCATATAACGTAACAACACACGGAAATGGCACAGTATCATATGGCGCTC

MSSA_129 GCAAAACAAACGCATATAACGTAACAACACACGGAAATGGCACAGTATCATATGGCGCTC

************************************************************

GTBN_1-4-5-12 GCCCGACACAAAACAAGCCAAGTAAAACAAATGCATATAACGTAACAACACATGCAGATG

MSSA_129 GCCCGACACAAAACAAGCCAAGTAAAACAAATGCATATAACGTAACAACACATGCAGATG

************************************************************

GTBN_1-4-5-12 GTACTGCGACATATGGTCCTAGAGTAACAAAATAA

MSSA_129 GTACTGCGACATATGGTCCTAGAGTAACAAAATAA

***********************************

**B)**

GTBN_1-4-5-12 ATGAAAAAGCAAATAATTTCGCTAGGCGCATTAGCAGTTGCATCTAGCTTATTTACATGG

Stp25 ATGAAAAAGCAAATAATTTCGCTAGGCGCATTAGCAGTTGCATCTAGCTTATTTACATGG

Stp58 ATGAAAAAGCAAATAATTTCGCTAGGCGCATTAGCAGTTGCATCTAGCTTATTTACATGG

************************************************************

GTBN_1-4-5-12 GATAACAAAGCAGATGCGATAGTAACAAAGGATTATAGTAAAGAATCAAGAGTGAAAGAG

Stp25 GATAACAAAGCAGATGCGATAGTAACAAAGGATTATAGTAAAGAATCAAGAGTGAATGAG

Stp58 GATAACAAAGCAGATGCGATAGTAACAAAGGATTATAGTAAAGAATCAAGAGTGAATGAG

********************************************************:***

GTBN_1-4-5-12 AACAGTAAATATGATTCGCCAATGTCAAATTGGTATTATTGGGGAAAGGTTAAATCCTTG

Stp25 AACAGTAAATATGATTCGCCAATGTCAAATTGGTATTATTGGGGAAAGGTTAAATCCTTG

Stp58 AACAGTAAATATGATTCGCCAATGTCAAATTGGTATTATTGGGGAAAGGTTAAATCCTTG

************************************************************

GTBN_1-4-5-12 GAGTCACAATTTGCAGATGCAATAGATATTATAGAAGATTATCAATATGGTGAAAAAGAA

Stp25 GAGTCACAATTTGCAGATGCAATAGATATTATAGAAGATTATCAATATGGTGAAAAAGAA

Stp58 GAGTCACAATTTGCAGATGCAATAGATATTATAGAAGATTATCAATATGGTGAAAAAGAA

************************************************************

GTBN_1-4-5-12 TATAAAGATGCAAAAGATAAACTAATGACTAGAATACTAGGTGAGGACCAATACTTATTA

Stp25 TATAAAGATGCAAAAGATAAACTAATGACTAGAATACTAGGTGAGGACCAATACTTATTA

Stp58 TATAAAGATGCAAAAGATAAACTAATGACTAGAATACTAGGTGAGGACCAATACTTATTA

************************************************************

GTBN_1-4-5-12 AAGAAAAAAATAGAAGAATATAAACAATATAGAGAAAGATATTTAAAAGCTGGATTAAGT

Stp25 AAGAAAAAAATAGAAGAATATAAACAATATAGAGAAAGATATTTAAAAGCTGGATTAAGT

Stp58 AAGAAAAAAATAGAAGAATATAAACAATATAGAGAAAGATATTTAAAAGCTGGATTAAGT

************************************************************

GTBN_1-4-5-12 CCTGTGAAATTTTATGATTACAATCTTTATGATTTTACAATGAAAGAATATAATGATATC

Stp25 CCTGTGAAATTTTATGATTACAATCTTTATGATTTTACAATGAAAGAATATAATGATATC

Stp58 CCTGTGAAATTTTATGATTACAATCTTTATGATTTTACAATGAAAGAATATAATGATATC

************************************************************

GTBN_1-4-5-12 CATCAGTCTTTAAAAGATGCAGTAGAAGAGTTCTATCAAGAAGTTAAACATATTCAATCA

Stp25 CATCAGTCTTTAAAAGATGCAGTAGAAGAGTTCTATCAAGAAGTTAAACATATTCAATCA

Stp58 CATCAGTCTTTAAAAGATGCAGTAGAAGAGTTCTATCAAGAAGTTAAACATATTCAATCA

************************************************************

GTBN_1-4-5-12 AAGAATTCGGATTTACAAACTTATGATAAGAAAACTGAAGATAAAGAAACTGATAATGTA

Stp25 AAGAATTCGGATTTACAAACTTATGATAAGAAAACTGAAGATAAAGAAACTGATAATGTA

Stp58 AAGAATTCGGATTTACAAACTTATGATAAGAAAACTGAAGATAAAGAAACTGATAATGTA

************************************************************

GTBN_1-4-5-12 TACTCTTTAGTTAGTGAAATTGATACTATTGTTGCAACATATTATGGAGATAAAAATCAT

Stp25 TACTCTTTAGTTAGTGAAATTGATACTATTGTTGCAACATATTATGGAGATAAAAATCAT

Stp58 TACTCTTTAGTTAGTGAAATTGATACTATTGTTGCAACATATTATGGAGATAAAAATCAT

************************************************************

GTBN_1-4-5-12 GGAGAGCATGCTAAAGAGTTGAGAGCTAAGCTAGATATTATTCTTGGAGAAGAAAAAA-G

Stp25 GGAGAGCATGCTAAAGAGTTGAGAGCTAAGCTAGATATTATTCTTGGAGAAGAAAAAA**A**G

Stp58 GGAGAGCATGCTAAAGAGTTGAGAGCTAAGCTAGATATTATTCTTGGAGAAGAAAAAA**A**G

********************************************************** *

GTBN_1-4-5-12 CCAAATAGAATAACTAATGAACGTATTAGAAAAGAAATGACTGATGATTTGAATTCTATT

Stp25 CCAAATAGAATAACTAATGAACGTATTAGAAAAGAAATGACTGATGATTTGAATTCTATT

Stp58 CCAAATAGAATAACTAATGAACGTATTAGAAAAGAAATGACTGATGATTTGAATTCTATT

************************************************************

GTBN_1-4-5-12 ATCGATGACTTCTTTATGGAAACTGGGCAAAACAGACCGGTTAAAATCACTAAATATAAT

Stp25 ATCGATGACTTCTTTATGGAAACTGGGCAAAACAGACCGGTTAAAATCACTAAATATAAT

Stp58 ATCGATGACTTCTTTATGGAAACTGGGCAAAACAGACCGGTTAAAATCACTAAATATAAT

************************************************************

GTBN_1-4-5-12 CCAAATATTCATAGCCCTAAAGATAACAAAGAAAGCTTCGATAAATTAGTTGAAGAAACG

Stp25 CCAAATATTCATAGCCCTAAAGATAACAAAGAAAGCTTCGATAAATTAGTTGAAGAAACG

Stp58 CCAAATATTCATAGCCCTAAAGATAACAAAGAAAGCTTCGATAAATTAGTTGAAGAAACG

************************************************************

GTBN_1-4-5-12 AAAAAAGCAGTTAAAGAAGCAGATGAGTCTTGGAAAACTAAAACTGTCAAAACATACGGT

Stp25 AAAAAAGCAGTTAAAGAAGCAGATGAGTCTTGGAAAACTAAAACTGTCAAAACATACGGT

Stp58 AAAAAAGCAGTTAAAGAAGCAGATGAGTCTTGGAAAACTAAAACTGTCAAAACATACGGT

************************************************************

GTBN_1-4-5-12 GAAACTGAAACAAAAGCACATGTTGTAAAAGAAGAGAAGAAAGTTGAAGAACCTCAATTA

Stp25 GAAACTGAAACAAAAGCACATGTTGTAAAAGAAGAGAAGAAAGTTGAAGAACCTCAATTA

Stp58 GAAACTGAAACAAAAGCACATGTTGTAAAAGAAGAGAAGAAAGTTGAAGAACCTCAATTA

************************************************************

GTBN_1-4-5-12 CCTAAAGTTGGAAACCAGCAAGAGGATAAAACTACAGTTGGTACAACTGAAAAAGCACCA

Stp25 CCTAAAGTTGGAAACCAGCAAGAGGATAAAACTACAGTTGGTACAACTGAAAAAGCACCA

Stp58 CCTAAAGTTGGAAACCAGCAAGAGGATAAAACTACAGTTGGTACAACTGAAAAAGCACCA

************************************************************

GTBN_1-4-5-12 TTACCAATTGCGCAACCACTAGTTAAATTACCACAAATTGGGACTCAAGGTAAAATTGTA

Stp25 TTACCAATTGCGCAACCACTAGTTAAATTACCACAAATTGGGACTCAAGGTAAAATTGTA

Stp58 TTACCAATTGCGCAACCACTAGTTAAATTACCACAAATTGGGACTCAAGGTAAAATTGTA

************************************************************

GTBN_1-4-5-12 GAAGGGCCAAAATACCCAACGATGGAACAGCACACAATCTATGGTGAAATTGTAAATGGT

Stp25 GAAGGGCCAAAATACCCAACGATGGAACAGCACACAATCTATGGTGAAATTGTAAAAGGT

Stp58 GAAGGGCCAAAATACCCAACGATGGAACAGCACACAATCTATGGTGAAATTGTAAAAGGT

********************************************************:***

GTBN_1-4-5-12 CCCGACTATCTAACGATGGAAAATAAAACGTTACAAGGTGAAATCGTTCAAGGTCCTGAT

Stp25 CCCGACTATCTAACGATGGAAAATAAAACGTTACAAGGTGAAATCGTTCAAGGTCCAGAT

Stp58 CCCGACTATCTAACGATGGAAAATAAAACGTTACAAGGTGAAATCGTTCAAGGTCCAGAT

********************************************************:***

GTBN_1-4-5-12 TTCCCAACAATGGAACAAAACAGACCATCTTTAAGCGATAATTATACTCAACCGACGACA

Stp25 TTCCCAACAATGGAACAAAACAGACCAGCACTAAGCGATAATTATACAAACCCAACGTTA

Stp58 TTCCCAACAATGGAACAAAACAGACCAGCACTAAGCGATAATTATACAAACCCAACGTTA

*************************** *: ****************:.*.**.***: *

GTBN_1-4-5-12 CCGAACCCTATTTTAGAAGGTCTTGAAGGTAGCTCATCTAAACTCGAAATAAAACCACAA

Stp25 ACGAACCCTATTTTAAAAGGTATTGAAGGAAACTCAACTAATCTTGAAATAAAACCACAA

Stp58 ACGAACCCTATTTTAAAAGGTATTGAAGGAAACTCAACTAATCTTGAAATAAAACCACAA

.**************.*****.*******:*.****:****:** ***************

GTBN_1-4-5-12 GGTACTGAATCAACGTTGAAAGGTATTCAAGGAGAATCAAGTGATATTGAAGTTAAACCT

Stp25 GGTACTGAATCAACGTTGAAAGGTATTCAAGGAGAATCAAGTGATATTGAAGTTAAACCT

Stp58 GGTACTGAATCAACGTTGAAAGGTATTCAAGGAGAATCAAGTGATATTGAAGTTAAACCT

************************************************************

GTBN_1-4-5-12 CAAGCAACTGAAACAACAGAAGCATCACATTATCCAGCGAGACCGCAATTTAACAAAACA

Stp25 CAAGCAACTGAAACAACAGAAGCATCACATTATCCAGCGAGACCGCAATTTAACAAAACA

Stp58 CAAGCAACTGAAACAACAGAAGCATCACATTATCCAGCGAGACCGCAATTTAACAAAACA

************************************************************

GTBN_1-4-5-12 CCTAAGTATGTGAAATATAGAGATGCTGGTACAGGTATTCGTGAATACAACGATGGAACA

Stp25 CCTAAGTATGTGAAATATAGAGATGCTGGTACAGGTATCCGTGAATACAACGATGGAACA

Stp58 CCTAAGTATGTGAAATATAGAGATGCTGGTACAGGTATCCGTGAATACAACGATGGAACA

************************************** *********************

GTBN_1-4-5-12 TTTGGATATGAAGCGAGACCAAGATTCAACAAGCCAAGTGAAACAAATGCATACAACGTA

Stp25 TTTGGATATGAAGCGAGACCAAGATTCAACAAGCCATCAGAAACAAACGCATACAACGTA

Stp58 TTTGGATATGAAGCGAGACCAAGATTCAACAAGCCATCAGAAACAAACGCATACAACGTA

************************************: :******** ************

GTBN_1-4-5-12 ACGACAAATCAAGATGGCACAGTATCATACGGCGCCCGCCCGACACAAAACAAG------

Stp25 ATGACAAATCAAGATGGCACAGTATCATACGGCGCCCGCCCAACACAAAACAAGGCATCA

Stp58 ATGACAAATCAAGATGGCACAGTATCATACGGCGCCCGCCCAACACAAAACAAGGCATCA

* ***************************************.************

GTBN_1-4-5-12 ------------------------------------------------------------

Stp25 GAAACAAACGCATATAACGTAACAACACATGCAAACGGCCAAGTATCATACGGAGCTCGC

Stp58 GAAACAAACGCATATAACGTAACAACACATGCAAACGGCCAAGTATCATACGGAGCTCGC

GTBN_1-4-5-12 ---------------CCAAGCGAAACGAATGCATATAACGTAACAGCACACGGAAATGGT

Stp25 CCAACACAAAAGAAGCCAAGCGAAACAAATGCATATAACGTAACAACACATGCAAACGGC

Stp58 CCAACACAAAAGAAGCCAAGCGAAACAAATGCATATAACGTAACAACACATGCAAACGGC

***********.******************.**** * *** **

GTBN_1-4-5-12 CAAGTGTCATACGGCGCTCGTCCGACATACAAGAAGCCAAGCGAAACAAATGCATATAAC

Stp25 CAAGTATCATATGGCGCCCGCCCGACATACAAGAAGCCAAGTGAAACAAATGCATATAAC

Stp58 CAAGTATCATATGGCGCCCGCCCGACATACAAGAAGCCAAGTGAAACAAATGCATATAAC

*****.***** ***** ** ******************** ******************

GTBN_1-4-5-12 GTAACAACACATGCAAATGGTCAAGTATCATACGGAGCTCGCCCAACACAAAACAAGCCA

Stp25 GTAACAACACATGCAAATGGCCAAGTATCATATGGGGCTCGCCCAACACAAAACAAGCCA

Stp58 GTAACAACACATGCAAATGGCCAAGTATCATATGGGGCTCGCCCAACACAAAACAAGCCA

******************** *********** **.************************

GTBN_1-4-5-12 AGCGAAACAAACGCATATAACGTAACAACACATGCAAACG--------------------

Stp25 AGCAATACAAACGCATATAACGTAACAACACATGCAAACGGCCAAGTATCATATGGGGCT

Stp58 AGCAATACAAACGCATATAACGTAACAACACATGCAAACGGCCAAGTATCATATGGGGCT

***.*:**********************************

GTBN_1-4-5-12 ------------------------------------------------------------

Stp25 CGCCCGACACAAAACAAGGCATCAGAAACAAACGCATATAACGTAACAACACATGCAAAC

Stp58 CGCCCGACACAAAACAAGGCATCAGAAACAAACGCATATAACGTAACAACACATGCAAAC

GTBN_1-4-5-12 -GTCAAGTGTCATACGGAGCTCGCCCGACACAAAACAAGCCAAGCAAAACAAACGCATAT

Stp25 GGCCAAGTATCATACGGAGCTCGCCCGACACAAAACAAGCCAAGCGAAACAAACGCATAT

Stp58 GGCCAAGTATCATACGGAGCTCGCCCGACACAAAACAAGCCAAGCGAAACAAACGCATAT

* *****.************************************.**************

GTBN_1-4-5-12 AACGTAACAACACACGGAAATGGCACAGTATCATATGGCGCTCGCCCGACACAAAACAAG

Stp25 AACGTAACAACACACGGAAACGGTCAAGTGTCATACGGCGCTCGTCCGACATACAACAAG

Stp58 AACGTAACAACACACGGAAACGGTCAAGTGTCATACGGCGCTCGTCCGACATACAACAAG

******************** ** ..***.***** ******** ****** *.******

GTBN_1-4-5-12 CCAAGTAAAACAAATGCATATAACGTAACAACACATGCAGATGGTACTGCGACATATGGT

Stp25 CCAAGTAAAACAAATGCATACAATGTAACAACACATGCAGATGGTACTGCGACATATGGT

Stp58 CCAAGTAAAACAAATGCATACAATGTAACAACACATGCAGATGGTACTGCGACATATGGT

******************** ** ************************************

GTBN_1-4-5-12 CCTAGAGTAACAAAATAA

Stp25 CCTAGAGTAACAAAATAA

Stp58 CCTAGAGTAACAAAATAA

******************

**C)**

Stp58 MKKQIISLGALAVASSLFTWDNKADAIVTKDYSKESRVNENSKYDSPMSNWYYWGKVKSL

Stp25 MKKQIISLGALAVASSLFTWDNKADAIVTKDYSKESRVNENSKYDSPMSNWYYWGKVKSL

GTBN_1-4-5-12 MKKQIISLGALAVASSLFTWDNKADAIVTKDYSKESRVKENSKYDSPMSNWYYWGKVKSL

**************************************:*********************

Stp58 ESQFADAIDIIEDYQYGEKEYKDAKDKLMTRILGEDQYLLKKKIEEYKQYRERYLKAGLS

Stp25 ESQFADAIDIIEDYQYGEKEYKDAKDKLMTRILGEDQYLLKKKIEEYKQYRERYLKAGLS

GTBN_1-4-5-12 ESQFADAIDIIEDYQYGEKEYKDAKDKLMTRILGEDQYLLKKKIEEYKQYRERYLKAGLS

************************************************************

Stp58 PVKFYDYNLYDFTMKEYNDIHQSLKDAVEEFYQEVKHIQSKNSDLQTYDKKTEDKETDNV

Stp25 PVKFYDYNLYDFTMKEYNDIHQSLKDAVEEFYQEVKHIQSKNSDLQTYDKKTEDKETDNV

GTBN_1-4-5-12 PVKFYDYNLYDFTMKEYNDIHQSLKDAVEEFYQEVKHIQSKNSDLQTYDKKTEDKETDNV

************************************************************

Stp58 YSLVSEIDTIVATYYGDKNHGEHAKELRAKLDIILGEEKKPNRITNERIRKEMTDDLNSI

Stp25 YSLVSEIDTIVATYYGDKNHGEHAKELRAKLDIILGEEKKPNRITNERIRKEMTDDLNSI

GTBN_1-4-5-12 YSLVSEIDTIVATYYGDKNHGEHAKELRAKLDIILGEEKSQIE-LMNVLEKK-LMI-ILL

***************************************. . : :.*: :

Stp58 IDDFFMETGQNRPVKITKYNPNIHSPKDNKESFDKLVEETKKAVKEADESWKTKTVKTYG

Stp25 IDDFFMETGQNRPVKITKYNPNIHSPKDNKESFDKLVEETKKAVKEADESWKTKTVKTYG

GTBN_1-4-5-12 SMTSLWKLGKTDRLKSLNIIQIFIALKITKKASIN-------------------------

: : *:. :* : : : * .*:: :

Stp58 ETETKAHVVKEEKKVEEPQLPKVGNQQEDKTTVGTTEKAPLPIAQPLVKLPQIGTQGKIV

Stp25 ETETKAHVVKEEKKVEEPQLPKVGNQQEDKTTVGTTEKAPLPIAQPLVKLPQIGTQGKIV

GTBN_1-4-5-12 ------------------------------------------------------------

Stp58 EGPKYPTMEQHTIYGEIVKGPDYLTMENKTLQGEIVQGPDFPTMEQNRPALSDNYTNPTL

Stp25 EGPKYPTMEQHTIYGEIVKGPDYLTMENKTLQGEIVQGPDFPTMEQNRPALSDNYTNPTL

GTBN_1-4-5-12 ------------------------------------------------------------

Stp58 TNPILKGIEGNSTNLEIKPQGTESTLKGIQGESSDIEVKPQATETTEASHYPARPQFNKT

Stp25 TNPILKGIEGNSTNLEIKPQGTESTLKGIQGESSDIEVKPQATETTEASHYPARPQFNKT

GTBN_1-4-5-12 ------------------------------------------------------------

Stp58 PKYVKYRDAGTGIREYNDGTFGYEARPRFNKPSETNAYNVMTNQDGTVSYGARPTQNKAS

Stp25 PKYVKYRDAGTGIREYNDGTFGYEARPRFNKPSETNAYNVMTNQDGTVSYGARPTQNKAS

GTBN_1-4-5-12 ------------------------------------------------------------

Stp58 ETNAYNVTTHANGQVSYGARPTQKKPSETNAYNVTTHANGQVSYGARPTYKKPSETNAYN

Stp25 ETNAYNVTTHANGQVSYGARPTQKKPSETNAYNVTTHANGQVSYGARPTYKKPSETNAYN

GTBN_1-4-5-12 ------------------------------------------------------------

Stp58 VTTHANGQVSYGARPTQNKPSNTNAYNVTTHANGQVSYGARPTQNKASETNAYNVTTHAN

Stp25 VTTHANGQVSYGARPTQNKPSNTNAYNVTTHANGQVSYGARPTQNKASETNAYNVTTHAN

GTBN_1-4-5-12 ------------------------------------------------------------

Stp58 GQVSYGARPTQNKPSETNAYNVTTHGNGQVSYGARPTYNKPSKTNAYNVTTHADGTATYG

Stp25 GQVSYGARPTQNKPSETNAYNVTTHGNGQVSYGARPTYNKPSKTNAYNVTTHADGTATYG

GTBN_1-4-5-12 ------------------------------------------------------------

Stp58 PRVTK

Stp25 PRVTK

GTBN_1-4-5-12 -----
